# Supplementary material for: The Flavoproteome of the Model Plant Arabidopsis thaliana
Source: Int J Mol Sci. 2020 Jul 28;21(15):5371. doi: 10.3390/ijms21155371 (PMC7432721; doi:10.3390/ijms21155371)
Supplement: Supplementary file 1 [file ijms-21-05371-s001.zip › Table S3.pdf]

Table S3. Flavoproteom protein sequence similarities. Depicting how many partner each flavoprotein has with a sequence similarity greater than 30 %, 50 % and 60 %.

	30%	50%	60%
AT1G01580.1	2	2	0
AT1G01590.1	2	2	1
AT1G01980.1	26	4	2
AT1G03990.1	0	0	0
AT1G04180.1	10	3	1
AT1G04400.2	1	0	0
AT1G04580.1	3	2	0
AT1G04610.1	10	4	1
AT1G04620.1	0	0	0
AT1G06290.1	1	1	1
AT1G06310.1	1	1	1
AT1G06820.1	0	0	0
AT1G07180.1	1	1	1
AT1G09090.2	9	0	0
AT1G09400.1	0	0	0
AT1G11770.1	26	4	2
AT1G12130.1	14	11	1
AT1G12140.1	14	12	7
AT1G12160.1	14	9	0
AT1G12200.1	14	12	3
AT1G12370.2	0	0	0
AT1G12570.1	4	0	0
AT1G14185.1	3	1	1
AT1G14190.1	1	1	1
AT1G15020.2	0	0	0
AT1G17990.1	1	1	1
AT1G18020.1	1	1	1
AT1G19230.2	9	0	0
AT1G19250.1	1	0	0
AT1G20020.1	1	1	0
AT1G21430.1	6	0	0
AT1G23020.2	2	2	1
AT1G24340.1	0	0	0
AT1G26380.1	26	5	5
AT1G26390.1	26	5	5
AT1G26400.1	26	5	5
AT1G26410.1	26	5	5
AT1G26420.1	26	5	5
AT1G30510.2	2	1	0
AT1G30700.1	26	0	0
AT1G30710.1	26	1	1
AT1G30720.1	26	1	1
AT1G30730.1	26	1	1
AT1G30740.1	26	4	2
AT1G30760.1	26	3	1
AT1G32300.1	5	4	4

AT1G34575.1	26	1	1
AT1G37130.1	1	1	1
AT1G48030.1	1	1	1
AT1G48605.1	1	1	1
AT1G48850.1	0	0	0
AT1G48910.1	10	0	0
AT1G49880.1	0	0	0
AT1G50940.1	0	0	0
AT1G55930.1	1	1	1
AT1G58440.1	5	1	1
AT1G62540.1	14	11	4
AT1G62560.1	14	11	4
AT1G62570.1	14	11	4
AT1G62580.1	14	5	2
AT1G62600.1	14	12	3
AT1G62620.1	14	10	3
AT1G62830.1	0	0	0
AT1G63340.1	14	11	2
AT1G63370.1	14	10	3
AT1G63390.1	0	0	0
AT1G63940.2	0	0	0
AT1G64060.1	9	1	0
AT1G65840.1	2	2	0
AT1G65860.1	14	11	4
AT1G68050.1	2	1	0
AT1G72280.1	0	0	0
AT1G72970.1	4	0	0
AT1G73050.1	1	0	0
AT1G75200.1	0	0	0
AT1G75450.1	5	0	0
AT1G76680.2	0	0	0
AT1G76690.1	1	0	0
AT1G77760.1	1	1	1
AT2G01270.1	0	0	0
AT2G02710.1	0	0	0
AT2G06050.2	1	0	0
AT2G13440.1	0	0	0
AT2G17420.1	1	1	1
AT2G18450.1	1	1	1
AT2G18915.2	2	1	0
AT2G19500.1	3	1	0
AT2G20360.1	0	0	0
AT2G20800.1	3	1	0
AT2G22830.1	2	1	0
AT2G24580.1	0	0	0
AT2G27150.1	3	1	0
AT2G29720.1	1	1	1
AT2G29990.1	1	1	1
AT2G33230.1	10	3	1
AT2G34790.1	26	4	1

AT2G34810.1	26	0	0
AT2G35660.1	1	1	1
AT2G35690.1	0	0	0
AT2G38960.3	0	0	0
AT2G41220.1	2	1	1
AT2G41510.1	2	0	0
AT2G41680.1	0	0	0
AT2G43020.1	2	2	1
AT2G43400.1	0	0	0
AT2G44160.1	1	1	1
AT2G46580.1	0	0	0
AT2G46740.1	5	4	4
AT2G46750.1	5	4	4
AT2G46760.1	5	4	4
AT2G47590.1	0	0	0
AT3G02280.1	0	0	0
AT3G06690.1	0	0	0
AT3G06810.1	0	0	0
AT3G09940.1	2	2	1
AT3G10370.1	0	0	0
AT3G10390.1	0	0	0
AT3G13070.1	1	1	1
AT3G13682.1	0	0	0
AT3G14130.1	3	1	1
AT3G14150.1	3	1	1
AT3G14415.2	4	2	1
AT3G14420.1	4	2	1
AT3G15620.1	0	0	0
AT3G16950.2	1	1	0
AT3G17240.1	1	1	1
AT3G18030.1	1	1	1
AT3G19820.1	0	0	0
AT3G23410.1	0	0	0
AT3G24170.1	1	0	0
AT3G27820.1	1	0	0
AT3G27890.1	0	0	0
AT3G30775.1	1	1	1
AT3G43600.1	3	1	0
AT3G44190.1	1	1	1
AT3G45300.1	0	0	0
AT3G45780.1	1	0	0
AT3G45810.1	9	1	1
AT3G47930.1	0	0	0
AT3G48560.1	0	0	0
AT3G49640.1	0	0	0
AT3G51840.1	0	0	0
AT3G52880.2	3	2	1
AT3G54660.1	1	0	0
AT3G56060.1	3	1	0
AT3G56840.1	0	0	0

AT3G59050.1	2	2	1
AT3G59970.3	1	1	1
AT3G63440.1	5	0	0
AT3G63510.1	1	1	1
AT4G01690.1	0	0	0
AT4G05020.2	2	0	0
AT4G05390.1	1	1	0
AT4G08920.1	1	0	0
AT4G11230.1	9	1	0
AT4G13260.1	9	2	0
AT4G14210.1	0	0	0
AT4G15760.1	0	0	0
AT4G16155.1	1	1	0
AT4G16310.1	0	0	0
AT4G16760.1	0	0	0
AT4G18360.1	2	2	0
AT4G19380.1	0	0	0
AT4G20800.1	26	5	5
AT4G20820.1	26	2	0
AT4G20830.1	26	4	1
AT4G20840.1	26	4	1
AT4G20860.1	26	1	1
AT4G21490.1	3	2	0
AT4G24520.1	1	1	1
AT4G25090.1	9	1	1
AT4G27270.1	3	1	0
AT4G28220.1	2	1	0
AT4G28570.1	0	0	0
AT4G28720.1	10	4	0
AT4G29720.1	0	0	0
AT4G29740.2	5	1	0
AT4G30210.1	1	1	1
AT4G32360.1	0	0	0
AT4G32540.1	9	0	0
AT4G34890.1	1	1	1
AT4G34900.1	1	1	1
AT4G35460.1	1	1	1
AT4G36400.1	0	0	0
AT4G36750.1	2	0	0
AT4G37760.1	5	2	1
AT4G38540.1	1	1	1
AT4G38890.1	0	0	0
AT5G03630.1	2	2	2
AT5G04140.2	2	1	1
AT5G05320.1	1	1	1
AT5G06580.1	0	0	0
AT5G07390.1	9	2	0
AT5G07800.1	14	1	1
AT5G08530.1	0	0	0
AT5G08740.1	0	0	0

AT5G11320.1	9	3	0
AT5G11330.1	0	0	0
AT5G11540.1	5	0	0
AT5G13700.1	0	0	0
AT5G14220.1	0	0	0
AT5G14760.1	0	0	0
AT5G17770.1	0	0	0
AT5G20080.1	0	0	0
AT5G20960.1	3	2	0
AT5G21482.1	3	0	0
AT5G22140.1	1	1	1
AT5G23300.1	0	0	0
AT5G23980.1	0	0	0
AT5G23990.1	0	0	0
AT5G24140.1	4	2	1
AT5G24150.1	4	2	2
AT5G24160.1	4	2	1
AT5G24850.1	0	0	0
AT5G25620.2	10	2	0
AT5G38710.1	1	1	1
AT5G43430.1	0	0	0
AT5G43890.1	9	5	1
AT5G44360.1	26	1	1
AT5G44380.1	26	3	2
AT5G44390.1	26	4	2
AT5G44400.1	26	4	2
AT5G44410.1	26	2	1
AT5G44440.1	26	2	1
AT5G45180.1	1	0	0
AT5G47910.1	9	1	0
AT5G47970.1	1	1	1
AT5G49730.1	1	1	1
AT5G49740.1	1	1	1
AT5G49970.1	0	0	0
AT5G50160.1	0	0	0
AT5G51060.1	9	2	1
AT5G51950.1	4	1	0
AT5G53460.1	2	0	0
AT5G54500.2	2	1	0
AT5G56470.1	0	0	0
AT5G56490.1	5	4	4
AT5G56970.1	3	0	0
AT5G57360.2	2	2	0
AT5G58140.2	1	0	0
AT5G58800.1	3	0	0
AT5G60010.1	9	1	1
AT5G61290.1	14	1	1
AT5G63910.1	0	0	0
AT5G65110.1	0	0	0
AT5G66190.1	2	1	0

AT5G66760.1	1	1	1
AT5G67030.1	0	0	0
AT5G67220.1	0	0	0
