# Supplementary material for: The Flavoproteome of the Model Plant Arabidopsis thaliana
Source: Int J Mol Sci. 2020 Jul 28;21(15):5371. doi: 10.3390/ijms21155371 (PMC7432721; doi:10.3390/ijms21155371)
Supplement: Supplementary file 1 [file ijms-21-05371-s001.zip › Table S4 .pdf]

Table S4 Summary structure prediction of the 38 genes categorized in evidence code 3 with the Phyre 2 web server.

# Description	Job id	Hit	Confidence (%)	Sequence identity (%)	Alignment coverage	Hit info 1
AT1G09400.1	e42a1a5ab680e0f1	d1gwja_	100	43	99	TIM beta/alpha-barr
AT1G12200.1	5bf852ba2fc3aa75	c6wpuA_	100	60	95	
AT1G12570.1	b31b32eaf70c5e29	c1ju2A_	100	41	95	
AT1G14185.1	c54d62a5c9d6c4b9	c1ju2A_	100	39	97	
AT1G14190.1	f464783a9da53908	c1ju2A_	100	40	95	
AT1G17990.1	2da61e11fe8bb162	c6qkgB_	100	27	98	
AT1G18020.1	75388141d464da53	c6qkgB_	100	27	98	
AT1G24340.1	b836d7b4902631c4	c6u0pC_	100	27	93	
AT1G55930.1	26f7e1f28b1b0dd3	c4hg0A_	100	30	40	
AT1G63340.1	62339ef6c54bf635	c6se3E_	100	29	95	
AT1G63390.1	208e0c724a6d7743	c6se3E_	99,8	35	93	
AT1G75200.1	24a6864c4e3b81db	c2z2uA_	100	35	48	
AT2G02710.1	66b4174cd5c62dda	c6hmjD_	100	19	85	
AT2G13440.1	88c19d469e05682e	c3cp8C_	100	47	85	
AT2G20360.1	77b8090b4fd0680c	c1z45A_	100	19	78	
AT2G29720.1	53ea3eedbc4b5507	c4bk2A_	100	19	89	
AT2G35660.1	8b7fc99e64d4d6bb	c6c6rA_	100	18	84	
AT2G44160.1	455494909bb887c8	c6fcxA_	100	45	99	
AT2G47590.1	2dac5fae1f4c6c80	c2j4dA_	100	36	77	
AT3G06690.1	6153d12d5272176b	d2ddha2	100	19	83	Bromodomain-like
AT3G13070.1	88483729d729af6a	c4hg0A_	100	31	40	
AT3G14130.1	4e2375a892bd56b9	c6dvhF_	100	34	96	
AT3G14150.1	6de950eec66c27d4	c6dvhF_	100	34	96	
AT3G49640.1	ca60065fc296601e	c4xp7A_	100	46	96	
AT3G56060.1	e32765e6e4c9ecaa		100	20	92	
AT3G63510.1	69dc4230cfbcb0e2	c3b0vD_	100	36	78	
AT4G27270.1	4fde53250665b3fe	c4lafB_	100	46	97	
AT4G36750.1	f53fcd768c35b35d	c5mp4C_	100	46	71	

AT4G38890.1	398663dcdcaa05c8	d1vhna_	100	26	47	TIM beta/alpha-barr
AT5G07800.1	5562e5fed485aa8e	c2vq7B_	100	27	92	
AT5G11330.1	cf830396d3fd8dcc	c6pvhA_	100	19	97	
AT5G20080.1	740cc95b13d700cb	c4p6vF_	100	22	96	
AT5G45180.1	4f8b368cb39c5045	c6semA_	100	30	97	
AT5G47970.1	f107bed3b307d429	c3b0vD_	100	32	82	
AT5G51950.1	e6f8594d32f129bd	c1ju2A_	100	42	94	
AT5G56470.1	956fd06c8ae981d4	d2i0ka1	100	19	58	Ferredoxin-like
AT5G58800.1	79df30d02ab8a13a	c4lafB_	100	43	96	
AT5G67220.1	f2a100b241673faa	d1vhna_	100	20	73	TIM beta/alpha-barr

Hit info 2	Hit info 3
FMN-linked oxidoreductases	FMN-linked oxidoreductases
PDB header:flavoprotein, oxidoreductase	Chain: A: PDB Molecule:flavin-containing monooxygenase;
PDB header:lyase	Chain: A: PDB Molecule:hydroxynitrile lyase;
PDB header:lyase	Chain: A: PDB Molecule:hydroxynitrile lyase;
PDB header:lyase	Chain: A: PDB Molecule:hydroxynitrile lyase;
PDB header:flavoprotein	Chain: B: PDB Molecule:ncr a;
PDB header:flavoprotein	Chain: B: PDB Molecule:ncr a;
PDB header:flavoprotein	Chain: C: PDB Molecule:2,4-dichlorophenol 6-monooxygenase;
PDB header:transport protein	Chain: A: PDB Molecule:magnesium and cobalt efflux protein corc;
PDB header:membrane protein	Chain: E: PDB Molecule:ancestral flavin-containing monooxygenase (fmo) 3-6;
PDB header:membrane protein	Chain: E: PDB Molecule:ancestral flavin-containing monooxygenase (fmo) 3-6;
PDB header:metal binding protein	Chain: A: PDB Molecule:upf0026 protein mj0257;
PDB header:signaling protein	Chain: D: PDB Molecule:putative pas/pac sensor protein;
PDB header:oxidoreductase	Chain: C: PDB Molecule:trna uridine 5-carboxymethylaminomethyl
PDB header:isomerase	Chain: A: PDB Molecule:gal10 bifunctional protein;
PDB header:oxidoreductase	Chain: A: PDB Molecule:probable salicylate monooxygenase;
PDB header:oxidoreductase/oxidoreductase inhibitor	Chain: A: PDB Molecule:squalene monooxygenase;
PDB header:oxidoreductase	Chain: A: PDB Molecule:methylenetetrahydrofolate reductase;
PDB header:dna binding protein	Chain: A: PDB Molecule:cryptochrome dash;
Acyl-CoA dehydrogenase C-terminal domain-like	acyl-CoA oxidase C-terminal domains
PDB header:transport protein	Chain: A: PDB Molecule:magnesium and cobalt efflux protein corc;
PDB header:oxidoreductase	Chain: F: PDB Molecule:lactate 2-monooxygenase;
PDB header:oxidoreductase	Chain: F: PDB Molecule:lactate 2-monooxygenase;
PDB header:oxidoreductase	Chain: A: PDB Molecule:trna-dihydrouridine(20) synthase [nad(p)+]-like;
PDB header:oxidoreductase	Chain: G: PDB Molecule:alcohol oxidase 1;
PDB header:oxidoreductase/rna	Chain: D: PDB Molecule:trna-dihydrouridine synthase;
PDB header:oxidoreductase	Chain: B: PDB Molecule:nad(p)h dehydrogenase (quinone);
PDB header:oxidoreductase	Chain: C: PDB Molecule:protoplast secreted protein 2;

FMN-linked oxidoreductases	FMN-linked oxidoreductases
PDB header:oxidoreductase	Chain: B: PDB Molecule:flavin-containing monooxygenase;
PDB header:biosynthetic protein	Chain: A: PDB Molecule:fad monooxygenase;
PDB header:oxidoreductase	Chain: F: PDB Molecule:na(+)-translocating nadh-quinone reductase subunit f;
PDB header:membrane protein	Chain: A: PDB Molecule:ancestral flavin-containing monooxygenase (fmo) 2;
PDB header:oxidoreductase/rna	Chain: D: PDB Molecule:trna-dihydrouridine synthase;
PDB header:lyase	Chain: A: PDB Molecule:hydroxynitrile lyase;
FAD-linked oxidases, C-terminal domain	Cholesterol oxidase
PDB header:oxidoreductase	Chain: B: PDB Molecule:nad(p)h dehydrogenase (quinone);
FMN-linked oxidoreductases	FMN-linked oxidoreductases

Table S4 flavin binding prediction of the 38 genes

Input	Entry	Status
AT1G09400	A0A1P8AT!	unreviewed
AT1G09400	Q8GYA3	reviewed
AT1G12200	A0A1P8AVI	unreviewed
AT1G12200	Q9FWW9	reviewed
AT1G12570	A0A5S9U1(	unreviewed
AT1G12570	Q66GI5	unreviewed
AT1G12570	Q9LN93	unreviewed
AT1G14185	Q9XI69	unreviewed
AT1G14190	A0A1P8API	unreviewed
AT1G14190	Q9XI68	unreviewed
AT1G17990	C0Z3F5	unreviewed
AT1G17990	F4I949	unreviewed
AT1G17990	P0DI08	reviewed
AT1G18020	P0DI09	reviewed
AT1G18020	Q56XI3	unreviewed
AT1G24340	Q8GYJ7	unreviewed
AT1G55930	A0A1P8AR!	unreviewed
AT1G55930	Q84R21	reviewed
AT1G63340	Q9C8T8	reviewed
AT1G63390	Q9SH25	reviewed
AT1G75200	Q56XF4	unreviewed
AT1G75200	Q8RXN5	reviewed
AT2G02710	A0A1P8AZI	unreviewed
AT2G02710	A0A2H1ZE:	unreviewed
AT2G02710	O64511	reviewed
AT2G13440	Q9SHS2	unreviewed
AT2G20360	Q9SK66	reviewed
AT2G29720	O82384	unreviewed
AT2G35660	F4IKT4	unreviewed
AT2G35660	Q9ZQN9	unreviewed

AT2G44160	O80585	reviewed
AT2G47590	Q8LB72	reviewed
AT3G06690	P0CZ24	reviewed
AT3G13070	Q9LK65	reviewed
AT3G14130	A0A1I9LRL	unreviewe
AT3G14130	A0A1I9LRL	unreviewe
AT3G14130	Q9LJH5	reviewed
AT3G14150	A0A178VH	unreviewe
AT3G14150	A0A178VL	unreviewe
AT3G14150	A0A178VM	unreviewe
AT3G14150	Q24JJ8	reviewed
AT3G49640	A0A1I9LM	unreviewe
AT3G49640	A0A1I9LM	unreviewe
AT3G49640	A0A1I9LM	unreviewe
AT3G49640	F4IY04	unreviewe
AT3G49640	Q8GWM4	unreviewe
AT3G49640	Q9SCJ5	unreviewe
AT3G56060	A0A5S9XLF	unreviewe
AT3G56060	Q93ZK1	unreviewe
AT3G56060	Q9LYN5	unreviewe
AT3G63510	A8MRX7	unreviewe
AT3G63510	C0Z2A1	unreviewe
AT3G63510	Q8H128	unreviewe
AT4G27270	A0A1P8B7	unreviewe
AT4G27270	A0A1P8B7	unreviewe
AT4G27270	Q6NQE2	reviewed
AT4G36750	O23207	reviewed
AT4G36750	Q941C6	unreviewe
AT4G38890	Q9T0J6	reviewed
AT5G07800	Q9FF12	reviewed
AT5G11330	A0A178UA	unreviewe
AT5G11330	F4JWH3	unreviewe

AT5G11330	Q9LFM4	unreviewe(
AT5G20080	P83291	reviewed
AT5G20080	Q29Q36	unreviewe(
AT5G45180	Q9FKE7	reviewed
AT5G47970	A0A1P8BFE	unreviewe(
AT5G47970	Q9FI41	unreviewe(
AT5G51950	A0A1P8BH(	unreviewe(
AT5G51950	F4KEQ8	unreviewe(
AT5G51950	Q56ZG6	unreviewe(
AT5G51950	Q94KD2	unreviewe(
AT5G56470	Q9FM84	reviewed
AT5G58800	A0A178U9(	unreviewe(
AT5G58800	Q9LUX9	reviewed
AT5G67220	Q56ZN4	unreviewe(
AT5G67220	Q9FH91	unreviewe(

categorized in evidence code 3 with UniProt database.

Protein names
FMN-linked oxidoreductases superfamily protein<br>Putative 12-oxophytodienoate reductase-like protein 1 (EC 1.3.1.-)
Flavin-containing monooxygenase (EC 1.-.-.-)<br>Flavin-containing monooxygenase FMO GS-OX-like 2 (EC 1.8.-.-) (Flavin-monooxygenase glucosinolate S-oxygenase-like 2)
Uncharacterized protein<br>At1g12570 (Glucose-methanol-choline (GMC) oxidoreductase family protein)<br>F5O11.31
At1g14180/F7A19_27 (F7A19.27 protein) (Glucose-methanol-choline (GMC) oxidoreductase family protein)
Glucose-methanol-choline (GMC) oxidoreductase family protein<br>F7A19.28 protein (Glucose-methanol-choline (GMC) oxidoreductase family protein)
AT1G17990 protein<br>FMN-linked oxidoreductases superfamily protein<br>Putative 12-oxophytodienoate reductase-like protein 2A (EC 1.3.1.-)
Putative 12-oxophytodienoate reductase-like protein 2B (EC 1.3.1.-)<br>Uncharacterized protein At1g18020
FAD/NAD(P)-binding oxidoreductase family protein
CBS domain-containing protein / transporter associated domain-containing protein<br>DUF21 domain-containing protein At1g55930, chloroplastic (CBS domain-containing protein CBSDUFCH2)
Putative flavin-containing monooxygenase FMO GS-OX-like 10 (EC 1.8.-.-) (Putative flavin-monooxygenase glucosinolate S-oxygenase-like 10)
Putative flavin-containing monooxygenase FMO GS-OX-like 11 (EC 1.8.-.-) (Putative flavin-monooxygenase glucosinolate S-oxygenase-like 11)
Uncharacterized protein At1g75200 (Fragment)<br>S-adenosyl-L-methionine-dependent tRNA 4-demethylwyosine synthase (EC 4.1.3.44) (tRNA wybutosine-synthesizing protein 1 homolog)
PAS/LOV protein B<br>PAS/LOV protein B<br>Protein TWIN LOV 1
Glucose-inhibited division family A protein (Similar to glucose inhibited division protein A from prokaryotes)
NADH dehydrogenase [ubiquinone] 1 alpha subcomplex subunit 9, mitochondrial
FAD/NAD(P)-binding oxidoreductase family protein (Putative monooxygenase)
FAD/NAD(P)-binding oxidoreductase family protein<br>FAD/NAD(P)-binding oxidoreductase family protein (Putative monooxygenase)

Methylenetetrahydrofolate reductase 2 (AtMTHFR2) (EC 1.5.1.20)
Blue-light photoreceptor PHR2
Putative acyl-coenzyme A oxidase At3g06690 (Acyl-CoA oxidase) (EC 1.3.3.6)
Putative DUF21 domain-containing protein At3g13070, chloroplastic (CBS domain-containing protein CBSDUFCH1)
Aldolase-type TIM barrel family protein<br>Aldolase-type TIM barrel family protein<br>Peroxisomal (S)-2-hydroxy-acid oxidase GLO4 (EC 1.1.3.15) (Glycolate oxidase 4) (AtGLO4) (GOX 4) (Short chain alpha-hydroxy acid oxidase GLO4)
Aldolase-type TIM barrel family protein<br>HAOX2<br>Aldolase-type TIM barrel family protein<br>Peroxisomal (S)-2-hydroxy-acid oxidase GLO3 (EC 1.1.3.15) (Glycolate oxidase 3) (AtGLO3) (GOX 3) (Short chain alpha-hydroxy acid oxidase GLO3)
Aldolase-type TIM barrel family protein<br>Aldolase-type TIM barrel family protein<br>tRNA-dihydrouridine synthase (EC 1.3.1.-)<br>Aldolase-type TIM barrel family protein<br>Aldolase-type TIM barrel family protein<br>Uncharacterized protein T9C5.230
Uncharacterized protein<br>AT3g56060/F18O21_20 (Glucose-methanol-choline (GMC) oxidoreductase family protein)<br>ADHESION OF CALYX EDGES-like protein
tRNA-dihydrouridine synthase (EC 1.3.1.-)<br>AT3G63510 protein<br>FMN-linked oxidoreductases superfamily protein
Quinone reductase family protein<br>Quinone reductase family protein<br>Probable NAD(P)H dehydrogenase (quinone) FQR1-like 1 (EC 1.6.5.2)
Probable NAD(P)H dehydrogenase (quinone) FQR1-like 2 (EC 1.6.5.2)<br>At4g36690/C7A10_610 (C7A10_610/C7A10_610)
tRNA-dihydrouridine(47) synthase [NAD(P)(+)]-like (EC 1.3.1.-) (Zinc finger CCCH domain-containing protein 50) (AtC3H50) (tRNA-dihydrouridine synthas
Flavin-containing monooxygenase FMO GS-OX-like 9 (EC 1.8.-.-) (Flavin-monooxygenase glucosinolate S-oxygenase-like 9)
FAD_binding_3 domain-containing protein<br>FAD/NAD(P)-binding oxidoreductase family protein

At5g11330 (FAD/NAD(P)-binding oxidoreductase family protein)
NADH-cytochrome b5 reductase-like protein (B5R) (EC 1.6.2.2)<br>NADH-cytochrome b5 reductase (EC 1.6.2.2)
Putative flavin-containing monooxygenase 2 (EC 1.14.13.-)
Aldolase-type TIM barrel family protein<br>tRNA-dihydrouridine synthase (EC 1.3.1.-)
Glucose-methanol-choline (GMC) oxidoreductase family protein<br>Glucose-methanol-choline (GMC) oxidoreductase family protein<br>Uncharacterized protein At5g51950<br>AT5g51950/MSG15_3 (Glucose-methanol-choline (GMC) oxidoreductase family protein)
Probable truncated L-gulonolactone oxidase 7, mitochondrial (AtGulLO7) (EC 1.1.3.8)
Flavodoxin-like domain-containing protein<br>Probable NAD(P)H dehydrogenase (quinone) FQR1-like 3 (EC 1.6.5.2)
Uncharacterized protein At5g67220 (Fragment)<br>AT5g67220/K21H1_18 (FMN-linked oxidoreductases superfamily protein) (Gb|AAF51525.1)

Gene names	Length	Cross-reference (CDD	Cross-reference (Pfam)	Cross-reference (PROSITE)
At1g09400 F14J9.6 F14J9_6	265		PF00724;	
At1g09400 F14J9.6	324		PF00724;	
FMO flavin monooxygenase At1g1	382		PF00743;	
At1g12200 T28K15.7	465		PF00743;	
At1g12570 C24_LOCUS1279	539		PF05199;PF00732;	PS00624;
At1g12570 F5O11.31 F5O11_31	572		PF05199;PF00732;	PS00624;
At1g12570	539		PF05199;PF00732;	PS00624;
F7A19.27 At1g14185	503		PF05199;PF00732;	PS00624;
At1g14190 F7A19.28 F7A19_28	522		PF05199;PF00732;	PS00624;
At1g14190 F7A19.28 F7A19_28	501		PF05199;PF00732;	PS00624;
At1g17990	169		PF00724;	
At1g17990 T10F20.3	261		PF00724;	
At1g17990 F2H15.21	269		PF00724;	
At1g18020 T10F20.3	269		PF00724;	
At1g18020	177		PF00724;	
EMB2421 EMB260 EMBRYO DEFE(	709		PF01494;	
At1g55930 F14J16.20 F14J16_20	596		PF00571;PF03471;PF01595;	PS51371;PS51846;
CBSDUFCH2 At1g55930 F14J16.20	653		PF00571;PF03471;PF01595;	PS51371;PS51846;
At1g63340 F9N12.4	448		PF00743;	
At1g63390 F2K11.23	168		PF00743;	
At1g75200	433		PF00258;PF04055;	PS50902;
TYW1 At1g75200 F22H5.13	647		PF00258;PF04055;PF08608;	PS50902;PS51918;
PLPB LOV PROTEIN LOV PROTEIN /	316	cd00130;	PF13426;	PS50113;PS50112;
PLPB LOV PROTEIN LOV PROTEIN /	358	cd00130;	PF13426;	PS50113;PS50112;
TLP1 FBX4 PLP At2g02710 T20F6.1	399	cd00130;	PF13426;	PS50113;PS50112;
At2g13440 T26C18.3 T26C18_3	723		PF13932;	PS01280;
At2g20360 F11A3.9	402		PF13460;	
CTF2B At2g29720 T27A16.18 T27A	427		PF01494;	
CTF2A At2g35660 T20F21.15 T20F	325		PF01494;	
CTF2A At2g35660 T20F21.15 T20F	439		PF01494;	

MTHFR2 At2g44160 F6E13.29	594	cd00537;	PF02219;	
PHR2 At2g47590 T30B22.11	447		PF00875;	PS51645;
At3g06690 F3E22.17 T8E24.15	187		PF01756;	
CBSDUFCH1 At3g13070 MJG19.2	661		PF00571;PF03471;PF01595;	PS51371;PS51846;
HAOX1 At3g14130	319	cd02809;	PF01070;	PS00557;PS51349;
HAOX1 At3g14130	255	cd02809;	PF01070;	PS00557;PS51349;
GLO4 At3g14130 MAG2.2	363	cd02809;	PF01070;	PS00557;PS51349;
HAOX2 At3g14150	255	cd02809;	PF01070;	PS00557;PS51349;
At3g14150 AXX17_At3g14680	95		PF01070;	PS51349;
HAOX2 At3g14150	277	cd02809;	PF01070;	PS00557;PS51349;
GLO3 At3g14150 MAG2.11	363	cd02809;	PF01070;	PS00557;PS51349;
At3g49640	251	cd02801;	PF01207;	PS01136;
At3g49640	264	cd02801;	PF01207;	PS01136;
At3g49640	303	cd02801;	PF01207;	PS01136;
At3g49640	329	cd02801;	PF01207;	PS01136;
At3g49640 At3g49640/T9C5_230	290	cd02801;	PF01207;	PS01136;
T9C5.230 At3g49640	519	cd02801;	PF01207;	PS01136;
At3g56060 C24_LOCUS15867	577		PF05199;PF00732;	PS00623;PS00624;
At3g56060	577		PF05199;PF00732;	PS00623;PS00624;
F18O21_20 At3g56060	557		PF05199;PF00732;	PS00623;PS00624;
At3g63510	386	cd02801;	PF01207;	
At3g63510	261	cd02801;	PF01207;	
At3g63510	419	cd02801;	PF01207;	
At4g27270 M4I22.80 M4I22_80	154		PF03358;	PS50902;
At4g27270 M4I22.80 M4I22_80	194		PF03358;	PS50902;
At4g27270	205		PF03358;	PS50902;
At4g36750 C7A10.610	273		PF03358;	PS50902;
At4g36750	152		PF03358;	PS50902;
At4g38890 F19H22.4	691	cd02801;	PF01207;	PS01136;PS50103;
At5g07800 MXM12.4	460		PF00743;	
At5g11330 AXX17_At5g11060 AN1	408		PF01494;	
At5g11330 F2I11.220 F2I11_220	262		PF01494;	

At5g11330 At5g11330/F2I11_220	408	PF01494;	
CBR2 At5g20080 F28I16.230	328	PF00970;PF00175;	PS51384;
At5g20080	328	PF00970;PF00175;	PS51384;
FMO2 At5g45180 K18C1.6	459	PF00743;	
At5g47970 K16F13.5 K16F13_5	276 cd02801;	PF01207;	PS01136;
At5g47970 K16F13.5 K16F13_5	387 cd02801;	PF01207;	PS01136;
MSG15.3 MSG15_3 At5g51950	584	PF05199;PF00732;	PS00624;
MSG15.3 MSG15_3 At5g51950	551	PF05199;PF00732;	PS00624;
At5g51950	100	PF05199;	
MSG15.3 MSG15_3 At5g51950	586	PF05199;PF00732;	PS00624;
GULLO7 At5g56470 MCD7.24	252	PF04030;	
At5g58800 AXX17_At5g58220	188	PF03358;	PS50902;
At5g58800 MZN1.26	207	PF03358;	PS50902;
At5g67220	127		
At5g67220 K21H1.18 K21H1_18	423 cd02801;	PF01207;	PS01136;

Protein families	Gene name	Motif
	At1g09400	
NADH:flavin oxidoreductase/NADH oxidase family	At1g09400	
FMO family	At1g12200	
FMO family	At1g12200	
	At1g12570	
	At1g12570	
	At1g12570	
	At1g14185	
	At1g14190	
	At1g14190	
	At1g17990	
	At1g17990	
NADH:flavin oxidoreductase/NADH oxidase family	At1g17990	
NADH:flavin oxidoreductase/NADH oxidase family	At1g18020	
	At1g18020	
	At1g24340	
	At1g55930	
	At1g55930	
FMO family	At1g63340	
FMO family	At1g63390	
	At1g75200	
TYW1 family	At1g75200	
	At2g02710	
	At2g02710	
	At2g02710	
	At2g13440	
Complex I NDUFA9 subunit family	At2g20360	
	At2g29720	
	At2g35660	
	At2g35660	

Methylenetetrahydrofolate reductase family	At2g44160
DNA photolyase class-1 family	At2g47590
Acyl-CoA oxidase family	At3g06690
	At3g13070
FMN-dependent alpha-hydroxy acid dehydrogenase family	At3g14130
FMN-dependent alpha-hydroxy acid dehydrogenase family	At3g14130
FMN-dependent alpha-hydroxy acid dehydrogenase family	At3g14130 MOTIF 361..3
FMN-dependent alpha-hydroxy acid dehydrogenase family	At3g14150
FMN-dependent alpha-hydroxy acid dehydrogenase family	At3g14150
FMN-dependent alpha-hydroxy acid dehydrogenase family	At3g14150
FMN-dependent alpha-hydroxy acid dehydrogenase family	At3g14150 MOTIF 361..3
	At3g49640
	At3g49640
Dus family	At3g49640
	At3g49640
	At3g49640
	At3g49640
	At3g56060
GMC oxidoreductase family	At3g56060
GMC oxidoreductase family	At3g56060
Dus family	At3g63510
	At3g63510
	At3g63510
	At4g27270
	At4g27270
WrbA family	At4g27270
WrbA family	At4g36750
	At4g36750
Dus family, Dus3 subfamily	At4g38890
FMO family	At5g07800
	At5g11330
	At5g11330

	At5g11330
Flavoprotein pyridine nucleotide cytochrome reductase family	At5g20080
Flavoprotein pyridine nucleotide cytochrome reductase family	At5g20080
FMO family	At5g45180
	At5g47970
Dus family	At5g47970
	At5g51950
	At5g51950
	At5g51950
	At5g51950
Oxygen-dependent FAD-linked oxidoreductase family	At5g56470
	At5g58800
WrbA family	At5g58800
	At5g67220
	At5g67220

Sequence similarities

SIMILARITY: Belongs to the NADH:flavin oxidoreductase/NADH oxidase family. {ECO:0000305}.

SIMILARITY: Belongs to the FMO family. {ECO:0000256|RuleBase:RU361177, ECO:0000256|SAAS:SAAS00859851}.
SIMILARITY: Belongs to the FMO family. {ECO:0000305}.

SIMILARITY: Belongs to the NADH:flavin oxidoreductase/NADH oxidase family. {ECO:0000305}.

SIMILARITY: Belongs to the NADH:flavin oxidoreductase/NADH oxidase family. {ECO:0000305}.

SIMILARITY: Belongs to the FMO family. {ECO:0000305}.

SIMILARITY: Belongs to the FMO family. {ECO:0000305}.

SIMILARITY: Belongs to the TYW1 family. {ECO:0000305}.

SIMILARITY: Belongs to the complex I NDUFA9 subunit family. {ECO:0000305}.

SIMILARITY: Belongs to the methylenetetrahydrofolate reductase family. {ECO:0000305}.
SIMILARITY: Belongs to the DNA photolyase class-1 family. {ECO:0000305}.
SIMILARITY: Belongs to the acyl-CoA oxidase family. {ECO:0000305}.
SIMILARITY: Belongs to the FMN-dependent alpha-hydroxy acid dehydrogenase family. {ECO:0000256|PROSITE-ProRule:PRU00683}.<br>SIMILARITY: Belongs to the FMN-dependent alpha-hydroxy acid dehydrogenase family. {ECO:0000256|PROSITE-ProRule:PRU00683}.<br>SIMILARITY: Belongs to the FMN-dependent alpha-hydroxy acid dehydrogenase family. {ECO:0000255|PROSITE-ProRule:PRU00683}.
SIMILARITY: Belongs to the FMN-dependent alpha-hydroxy acid dehydrogenase family. {ECO:0000256|PROSITE-ProRule:PRU00683}.<br>SIMILARITY: Belongs to the FMN-dependent alpha-hydroxy acid dehydrogenase family. {ECO:0000256|PROSITE-ProRule:PRU00683}.<br>SIMILARITY: Belongs to the FMN-dependent alpha-hydroxy acid dehydrogenase family. {ECO:0000256|PROSITE-ProRule:PRU00683}.<br>SIMILARITY: Belongs to the FMN-dependent alpha-hydroxy acid dehydrogenase family. {ECO:0000255|PROSITE-ProRule:PRU00683}.
SIMILARITY: Belongs to the dus family. {ECO:0000256|PIRNR:PIRNR006621}.
SIMILARITY: Belongs to the GMC oxidoreductase family. {ECO:0000256|RuleBase:RU003968}.<br>SIMILARITY: Belongs to the GMC oxidoreductase family. {ECO:0000256|RuleBase:RU003968}.
SIMILARITY: Belongs to the dus family. {ECO:0000256|PIRNR:PIRNR006621}.
SIMILARITY: Belongs to the WrbA family. {ECO:0000305}.
SIMILARITY: Belongs to the WrbA family. {ECO:0000305}.
SIMILARITY: Belongs to the Dus family. Dus3 subfamily. {ECO:0000305}.
SIMILARITY: Belongs to the FMO family. {ECO:0000305}.

SIMILARITY: Belongs to the flavoprotein pyridine nucleotide cytochrome reductase family. {ECO:0000250|UniProtKB:P36060}.
SIMILARITY: Belongs to the flavoprotein pyridine nucleotide cytochrome reductase family. {ECO:0000256|RuleBase:RU361226}.

SIMILARITY: Belongs to the FMO family. {ECO:0000305}.

SIMILARITY: Belongs to the dus family. {ECO:0000256|PIRNR:PIRNR006621}.

SIMILARITY: Belongs to the oxygen-dependent FAD-linked oxidoreductase family. {ECO:0000305}.

SIMILARITY: Belongs to the WrbA family. {ECO:0000305}.

Cross-reference (TIGRFAMs)
TIGR00229;<br>TIGR00229;<br>TIGR00229;
TIGR00136;

TIGR00677;
TIGR01755;<br>TIGR01755;<br>TIGR01755;
TIGR01755;<br>TIGR01755;

TIGR01677;
TIGR01755;<br>TIGR01755;

Chain
CHAIN 1..324; /note="Putative 12-oxophytodienoate reductase-like protein 1"; /id="PRO_0000194486"
CHAIN 1..465; /note="Flavin-containing monooxygenase FMO GS-OX-like 2"; /id="PRO_0000401957"
CHAIN 26..572; /evidence="ECO:0000256|SAM:SignalP"; /id="PRO_5014310281"
CHAIN 20..503; /evidence="ECO:0000256|SAM:SignalP"; /id="PRO_5014313391"
CHAIN 20..522; /evidence="ECO:0000256|SAM:SignalP"; /id="PRO_5010239008"
CHAIN 1..269; /note="Putative 12-oxophytodienoate reductase-like protein 2A"; /id="PRO_0000194487"
CHAIN 1..269; /note="Putative 12-oxophytodienoate reductase-like protein 2B"; /id="PRO_0000416556"
CHAIN 73..653; /note="DUF21 domain-containing protein At1g55930, chloroplastic"; /id="PRO_0000411686"
CHAIN 1..448; /note="Putative flavin-containing monooxygenase FMO GS-OX-like 10"; /id="PRO_0000401965"
CHAIN 1..168; /note="Putative flavin-containing monooxygenase FMO GS-OX-like 11"; /id="PRO_0000401966"
CHAIN 1..647; /note="S-adenosyl-L-methionine-dependent tRNA 4-demethylwyosine synthase"; /id="PRO_0000281832"
CHAIN 1..399; /note="Protein TWIN LOV 1"; /id="PRO_0000272267"
CHAIN 44..402; /note="NADH dehydrogenase [ubiquinone] 1 alpha subcomplex subunit 9, mitochondrial"; /id="PRO_0000410934"

CHAIN 1..594; /note="Methylenetetrahydrofolate reductase 2"; /id="PRO_0000190250"
CHAIN 1..447; /note="Blue-light photoreceptor PHR2"; /id="PRO_0000253763"
CHAIN 1..187; /note="Putative acyl-coenzyme A oxidase At3g06690"; /id="PRO_0000411108"
CHAIN 72..661; /note="Putative DUF21 domain-containing protein At3g13070, chloroplastic"; /id="PRO_0000411685"

CHAIN 1..363; /note="Peroxisomal (S)-2-hydroxy-acid oxidase GLO4"; /id="PRO_0000403407"

CHAIN 1..363; /note="Peroxisomal (S)-2-hydroxy-acid oxidase GLO3"; /id="PRO_0000403406"

CHAIN 26..577; /evidence="ECO:0000256|SAM:SignalP"; /id="PRO_5015099560"
CHAIN 26..557; /evidence="ECO:0000256|SAM:SignalP"; /id="PRO_5004333582"

CHAIN 1..205; /note="Probable NAD(P)H dehydrogenase (quinone) FQR1-like 1"; /id="PRO_0000431284"
CHAIN 1..273; /note="Probable NAD(P)H dehydrogenase (quinone) FQR1-like 2"; /id="PRO_0000431285"

CHAIN 2..691; /note="tRNA-dihydrouridine(47) synthase [NAD(P)(+)]-like"; /id="PRO_0000371960"
CHAIN 1..460; /note="Flavin-containing monooxygenase FMO GS-OX-like 9"; /id="PRO_0000401964"

CHAIN 1..328; /note="NADH-cytochrome b5 reductase-like protein"; /id="PRO_0000167626"
CHAIN 1..459; /note="Putative flavin-containing monooxygenase 2"; /id="PRO_0000249422"
CHAIN 24..584; /evidence="ECO:0000256|SAM:SignalP"; /id="PRO_5010297158"
CHAIN 24..586; /evidence="ECO:0000256|SAM:SignalP"; /id="PRO_5014312554"
CHAIN 103..252; /note="Probable truncated L-gulonolactone oxidase 7, mitochondrial"; /evidence="ECO:0000255"; /id="PRO_0000432508"
CHAIN 1..207; /note="Probable NAD(P)H dehydrogenase (quinone) FQR1-like 3"; /id="PRO_0000431286"

Cross-reference (InterPro)	Cross-reference (SUPFAM)
IPR013785;IPR001155;	
IPR013785;IPR001155;	
IPR036188;IPR000960;IPR020946;	SSF51905;
IPR036188;IPR000960;IPR020946;	SSF51905;
IPR036188;IPR012132;IPR000172;IPR007867;	SSF51905;
IPR036188;IPR012132;IPR000172;IPR007867;	SSF51905;
IPR036188;IPR012132;IPR000172;IPR007867;	SSF51905;
IPR036188;IPR012132;IPR000172;IPR007867;	SSF51905;
IPR036188;IPR012132;IPR000172;IPR007867;	SSF51905;
IPR036188;IPR012132;IPR000172;IPR007867;	SSF51905;
IPR013785;IPR001155;	
IPR013785;IPR001155;	
IPR013785;IPR001155;	
IPR013785;IPR001155;	
IPR013785;IPR001155;	
IPR002938;IPR036188;	SSF51905;
IPR000644;IPR002550;IPR036318;IPR016169;IPR005170;	SSF56176;
IPR000644;IPR002550;IPR036318;IPR016169;IPR005170;	SSF56176;
IPR036188;IPR000960;IPR020946;	SSF51905;
IPR036188;IPR020946;	SSF51905;
IPR013785;IPR001094;IPR008254;IPR029039;IPR007197;	SSF52218;
IPR013785;IPR001094;IPR008254;IPR029039;IPR007197;IPR013917;IPR034556;	SSF52218;
IPR001610;IPR000014;IPR000700;IPR035965;	SSF55785;
IPR001610;IPR000014;IPR000700;IPR035965;	SSF55785;
IPR001610;IPR000014;IPR000700;IPR035965;	SSF55785;
IPR036188;IPR026904;IPR004416;IPR002218;IPR020595;	SSF51905;
IPR016040;IPR036291;	SSF51735;
IPR002938;IPR036188;	SSF51905;
IPR002938;IPR036188;	SSF51905;
IPR002938;IPR036188;	SSF51905;

IPR029041;IPR004621;IPR003171;	SSF51730;
IPR036134;IPR036155;IPR006050;IPR014729;	SSF48173;SSF52425;
IPR002655;IPR036250;	SSF47203;
IPR000644;IPR002550;IPR036318;IPR016169;IPR005170;	SSF56176;
IPR013785;IPR012133;IPR000262;IPR037396;IPR008259;	
IPR013785;IPR012133;IPR000262;IPR037396;IPR008259;	
IPR013785;IPR012133;IPR000262;IPR037396;IPR008259;	
IPR013785;IPR012133;IPR000262;IPR037396;IPR008259;	
IPR013785;IPR000262;IPR037396;	
IPR013785;IPR012133;IPR000262;IPR037396;IPR008259;	
IPR013785;IPR012133;IPR000262;IPR037396;IPR008259;	
IPR013785;IPR035587;IPR001269;IPR018517;	
IPR013785;IPR035587;IPR001269;IPR018517;	
IPR013785;IPR035587;IPR001269;IPR018517;	
IPR013785;IPR035587;IPR001269;IPR018517;	
IPR013785;IPR035587;IPR001269;IPR018517;	
IPR013785;IPR035587;IPR001269;IPR018517;	
IPR036188;IPR012132;IPR000172;IPR007867;	SSF51905;
IPR036188;IPR012132;IPR000172;IPR007867;	SSF51905;
IPR036188;IPR012132;IPR000172;IPR007867;	SSF51905;
IPR013785;IPR035587;IPR004653;IPR001269;	
IPR013785;IPR035587;IPR004653;IPR001269;	
IPR013785;IPR035587;IPR004653;IPR001269;	
IPR008254;IPR029039;IPR010089;IPR005025;	SSF52218;
IPR008254;IPR029039;IPR010089;IPR005025;	SSF52218;
IPR008254;IPR029039;IPR010089;IPR005025;	SSF52218;
IPR008254;IPR029039;IPR010089;IPR005025;	SSF52218;
IPR008254;IPR029039;IPR010089;IPR005025;	SSF52218;
IPR013785;IPR035587;IPR001269;IPR018517;IPR000571;	
IPR036188;IPR000960;IPR020946;	SSF51905;
IPR002938;IPR036188;	SSF51905;
IPR002938;IPR036188;	SSF51905;

IPR002938;IPR036188;	SSF51905;
IPR001834;IPR008333;IPR017927;IPR001709;IPR039261;IPR001433;IPR017938;	SSF52343;SSF63380;
IPR001834;IPR008333;IPR017927;IPR001709;IPR039261;IPR001433;IPR017938;	SSF52343;SSF63380;
CHAIN 1..459; /note="Putative flavin-containing monooxygenase 2"; /id="PRO_0000	SSF51905;
IPR013785;IPR035587;IPR004653;IPR001269;IPR018517;	
IPR013785;IPR035587;IPR004653;IPR001269;IPR018517;	
IPR036188;IPR012132;IPR000172;IPR007867;	SSF51905;
IPR036188;IPR012132;IPR000172;IPR007867;	SSF51905;
IPR036188;IPR007867;	SSF51905;
IPR036188;IPR012132;IPR000172;IPR007867;	SSF51905;
IPR007173;IPR010030;	
IPR008254;IPR029039;IPR010089;IPR005025;	SSF52218;
IPR008254;IPR029039;IPR010089;IPR005025;	SSF52218;
IPR013785;IPR035587;IPR001269;IPR018517;	

Gene ontology (biological process)
defense response to fungus [GO:0050832]
oxidation-reduction process [GO:0055114]<br>oxidation-reduction process [GO:0055114]; tRNA processing [GO:0008033]
protein-chromophore linkage [GO:0018298]; response to stimulus [GO:0050896]
mitochondrial tRNA wobble uridine modification [GO:0070899]; tRNA methylation [GO:0030488]; tRNA w
ubiquinone-6 biosynthetic process [GO:1901006]

methionine biosynthetic process [GO:0009086]; one-carbon metabolic process [GO:0006730]; tetrahydro
protein-chromophore linkage [GO:0018298]; response to stimulus [GO:0050896]
fatty acid beta-oxidation using acyl-CoA oxidase [GO:0033540]; lipid homeostasis [GO:0055088]
defense response to bacterium [GO:0042742]; hydrogen peroxide biosynthetic process [GO:0050665]; lac
defense response to bacterium [GO:0042742]; hydrogen peroxide biosynthetic process [GO:0050665]; inn
oxidation-reduction process [GO:0055114]
oxidation-reduction process [GO:0055114]

L-ascorbic acid biosynthetic process [GO:0019853]
oxidation-reduction process [GO:0055114]

Gene ontology (cellular component)
integral component of membrane [GO:0016021]
nucleus [GO:0005634]
nucleus [GO:0005634]
integral component of membrane [GO:0016021]<br>chloroplast envelope [GO:0009941]; chloroplast membrane [GO:0031969]; integral compon
mitochondrion [GO:0005739]
nucleus [GO:0005634]
mitochondrion [GO:0005739]
mitochondrial matrix [GO:0005759]; mitochondrial respiratory chain complex I [GO:0005747

cytosol [GO:0005829]
cytosol [GO:0005829]; peroxisome [GO:0005777]
chloroplast envelope [GO:0009941]; chloroplast membrane [GO:0031969]; cytosol [GO:0005
peroxisome [GO:0005777]
peroxisome [GO:0005777]
cytosol [GO:0005829]; plasma membrane [GO:0005886]; plasmodesma [GO:0009506]
plasma membrane [GO:0005886]
vacuole [GO:0005773]
plasma membrane [GO:0005886]

plasma membrane [GO:0005886]
Golgi apparatus [GO:0005794]; mitochondrial intermembrane space [GO:0005758]; mitocho
cytoplasm [GO:0005737]; cytosol [GO:0005829]; nucleus [GO:0005634]; phragmoplast [GO:
membrane [GO:0016020]; mitochondrion [GO:0005739]
plasma membrane [GO:0005886]; plastid [GO:0009536]

Gene ontology (GO)
FMN binding [GO:0010181]; oxidoreductase activity [GO:0016491]
FMN binding [GO:0010181]; oxidoreductase activity [GO:0016491]
integral component of membrane [GO:0016021]; flavin adenine dinucleotide binding [GO:0050660]; N,N-dimethylaniline monooxygenase activity [GO:0004499]; NADP binding [GC
flavin adenine dinucleotide binding [GO:0050660]; N,N-dimethylaniline monooxygenase activity [GO:0004499]; NADP binding [GO:0050661]; defense response to fungus [GO:0050
flavin adenine dinucleotide binding [GO:0050660]; oxidoreductase activity, acting on CH-OH group of donors [GO:0016614]
flavin adenine dinucleotide binding [GO:0050660]; oxidoreductase activity, acting on CH-OH group of donors [GO:0016614]
flavin adenine dinucleotide binding [GO:0050660]; oxidoreductase activity, acting on CH-OH group of donors [GO:0016614]
flavin adenine dinucleotide binding [GO:0050660]; oxidoreductase activity, acting on CH-OH group of donors [GO:0016614]
flavin adenine dinucleotide binding [GO:0050660]; oxidoreductase activity, acting on CH-OH group of donors [GO:0016614]
flavin adenine dinucleotide binding [GO:0050660]; oxidoreductase activity, acting on CH-OH group of donors [GO:0016614]
FMN binding [GO:0010181]; oxidoreductase activity [GO:0016491]
FMN binding [GO:0010181]; oxidoreductase activity [GO:0016491]
nucleus [GO:0005634]; FMN binding [GO:0010181]; oxidoreductase activity [GO:0016491]
nucleus [GO:0005634]; FMN binding [GO:0010181]; oxidoreductase activity [GO:0016491]
FMN binding [GO:0010181]; oxidoreductase activity [GO:0016491]
FAD binding [GO:0071949]
integral component of membrane [GO:0016021]; flavin adenine dinucleotide binding [GO:0050660]
chloroplast envelope [GO:0009941]; chloroplast membrane [GO:0031969]; integral component of membrane [GO:0016021]; nucleus [GO:0005634]; plastid [GO:0009536]; flavin a
flavin adenine dinucleotide binding [GO:0050660]; N,N-dimethylaniline monooxygenase activity [GO:0004499]; NADP binding [GO:0050661]
flavin adenine dinucleotide binding [GO:0050660]; N,N-dimethylaniline monooxygenase activity [GO:0004499]; NADP binding [GO:0050661]
catalytic activity [GO:0003824]; FMN binding [GO:0010181]; iron-sulfur cluster binding [GO:0051536]; oxidation-reduction process [GO:0055114]
mitochondrion [GO:0005739]; 4 iron, 4 sulfur cluster binding [GO:0051539]; FMN binding [GO:0010181]; metal ion binding [GO:0046872]; tRNA-4-demethylwyosine synthase activ
nucleus [GO:0005634]; photoreceptor activity [GO:0009881]; protein-chromophore linkage [GO:0018298]; response to stimulus [GO:0050896]
mitochondrion [GO:0005739]; flavin adenine dinucleotide binding [GO:0050660]; mitochondrial tRNA wobble uridine modification [GO:0070899]; tRNA methylation [GO:0030488]
mitochondrial matrix [GO:0005759]; mitochondrial respiratory chain complex I [GO:0005747]; mitochondrion [GO:0005739]; NADH dehydrogenase activity [GO:0003954]; ubiquin
FAD binding [GO:0071949]; monooxygenase activity [GO:0004497]
FAD binding [GO:0071949]
FAD binding [GO:0071949]; monooxygenase activity [GO:0004497]

cytosol [GO:0005829]; FAD binding [GO:0071949]; methylenetetrahydrofolate reductase (NAD(P)H) activity [GO:0004489]; methionine biosynthetic process [GO:0009086]; one-ca
photoreceptor activity [GO:0009881]; protein-chromophore linkage [GO:0018298]; response to stimulus [GO:0050896]
cytosol [GO:0005829]; peroxisome [GO:0005777]; acyl-CoA oxidase activity [GO:0003997]; fatty acid binding [GO:0005504]; flavin adenine dinucleotide binding [GO:0050660]; fatt
chloroplast envelope [GO:0009941]; chloroplast membrane [GO:0031969]; cytosol [GO:0005829]; integral component of membrane [GO:0016021]; flavin adenine dinucleotide bin
FMN binding [GO:0010181]; oxidoreductase activity [GO:0016491]
FMN binding [GO:0010181]; oxidoreductase activity [GO:0016491]
peroxisome [GO:0005777]; FMN binding [GO:0010181]; L-lactate dehydrogenase activity [GO:0004459]; long-chain-(S)-2-hydroxy-long-chain-acid oxidase activity [GO:0052853]; m
FMN binding [GO:0010181]; oxidoreductase activity [GO:0016491]
oxidoreductase activity [GO:0016491]
FMN binding [GO:0010181]; oxidoreductase activity [GO:0016491]
peroxisome [GO:0005777]; FMN binding [GO:0010181]; L-lactate dehydrogenase activity [GO:0004459]; long-chain-(S)-2-hydroxy-long-chain-acid oxidase activity [GO:0052853]; m
flavin adenine dinucleotide binding [GO:0050660]; tRNA dihydrouridine synthase activity [GO:0017150]
flavin adenine dinucleotide binding [GO:0050660]; tRNA dihydrouridine synthase activity [GO:0017150]
flavin adenine dinucleotide binding [GO:0050660]; tRNA dihydrouridine synthase activity [GO:0017150]
flavin adenine dinucleotide binding [GO:0050660]; tRNA dihydrouridine synthase activity [GO:0017150]
flavin adenine dinucleotide binding [GO:0050660]; tRNA dihydrouridine synthase activity [GO:0017150]
flavin adenine dinucleotide binding [GO:0050660]; tRNA dihydrouridine synthase activity [GO:0017150]
flavin adenine dinucleotide binding [GO:0050660]; oxidoreductase activity, acting on CH-OH group of donors [GO:0016614]
flavin adenine dinucleotide binding [GO:0050660]; oxidoreductase activity, acting on CH-OH group of donors [GO:0016614]
flavin adenine dinucleotide binding [GO:0050660]; oxidoreductase activity, acting on CH-OH group of donors [GO:0016614]
flavin adenine dinucleotide binding [GO:0050660]; tRNA dihydrouridine synthase activity [GO:0017150]
flavin adenine dinucleotide binding [GO:0050660]; tRNA dihydrouridine synthase activity [GO:0017150]
flavin adenine dinucleotide binding [GO:0050660]; tRNA dihydrouridine synthase activity [GO:0017150]
FMN binding [GO:0010181]; NAD(P)H dehydrogenase (quinone) activity [GO:0003955]
FMN binding [GO:0010181]; NAD(P)H dehydrogenase (quinone) activity [GO:0003955]
cytosol [GO:0005829]; plasma membrane [GO:0005886]; plasmodesma [GO:0009506]; FMN binding [GO:0010181]; NAD(P)H dehydrogenase (quinone) activity [GO:0003955]; oxid
plasma membrane [GO:0005886]; FMN binding [GO:0010181]; NAD(P)H dehydrogenase (quinone) activity [GO:0003955]; oxidation-reduction process [GO:0055114]
FMN binding [GO:0010181]; NAD(P)H dehydrogenase (quinone) activity [GO:0003955]
vacuole [GO:0005773]; flavin adenine dinucleotide binding [GO:0050660]; metal ion binding [GO:0046872]; tRNA dihydrouridine synthase activity [GO:0017150]
flavin adenine dinucleotide binding [GO:0050660]; N,N-dimethylaniline monooxygenase activity [GO:0004499]; NADP binding [GO:0050661]
plasma membrane [GO:0005886]; FAD binding [GO:0071949]
FAD binding [GO:0071949]

plasma membrane [GO:0005886]; FAD binding [GO:0071949]
Golgi apparatus [GO:0005794]; mitochondrial intermembrane space [GO:0005758]; mitochondrion [GO:0005739]; plant-type cell wall [GO:0009505]; plastid [GO:0009536]; copper<br>cytochrome-b5 reductase activity, acting on NAD(P)H [GO:0004128]
flavin adenine dinucleotide binding [GO:0050660]; N,N-dimethylaniline monooxygenase activity [GO:0004499]; NADP binding [GO:0050661]
flavin adenine dinucleotide binding [GO:0050660]; tRNA dihydrouridine synthase activity [GO:0017150]
cytoplasm [GO:0005737]; cytosol [GO:0005829]; nucleus [GO:0005634]; phragmoplast [GO:0009524]; flavin adenine dinucleotide binding [GO:0050660]; tRNA dihydrouridine synt
flavin adenine dinucleotide binding [GO:0050660]; oxidoreductase activity, acting on CH-OH group of donors [GO:0016614]
flavin adenine dinucleotide binding [GO:0050660]; oxidoreductase activity, acting on CH-OH group of donors [GO:0016614]
oxidoreductase activity, acting on CH-OH group of donors [GO:0016614]
flavin adenine dinucleotide binding [GO:0050660]; oxidoreductase activity, acting on CH-OH group of donors [GO:0016614]
membrane [GO:0016020]; mitochondrion [GO:0005739]; D-arabinono-1,4-lactone oxidase activity [GO:0003885]; L-gulonolactone oxidase activity [GO:0050105]; L-ascorbic acid bi
FMN binding [GO:0010181]; NAD(P)H dehydrogenase (quinone) activity [GO:0003955]
plasma membrane [GO:0005886]; plastid [GO:0009536]; FMN binding [GO:0010181]; NAD(P)H dehydrogenase (quinone) activity [GO:0003955]; oxidation-reduction process [GO:0
flavin adenine dinucleotide binding [GO:0050660]; tRNA dihydrouridine synthase activity [GO:0017150]

Gene ontol	Gene ontol	Cofactor	Gene name	Gene names (synonym)
FMN bindir	GO:0010181; GO:0016491			
FMN bindir	GO:001018	COFACTOR: Name=FMN; Xref=ChEBI:CHEBI:58210; Evidence={ECO:0000250};		
flavin aden	GO:000449	COFACTOR	FMO	flavin monooxygenase
flavin aden	GO:000449	COFACTOR: Name=FAD; Xref=ChEBI:CHEBI:57692; Evidence={ECO:0000250};		
flavin aden	GO:0016614; GO:0050660			
flavin aden	GO:001661	COFACTOR: Name=FAD; Xref=ChEBI:CHEBI:57692; Evidence={ECO:0000256|PIRSR:PIRSR000137-2};		
flavin aden	GO:001661	COFACTOR: Name=FAD; Xref=ChEBI:CHEBI:57692; Evidence={ECO:0000256|PIRSR:PIRSR000137-2};		
flavin aden	GO:001661	COFACTOR	F7A19.27	
flavin aden	GO:001661	COFACTOR: Name=FAD; Xref=ChEBI:CHEBI:57692; Evidence={ECO:0000256|PIRSR:PIRSR000137-2};		
flavin aden	GO:001661	COFACTOR: Name=FAD; Xref=ChEBI:CHEBI:57692; Evidence={ECO:0000256|PIRSR:PIRSR000137-2};		
FMN bindir	GO:0010181; GO:0016491			
FMN bindir	GO:0010181; GO:0016491			
FMN bindir	GO:000563	COFACTOR: Name=FMN; Xref=ChEBI:CHEBI:58210; Evidence={ECO:0000250};		
FMN bindir	GO:000563	COFACTOR: Name=FMN; Xref=ChEBI:CHEBI:58210; Evidence={ECO:0000250};		
FMN bindir	GO:0010181; GO:0016491			
FAD bindin	GO:0071949		EMB2421	EMB260 EMBRYO DEFECTIVE 2421 EMBRYO DEFECTIVE 260
flavin aden	GO:0016021; GO:0050660			
flavin aden	GO:0005634; GO:0009		CBSDUFCH2	
flavin aden	GO:000449	COFACTOR: Name=FAD; Xref=ChEBI:CHEBI:57692; Evidence={ECO:0000250};		
flavin aden	GO:000449	COFACTOR: Name=FAD; Xref=ChEBI:CHEBI:57692; Evidence={ECO:0000250};		
catalytic ac	GO:0003824; GO:0010181; GO:0051536; GO:0055114			
4 iron, 4 su	GO:000573	COFACTOR	TYW1	
			PLPB	LOV PROTEIN LOV PROTEIN A LOV protein B LOV PROTEIN C PAS PLP PLPA PLPC
			PLPB	LOV PROTEIN LOV PROTEIN A LOV protein B LOV PROTEIN C PAS PLP PLPA PLPC
photorece	GO:0005634; GO:0009		TLP1	FBX4 PLP
flavin aden	GO:0002098; GO:0005739; GO:0030488; GO:0050660; GO:0070899			
NADH deh	GO:000395	COFACTOR: Name=FAD; Xref=ChEBI:CHEBI:57692; Evidence={ECO:0000250}; Note=Binds 1 FAD per subunit. {ECO:0000250};		
FAD bindin	GO:0004497; GO:0071		CTF2B	
FAD bindin	GO:0071949		CTF2A	
FAD bindin	GO:0004497; GO:0071		CTF2A	

FAD bindin	GO:000448	COFACTOR MTHFR2
photorecep	GO:000988	COFACTOR PHR2
acyl-CoA o	GO:0003997; GO:0005504; GO:0005777; GO:0005829; GO:0033540; GO:0050660; GO:0055088	
flavin aden	GO:0005829; GO:0009	CBSDUFCH1
FMN bindin	GO:001018	COFACTOR HAOX1
FMN bindin	GO:001018	COFACTOR HAOX1
FMN bindin	GO:000445	COFACTOR GLO4
FMN bindin	GO:001018	COFACTOR HAOX2
oxidoreduc	GO:001649	COFACTOR: Name=FMN; Xref=ChEBI:CHEBI:58210; Evidence={ECO:0000256|PROSITE-ProRule:PRU00683};
FMN bindin	GO:001018	COFACTOR HAOX2
FMN bindin	GO:000275	COFACTOR GLO3
flavin aden	GO:0017150; GO:0050660	
flavin aden	GO:0017150; GO:0050660	
flavin aden	GO:001715	COFACTOR: Name=FMN; Xref=ChEBI:CHEBI:58210; Evidence={ECO:0000256|PIRNR:PIRNR006621};
flavin aden	GO:0017150; GO:0050660	
flavin aden	GO:0017150; GO:0050660	
flavin aden	GO:0017150; GO:0050	T9C5.230
flavin aden	GO:0016614; GO:0050660	
flavin aden	GO:001661	COFACTOR: Name=FAD; Xref=ChEBI:CHEBI:57692; Evidence={ECO:0000256|PIRSR:PIRSR000137-2};
flavin aden	GO:001661	COFACTOR F18O21_20
flavin aden	GO:001715	COFACTOR: Name=FMN; Xref=ChEBI:CHEBI:58210; Evidence={ECO:0000256|PIRNR:PIRNR006621};
flavin aden	GO:0017150; GO:0050660	
flavin aden	GO:0017150; GO:0050660	
FMN bindin	GO:0003955; GO:0010181	
FMN bindin	GO:0003955; GO:0010181	
FMN bindin	GO:000395	COFACTOR: Name=FMN; Xref=ChEBI:CHEBI:58210; Evidence={ECO:0000250|UniProtKB:Q9LSQ5}; Note=Binds 1 FMN per monomer. {ECO:0000250|U
FMN bindin	GO:000395	COFACTOR: Name=FMN; Xref=ChEBI:CHEBI:58210; Evidence={ECO:0000250|UniProtKB:Q9LSQ5}; Note=Binds 1 FMN per monomer. {ECO:0000250|U
FMN bindin	GO:0003955; GO:0010181	
flavin aden	GO:000577	COFACTOR: Name=FMN; Xref=ChEBI:CHEBI:58210; Evidence={ECO:0000250|UniProtKB:Q5SMC7};
flavin aden	GO:000449	COFACTOR: Name=FAD; Xref=ChEBI:CHEBI:57692; Evidence={ECO:0000250};
FAD bindin	GO:0005886; GO:0071949	
FAD bindin	GO:0071949	

FAD bindin	GO:0005886; GO:0071949			
copper ion	GO:000412	COFACTOR	CBR2	
cytochrom	GO:000412	COFACTOR: Name=FAD; Xref=ChEBI:CHEBI:57692; Evidence={ECO:0000256|RuleBase:RU361226, ECO:0000256|SAAS:SAAS01057700};		
flavin aden	GO:000449	COFACTOR	FMO2	
flavin aden	GO:0017150; GO:0050660			
flavin aden	GO:000563	COFACTOR: Name=FMN; Xref=ChEBI:CHEBI:58210; Evidence={ECO:0000256|PIRNR:PIRNR006621};		
flavin aden	GO:001661	COFACTOR	MSG15.3	MSG15_3
flavin aden	GO:001661	COFACTOR	MSG15.3	MSG15_3
oxidoreduc	GO:0016614			
flavin aden	GO:001661	COFACTOR	MSG15.3	MSG15_3
D-arabinon	GO:0003885; GO:0005		GULLO7	
FMN bindir	GO:0003955; GO:0010181			
FMN bindir	GO:000395	COFACTOR: Name=FMN; Xref=ChEBI:CHEBI:58210; Evidence={ECO:0000250|UniProtKB:Q9LSQ5}; Note=Binds 1 FMN per monomer. {ECO:0000250|U		
flavin aden	GO:0017150; GO:0050660			

JniProtKB:Q9LSQ5};
JniProtKB:Q9LSQ5};

JniProtKB:Q9LSQ5};

Table S4 flavin binding prediction of the 38 genes categorized in evidence code 3 with the HMMER biosequence analysis.

seq id	alignment start	alignment end
> AT1G09400.1 | Symbols:no symbol available | no full name available | Chr1:3033676-3034993 REVERSE LENGTH=324	2	323
> AT1G12200.1 | A3:P14:FMO | flavin monooxygenase | Chr1:4137627-4139835 FORWARD LENGTH=465	12	244
> AT1G12200.1 | Symbols:FMO | flavin monooxygenase | Chr1:4137627-4139835 FORWARD LENGTH=465	268	377
> AT1G12200.1 | Symbols:FMO | flavin monooxygenase | Chr1:4137627-4139835 FORWARD LENGTH=465	81	309
> AT1G12200.1 | Symbols:FMO | flavin monooxygenase | Chr1:4137627-4139835 FORWARD LENGTH=465	13	307
> AT1G12200.1 | Symbols:FMO | flavin monooxygenase | Chr1:4137627-4139835 FORWARD LENGTH=465	13	48
> AT1G12200.1 | Symbols:FMO | flavin monooxygenase | Chr1:4137627-4139835 FORWARD LENGTH=465	16	51
> AT1G12200.1 | Symbols:FMO | flavin monooxygenase | Chr1:4137627-4139835 FORWARD LENGTH=465	14	49
> AT1G12200.1 | Symbols:FMO | flavin monooxygenase | Chr1:4137627-4139835 FORWARD LENGTH=465	13	47
> AT1G12200.1 | Symbols:FMO | flavin monooxygenase | Chr1:4137627-4139835 FORWARD LENGTH=465	108	244
> AT1G12200.1 | Symbols:FMO | flavin monooxygenase | Chr1:4137627-4139835 FORWARD LENGTH=465	22	55
> AT1G12200.1 | Symbols:FMO | flavin monooxygenase | Chr1:4137627-4139835 FORWARD LENGTH=465	15	177
> AT1G12200.1 | Symbols:FMO | flavin monooxygenase | Chr1:4137627-4139835 FORWARD LENGTH=465	13	44
> AT1G12570.1 | Symbols:no symbol available | no full name available | Chr1:4278192-4280753 REVERSE LENGTH=572	46	319
> AT1G12570.1 | Symbols:no symbol available | no full name available | Chr1:4278192-4280753 REVERSE LENGTH=572	409	558
> AT1G14185.1 | Symbols:no symbol available | no full name available | Chr1:4850284-4851885 FORWARD LENGTH=503	37	140
> AT1G14185.1 | Symbols:no symbol available | no full name available | Chr1:4850284-4851885 FORWARD LENGTH=503	217	310
> AT1G14185.1 | Symbols:no symbol available | no full name available | Chr1:4850284-4851885 FORWARD LENGTH=503	364	492
> AT1G14190.1 | Symbols:no symbol available | no full name available | Chr1:4852802-4854307 FORWARD LENGTH=501	16	113
> AT1G14190.1 | Symbols:no symbol available | no full name available | Chr1:4852802-4854307 FORWARD LENGTH=501	196	290
> AT1G14190.1 | Symbols:no symbol available | no full name available | Chr1:4852802-4854307 FORWARD LENGTH=501	344	472
> AT1G17990.1 | Symbols:no symbol available | no full name available | Chr1:6192455-6193755 REVERSE LENGTH=269	9	257
> AT1G18020.1 | Symbols:no symbol available | no full name available | Chr1:6202272-6203572 FORWARD LENGTH=269	9	257
> AT1G24340.1 | Symbols:EMB260,EMB2421 | EMBRYO DEFECTIVE 260,EMBRYO DEFECTIVE 2421 | Chr1:8635416-8638866 FORWARD LENGTH=709	47	421
> AT1G55930.1 | Symbols:no symbol available | no full name available | Chr1:20918895-20922133 FORWARD LENGTH=653	158	335
> AT1G55930.1 | Symbols:no symbol available | no full name available | Chr1:20918895-20922133 FORWARD LENGTH=653	495	599

> AT1G55930.1 | Symbols:no symbol available | no full name available | Chr1:20918895-20922133 FORWARD LENGTH=653	350	408
> AT1G55930.1 | Symbols:no symbol available | no full name available | Chr1:20918895-20922133 FORWARD LENGTH=653	421	473
> AT1G63340.1 | Symbols:no symbol available | no full name available | Chr1:23491882-23494019 REVERSE LENGTH=424	13	237
> AT1G63340.1 | Symbols:no symbol available | no full name available | Chr1:23491882-23494019 REVERSE LENGTH=424	254	351
> AT1G63340.1 | Symbols:no symbol available | no full name available | Chr1:23491882-23494019 REVERSE LENGTH=424	86	304
> AT1G63340.1 | Symbols:no symbol available | no full name available | Chr1:23491882-23494019 REVERSE LENGTH=424	13	301
> AT1G63340.1 | Symbols:no symbol available | no full name available | Chr1:23491882-23494019 REVERSE LENGTH=424	16	51
> AT1G63340.1 | Symbols:no symbol available | no full name available | Chr1:23491882-23494019 REVERSE LENGTH=424	14	48
> AT1G63340.1 | Symbols:no symbol available | no full name available | Chr1:23491882-23494019 REVERSE LENGTH=424	13	47
> AT1G63340.1 | Symbols:no symbol available | no full name available | Chr1:23491882-23494019 REVERSE LENGTH=424	15	174
> AT1G63340.1 | Symbols:no symbol available | no full name available | Chr1:23491882-23494019 REVERSE LENGTH=424	22	48
> AT1G63390.1 | Symbols:no symbol available | no full name available | Chr1:23506461-23506967 REVERSE LENGTH=168	12	162
> AT1G63390.1 | Symbols:no symbol available | no full name available | Chr1:23506461-23506967 REVERSE LENGTH=168	15	49
> AT1G63390.1 | Symbols:no symbol available | no full name available | Chr1:23506461-23506967 REVERSE LENGTH=168	13	48
> AT1G63390.1 | Symbols:no symbol available | no full name available | Chr1:23506461-23506967 REVERSE LENGTH=168	12	46
> AT1G63390.1 | Symbols:no symbol available | no full name available | Chr1:23506461-23506967 REVERSE LENGTH=168	12	51
> AT1G63390.1 | Symbols:no symbol available | no full name available | Chr1:23506461-23506967 REVERSE LENGTH=168	12	48
> AT1G63390.1 | Symbols:no symbol available | no full name available | Chr1:23506461-23506967 REVERSE LENGTH=168	21	134
> AT1G63390.1 | Symbols:no symbol available | no full name available | Chr1:23506461-23506967 REVERSE LENGTH=168	12	43
> AT1G63390.1 | Symbols:no symbol available | no full name available | Chr1:23506461-23506967 REVERSE LENGTH=168	12	46
> AT1G75200.1 | Symbols:no symbol available | no full name available | Chr1:28220849-28223597 REVERSE LENGTH=647	52	193
> AT1G75200.1 | Symbols:no symbol available | no full name available | Chr1:28220849-28223597 REVERSE LENGTH=647	511	572
> AT1G75200.1 | Symbols:no symbol available | no full name available | Chr1:28220849-28223597 REVERSE LENGTH=647	327	507
> AT2G02710.1 | Symbols:PLPA,PLPB,PLPC,PLP | PAS/LOV protein B,PAS/LOV PROTEIN C,PAS/LOV PROTEIN A,PAS/LOV PROTEIN | Chr2:758812-760608 REVERSE LENGTH=399	49	141
> AT2G02710.1 | Symbols:PLPA,PLPB,PLPC,PLP | PAS/LOV protein B,PAS/LOV PROTEIN C,PAS/LOV PROTEIN A,PAS/LOV PROTEIN | Chr2:758812-760608 REVERSE LENGTH=399	267	362
> AT2G02710.1 | Symbols:PLPA,PLPB,PLPC,PLP | PAS/LOV protein B,PAS/LOV PROTEIN C,PAS/LOV PROTEIN A,PAS/LOV PROTEIN | Chr2:758812-760608 REVERSE LENGTH=399	51	128
> AT2G02710.1 | Symbols:PLPA,PLPB,PLPC,PLP | PAS/LOV protein B,PAS/LOV PROTEIN C,PAS/LOV PROTEIN A,PAS/LOV PROTEIN | Chr2:758812-760608 REVERSE LENGTH=399	273	360
> AT2G13440.1 | Symbols:no symbol available | no full name available | Chr2:5593270-5598296 REVERSE LENGTH=723	74	466
> AT2G13440.1 | Symbols:no symbol available | no full name available | Chr2:5593270-5598296 REVERSE LENGTH=723	468	683

> AT2G20360.1 | Symbols:no symbol available | no full name available | Chr2:8786070-8789098 FORWARD LENGTH=402	75	271
> AT2G20360.1 | Symbols:no symbol available | no full name available | Chr2:8786070-8789098 FORWARD LENGTH=402	72	283
> AT2G20360.1 | Symbols:no symbol available | no full name available | Chr2:8786070-8789098 FORWARD LENGTH=402	72	299
> AT2G20360.1 | Symbols:no symbol available | no full name available | Chr2:8786070-8789098 FORWARD LENGTH=402	73	146
> AT2G29720.1 | Symbols:CTF2B | | Chr2:12700592-12702289 REVERSE LENGTH=427	44	379
> AT2G29720.1 | Symbols:CTF2B | | Chr2:12700592-12702289 REVERSE LENGTH=427	187	401
> AT2G29720.1 | Symbols:CTF2B | | Chr2:12700592-12702289 REVERSE LENGTH=427	47	93
> AT2G29720.1 | Symbols:CTF2B | | Chr2:12700592-12702289 REVERSE LENGTH=427	45	226
> AT2G35660.1 | Symbols:CTF2A | | Chr2:14988499-14990320 FORWARD LENGTH=439	46	384
> AT2G35660.1 | Symbols:CTF2A | | Chr2:14988499-14990320 FORWARD LENGTH=439	49	92
> AT2G35660.1 | Symbols:CTF2A | | Chr2:14988499-14990320 FORWARD LENGTH=439	47	202
> AT2G35660.1 | Symbols:CTF2A | | Chr2:14988499-14990320 FORWARD LENGTH=439	189	382
> AT2G35660.1 | Symbols:CTF2A | | Chr2:14988499-14990320 FORWARD LENGTH=439	47	80
> AT2G44160.1 | Symbols:MTHFR2 | methylenetetrahydrofolate reductase 2 | Chr2:18262301-18265185 FORWARD LENGTH=594	6	301
> AT2G47590.1 | Symbols:PHR2 | photolyase/blue-light receptor 2 | Chr2:19521888-19523732 FORWARD LENGTH=447	117	275
> AT3G06690.1 | Symbols:no symbol available | no full name available | Chr3:2115589-2116825 REVERSE LENGTH=187	39	171
> AT3G13070.1 | Symbols:no symbol available | no full name available | Chr3:4191511-4195112 REVERSE LENGTH=661	163	340
> AT3G13070.1 | Symbols:no symbol available | no full name available | Chr3:4191511-4195112 REVERSE LENGTH=661	500	604
> AT3G13070.1 | Symbols:no symbol available | no full name available | Chr3:4191511-4195112 REVERSE LENGTH=661	355	413
> AT3G13070.1 | Symbols:no symbol available | no full name available | Chr3:4191511-4195112 REVERSE LENGTH=661	423	478
> AT3G14130.1 | Symbols:HAOX1 | | Chr3:4685844-4687852 REVERSE LENGTH=363	14	353
> AT3G14130.1 | Symbols:HAOX1 | | Chr3:4685844-4687852 REVERSE LENGTH=363	233	340
> AT3G14130.1 | Symbols:HAOX1 | | Chr3:4685844-4687852 REVERSE LENGTH=363	223	307
> AT3G14150.1 | Symbols:HAOX2 | | Chr3:4690667-4692679 REVERSE LENGTH=363	14	353
> AT3G14150.1 | Symbols:HAOX2 | | Chr3:4690667-4692679 REVERSE LENGTH=363	228	340
> AT3G14150.1 | Symbols:HAOX2 | | Chr3:4690667-4692679 REVERSE LENGTH=363	212	307
> AT3G14150.1 | Symbols:HAOX2 | | Chr3:4690667-4692679 REVERSE LENGTH=363	274	309
> AT3G49640.1 | Symbols:no symbol available | no full name available | Chr3:18400480-18402809 REVERSE LENGTH=329	8	255
> AT3G56060.1 | Symbols:no symbol available | no full name available | Chr3:20803328-20805983 REVERSE LENGTH=577	46	322
> AT3G56060.1 | Symbols:no symbol available | no full name available | Chr3:20803328-20805983 REVERSE LENGTH=577	412	557

> AT3G63510.1 | Symbols:no symbol available | no full name available | Chr3:23450542-23452682 FORWARD LENGTH=419	56	365
> AT4G27270.1 | Symbols:no symbol available | no full name available | Chr4:13661458-13663243 REVERSE LENGTH=205	18	144
> AT4G27270.1 | Symbols:no symbol available | no full name available | Chr4:13661458-13663243 REVERSE LENGTH=205	4	120
> AT4G36750.1 | Symbols:no symbol available | no full name available | Chr4:17324642-17326215 FORWARD LENGTH=273	89	215
> AT4G36750.1 | Symbols:no symbol available | no full name available | Chr4:17324642-17326215 FORWARD LENGTH=273	77	208
> AT4G38890.1 | Symbols:no symbol available | no full name available | Chr4:18135909-18139100 REVERSE LENGTH=691	342	609
> AT5G07800.1 | Symbols:no symbol available | no full name available | Chr5:2486717-2489241 REVERSE LENGTH=460	14	247
> AT5G07800.1 | Symbols:no symbol available | no full name available | Chr5:2486717-2489241 REVERSE LENGTH=460	266	410
> AT5G07800.1 | Symbols:no symbol available | no full name available | Chr5:2486717-2489241 REVERSE LENGTH=460	91	318
> AT5G07800.1 | Symbols:no symbol available | no full name available | Chr5:2486717-2489241 REVERSE LENGTH=460	14	314
> AT5G07800.1 | Symbols:no symbol available | no full name available | Chr5:2486717-2489241 REVERSE LENGTH=460	18	53
> AT5G07800.1 | Symbols:no symbol available | no full name available | Chr5:2486717-2489241 REVERSE LENGTH=460	15	49
> AT5G07800.1 | Symbols:no symbol available | no full name available | Chr5:2486717-2489241 REVERSE LENGTH=460	16	53
> AT5G07800.1 | Symbols:no symbol available | no full name available | Chr5:2486717-2489241 REVERSE LENGTH=460	117	244
> AT5G07800.1 | Symbols:no symbol available | no full name available | Chr5:2486717-2489241 REVERSE LENGTH=460	15	46
> AT5G11330.1 | Symbols:no symbol available | no full name available | Chr5:3617342-3618861 REVERSE LENGTH=408	8	367
> AT5G20080.1 | Symbols:no symbol available | no full name available | Chr5:6782708-6786360 FORWARD LENGTH=328	193	299
> AT5G20080.1 | Symbols:no symbol available | no full name available | Chr5:6782708-6786360 FORWARD LENGTH=328	81	183
> AT5G45180.1 | Symbols:no symbol available | no full name available | Chr5:18274179-18276586 REVERSE LENGTH=459	11	248
> AT5G45180.1 | Symbols:no symbol available | no full name available | Chr5:18274179-18276586 REVERSE LENGTH=459	270	459
> AT5G45180.1 | Symbols:no symbol available | no full name available | Chr5:18274179-18276586 REVERSE LENGTH=459	12	247
> AT5G45180.1 | Symbols:no symbol available | no full name available | Chr5:18274179-18276586 REVERSE LENGTH=459	15	69
> AT5G47970.1 | Symbols:no symbol available | no full name available | Chr5:19423420-19425585 FORWARD LENGTH=387	14	317
> AT5G51950.1 | Symbols:no symbol available | no full name available | Chr5:21106093-21108559 REVERSE LENGTH=586	46	320
> AT5G51950.1 | Symbols:no symbol available | no full name available | Chr5:21106093-21108559 REVERSE LENGTH=586	421	566
> AT5G56470.1 | Symbols:GulLO7,AtGulLO7 | L -gulono-1,4-lactone (L -GulL) oxidase 1 | Chr5:22869060-22869958 FORWARD LENGTH=252	63	198
> AT5G58800.1 | Symbols:no symbol available | no full name available | Chr5:23746032-23746895 REVERSE LENGTH=207	5	147
> AT5G67220.1 | Symbols:no symbol available | no full name available | Chr5:26820276-26821926 REVERSE LENGTH=423	91	380

envelope start	envelope end	hmm acc	hmm name	hmm start	hmm end	hmm length	bit score	Individual E-value	Conditional E-value	database signifcan	outcompeted	clan
1	323	PF00724.20	Oxidored_FMN	9	342	342	29203	6,3E-86	3,5E-90	1	0	CL0036
11	247	PF00743.19	FMO-like	2	217	532	13221	1,9E-37	1,3E-40	1	0	CL0063
251	408	PF00743.19	FMO-like	306	416	532	6156	4,9E-16	3,3E-19	1	0	CL0063
74	319	PF13738.6	Pyr_redox_3	49	284	305	5511	6,8E-14	4,5E-17	1	1	CL0063
12	314	PF07992.14	Pyr_redox_2	2	253	294	5391	1,5E-13	1E-16	1	1	CL0063
13	60	PF01266.24	DAO	1	35	352	2821	0,000013	9E-09	1	1	CL0063
16	72	PF13450.6	NAD_binding_8	1	36	68	4134	1,3E-09	8,7E-13	1	1	CL0063
13	55	PF00890.24	FAD_binding_2	2	37	417	2923	0,0000047	3,2E-09	1	1	CL0063
12	50	PF03486.14	HI0933_like	2	36	409	2715	0,000016	0,00000001	1	1	CL0063
104	254	PF13434.6	K_oxygenase	92	227	342	2522	0,00008	5,4E-08	1	1	CL0063
21	181	PF01593.24	Amino_oxidase	2	36	452	2428	0,00017	0,00000012	1	1	CL0063
15	178	PF13454.6	NAD_binding_9	1	155	156	2223	11	0,00000074	1	1	CL0063
11	46	PF01494.19	FAD_binding_3	3	34	349	2272	0,00049	0,00000033	1	1	CL0063
46	320	PF00732.19	**GMC_oxred_N**	1	295	296	10050	1,1E-27	1,2E-31	1	0	CL0063
409	560	PF05199.13	GMC_oxred_C	1	142	144	9505	5,5E-26	6,1E-30	1	0	
37	162	PF00732.19	**GMC_oxred_N**	1	105	296	4633	3,5E-11	5,8E-15	1	0	CL0063
172	311	PF00732.19	GMC_oxred_N	208	295	296	7211	4,8E-19	8,1E-23	1	0	CL0063
364	494	PF05199.13	GMC_oxred_C	1	141	144	9663	1,8E-26	3E-30	1	0	
16	135	PF00732.19	GMC_oxred_N	1	101	296	4438	1,4E-10	2,3E-14	1	0	CL0063
153	291	PF00732.19	GMC_oxred_N	208	295	296	7005	2,1E-18	3,4E-22	1	0	CL0063
344	474	PF05199.13	GMC_oxred_C	1	141	144	9863	4,3E-27	7,3E-31	1	0	
8	268	PF00724.20	Oxidored_FMN	2	254	342	24611	5,8E-72	3,2E-76	1	0	CL0036
8	268	PF00724.20	Oxidored_FMN	2	254	342	24611	5,8E-72	3,2E-76	1	0	CL0036
46	427	PF01494.19	FAD_binding_3	2	343	349	24632	5,1E-72	2,9E-76	1	0	CL0063
158	335	PF01595.20	DUF21	1	176	176	14794	2,3E-42	3,8E-46	1	0	
494	600	PF03471.17	CorC_HlyC	2	80	81	7865	2,5E-21	4,2E-25	1	0	

350	409	PF00571.28	CBS	1	56	57	1679	7	0,000012	1	0	
418	474	PF00571.28	CBS	4	56	57	2754	0,000031	5,1E-09	1	0	
11	240	PF00743.19	FMO-like	3	215	532	10805	4E-30	2,2E-33	1	0	CL0063
246	396	PF00743.19	FMO-like	298	395	532	4539	3,9E-11	2,2E-14	1	0	CL0063
75	326	PF13738.6	Pyr_redox_3	54	284	305	3629	3,7E-08	2E-11	1	1	CL0063
12	308	PF07992.14	Pyr_redox_2	2	252	294	4700	1,9E-11	1,1E-14	1	1	CL0063
16	75	PF13450.6	NAD_binding_8	1	36	68	4321	3,4E-10	1,9E-13	1	1	CL0063
13	53	PF00890.24	FAD_binding_2	2	36	417	2861	0,0000073	4,1E-09	1	1	CL0063
12	49	PF03486.14	HI0933_like	2	36	409	2805	0,0000082	4,6E-09	1	1	CL0063
15	175	PF13454.6	NAD_binding_9	1	155	156	2343	0,00047	0,00000026	1	1	CL0063
21	163	PF01593.24	Amino_oxidase	2	28	452	2394	0,00022	0,00000012	1	1	CL0063
10	168	PF00743.19	FMO-like	3	135	532	6326	1,5E-16	7,5E-20	1	0	CL0063
15	80	PF13450.6	NAD_binding_8	1	35	68	4295	4,1E-10	2,1E-13	1	1	CL0063
12	52	PF00890.24	FAD_binding_2	2	37	417	3180	7,9E-07	3,9E-10	1	1	CL0063
11	50	PF03486.14	HI0933_like	2	36	409	3075	0,0000013	6,3E-10	1	1	CL0063
11	142	PF07992.14	Pyr_redox_2	2	37	294	2950	0,0000042	2,1E-09	1	1	CL0063
12	54	PF01266.24	DAO	1	36	352	2819	0,000014	6,9E-09	1	1	CL0063
20	165	PF01593.24	Amino_oxidase	2	120	452	2786	0,000014	7,2E-09	1	1	CL0063
10	47	PF01494.19	FAD_binding_3	3	34	349	2406	0,00019	9,8E-08	1	1	CL0063
12	56	PF00070.27	Pyr_redox	1	35	81	2362	0,00056	0,00000028	1	1	CL0063
52	193	PF00258.25	Flavodoxin_1	1	143	143	9316	1,7E-25	2,8E-29	1	0	CL0042
510	574	PF08608.12	Wyosine_form	2	61	63	8791	4,1E-24	6,8E-28	1	0	
326	508	PF04055.21	Radical_SAM	2	166	167	8771	1,1E-23	1,8E-27	1	0	CL0036
39	143	PF13426.7	**PAS_9**	11	102	104	5612	3,5E-14	5,9E-18	1	0	CL0183
259	365	PF13426.7	PAS_9	7	100	104	5481	9E-14	1,5E-17	1	0	CL0183
43	138	PF00989.25	PAS	23	101	113	2384	0,00033	5,6E-08	1	1	CL0183
270	362	PF00989.25	PAS	23	109	113	2775	0,00002	3,4E-09	1	1	CL0183
74	466	PF01134.22	**GIDA**	1	392	392	51700	3,3E-154	7,4E-158	1	0	CL0063
468	683	PF13932.6	GIDA_assoc	1	210	210	23498	9,9E-69	2,2E-72	1	0	

75	272	PF13460.6	NAD_binding_10	1	183	184	4543	7,9E-11	2,2E-14	1	0	CL0063
71	283	PF01370.21	Epimerase	2	241	241	4115	1,3E-09	3,7E-13	1	1	CL0063
71	309	PF05368.13	NmrA	2	223	233	4155	1,1E-09	2,9E-13	1	1	CL0063
72	184	PF01073.19	3Beta_HSD	2	75	280	2285	0,00038	0,0000001	1	1	CL0063
42	387	PF01494.19	FAD_binding_3	3	339	349	9659	1,7E-26	3,8E-30	1	0	CL0063
186	418	PF08491.10	SE	2	210	276	3206	0,0000006	1,3E-10	1	1	CL0063
47	106	PF13450.6	NAD_binding_8	1	45	68	2875	0,000011	2,5E-09	1	1	CL0063
44	237	PF05834.12	Lycopene_cycl	2	164	380	2467	0,00011	2,5E-08	1	1	CL0063
44	391	PF01494.19	FAD_binding_3	3	342	349	9422	9E-26	2,5E-29	1	0	CL0063
49	109	PF13450.6	NAD_binding_8	1	42	68	2642	0,000059	1,7E-08	1	1	CL0063
46	234	PF05834.12	Lycopene_cycl	2	145	380	2357	0,00024	6,7E-08	1	1	CL0063
188	401	PF08491.10	SE	2	189	276	2462	0,00011	3,1E-08	1	1	CL0063
46	93	PF00890.24	FAD_binding_2	2	35	417	2163	0,00096	0,00000027	1	1	CL0063
6	301	PF02219.17	MTHFR	1	287	287	42325	4,6E-126	2,6E-130	1	0	CL0086
117	290	PF00875.18	**DNA_photolyase**	1	154	164	15085	3,2E-43	1,8E-47	1	0	CL0039
35	175	PF01756.19	ACOX	6	138	180	5988	2,3E-15	1,3E-19	1	0	CL0087
163	340	PF01595.20	**DUF21**	1	176	176	14501	1,8E-41	3E-45	1	0	
499	605	PF03471.17	CorC_HlyC	2	80	81	8227	1,9E-22	3,2E-26	1	0	
355	414	PF00571.28	CBS	1	56	57	2039	53	0,0000088	1	0	
423	479	PF00571.28	CBS	1	56	57	2917	0,0000095	1,6E-09	1	0	
14	354	PF01070.18	FMN_dh	1	347	348	46012	4,2E-137	1,2E-140	1	0	CL0036
212	360	PF03060.15	NMO	146	257	331	2953	0,0000046	1,3E-09	1	1	CL0036
193	319	PF00478.25	IMPDH	146	237	345	2861	0,0000068	1,9E-09	1	1	CL0036
14	354	PF01070.18	FMN_dh	1	347	348	46041	3,5E-137	1,2E-140	1	0	CL0036
211	357	PF03060.15	NMO	141	257	331	3498	0,0000001	3,4E-11	1	1	CL0036
202	318	PF00478.25	IMPDH	134	237	345	2974	0,0000031	1E-09	1	1	CL0036
259	316	PF01645.17	Glu_synthase	271	306	368	2108	14	0,00000049	1	1	CL0036
8	286	PF01207.17	Dus	1	236	310	18290	7,6E-53	4,2E-57	1	0	CL0036
46	323	PF00732.19	**GMC_oxred_N**	1	295	296	10837	4,4E-30	4,9E-34	1	0	CL0063
412	559	PF05199.13	GMC_oxred_C	1	142	144	9588	3,1E-26	3,4E-30	1	0	

55	379	PF01207.17	**Dus**	2	297	310	21463	1,7E-62	9,2E-67	1	0	CL0036
5	152	PF03358.15	FMN_red	18	145	155	4478	1E-10	1,2E-14	1	0	CL0042
3	157	PF02525.17	Flavodoxin_2	2	138	198	2655	0,000044	4,9E-09	1	1	CL0042
74	223	PF03358.15	FMN_red	19	145	155	4407	1,7E-10	1,9E-14	1	0	CL0042
77	209	PF00258.25	Flavodoxin_1	1	142	143	2664	0,000055	6,2E-09	1	1	CL0042
341	621	PF01207.17	**Dus**	2	270	310	16877	1,5E-48	2,5E-52	1	0	CL0036
13	261	PF00743.19	FMO-like	2	215	532	12245	1,7E-34	1,1E-37	1	0	CL0063
254	416	PF00743.19	FMO-like	298	443	532	5313	1,7E-13	1,1E-16	1	0	CL0063
80	327	PF13738.6	Pyr_redox_3	52	286	305	5449	1E-13	6,4E-17	1	1	CL0063
14	320	PF07992.14	Pyr_redox_2	1	253	294	7485	6,4E-20	3,9E-23	1	1	CL0063
18	66	PF13450.6	NAD_binding_8	1	36	68	3810	1,3E-08	8,2E-12	1	1	CL0063
14	52	PF03486.14	HI0933_like	2	36	409	2671	0,000021	1,3E-08	1	1	CL0063
15	55	PF00890.24	FAD_binding_2	2	39	417	2781	0,000013	7,8E-09	1	1	CL0063
102	253	PF13434.6	K_oxygenase	97	220	342	2537	0,000072	4,4E-08	1	1	CL0063
13	49	PF01494.19	FAD_binding_3	3	34	349	2669	0,000031	1,9E-08	1	1	CL0063
5	371	PF01494.19	FAD_binding_3	4	344	349	4991	2,7E-12	1,5E-16	1	0	CL0063
193	300	PF00175.21	NAD_binding_1	1	108	109	10419	5,6E-29	9,4E-33	1	0	CL0091
80	183	PF00970.24	FAD_binding_6	2	99	99	8891	2,2E-24	3,6E-28	1	0	CL0076
10	255	PF00743.19	FMO-like	2	220	532	8442	5,8E-23	2,6E-26	1	0	CL0063
251	459	PF00743.19	FMO-like	265	446	532	3531	4,4E-08	2E-11	1	0	CL0063
11	264	PF07992.14	Pyr_redox_2	2	178	294	2731	0,000019	8,7E-09	1	1	CL0063
15	87	PF13450.6	NAD_binding_8	1	52	68	3070	0,0000027	1,2E-09	1	1	CL0063
13	335	PF01207.17	**Dus**	2	291	310	19075	3,1E-55	1,7E-59	1	0	CL0036
46	321	PF00732.19	GMC_oxred_N	1	295	296	10963	1,8E-30	3E-34	1	0	CL0063
421	568	PF05199.13	GMC_oxred_C	1	142	144	9548	4,1E-26	6,8E-30	1	0	
56	199	PF04030.14	ALO	130	259	260	5090	1,8E-12	1E-16	1	0	CL0277
4	154	PF03358.15	FMN_red	2	146	155	3777	1,5E-08	8,4E-13	1	0	CL0042
91	387	PF01207.17	Dus	1	290	310	24387	2,1E-71	1,2E-75	1	0	CL0036
