# Supplementary material for: The Flavoproteome of the Model Plant Arabidopsis thaliana
Source: Int J Mol Sci. 2020 Jul 28;21(15):5371. doi: 10.3390/ijms21155371 (PMC7432721; doi:10.3390/ijms21155371)
Supplement: Supplementary file 1 [file ijms-21-05371-s001.zip › Table S5.pdf]

Table S5 GO term enrichment of the flavoproteome of Arabidopsis thaliana The p-value cut off is $p < 0.05$

**GO term**	**Ontology**	percentage of genes	- log10 p-valu	entries
GO:0055114	oxidation-reduction process	86,35	223,62	// AT5G49730 // AT3G54660 // AT2G24580 // AT5G03630 // AT4G38540 // AT4G05020 // AT4G142
GO:0044710	single-organism metabolic process	88,35	149,80	// AT5G49730 // AT3G54660 // AT2G24580 // AT5G03630 // AT4G38540 // AT4G05020 // AT4G142
GO:0044699	single-organism process	91,57	81,57	// AT5G49730 // AT3G54660 // AT2G24580 // AT5G03630 // AT4G38540 // AT4G05020 // AT4G142
GO:0008152	metabolic process	93,17	64,00	// AT5G49730 // AT3G54660 // AT2G24580 // AT5G03630 // AT4G38540 // AT4G05020 // AT4G142
GO:0044281	small molecule metabolic process	27,31	26,85	// AT2G46760 // AT2G44160 // AT2G18450 // AT1G62540 // AT1G06310 // AT3G51840 // AT2G467
GO:0043436	oxoacid metabolic process	20,48	23,59	// AT2G46760 // AT2G44160 // AT1G62540 // AT1G06310 // AT3G51840 // AT2G46740 // AT4G323
GO:0006082	organic acid metabolic process	21,29	22,96	// AT2G46760 // AT2G44160 // AT1G62540 // AT1G06310 // AT3G51840 // AT2G46740 // AT4G323
GO:0042445	hormone metabolic process	9,24	19,82	// AT1G04610 // AT3G63440 // AT5G25620 // AT5G21482 // AT2G19500 // AT5G43890 // AT1G214
GO:0019752	carboxylic acid metabolic process	17,27	19,00	// AT2G46760 // AT2G44160 // AT1G06310 // AT3G51840 // AT2G46740 // AT4G32360 // AT1G729

GO:0044711	single-organism biosynthetic process	23,69	18,46	// AT2G46760 // AT1G62540 // AT4G14210 // AT2G46740 // AT1G06820 // AT4G32360 // AT3G479[illegible]
GO:0009851	auxin biosynthetic process	6,02	16,52	// AT1G04610 // AT1G48910 // AT1G04580 // AT2G33230 // AT1G21430 // AT4G13260 // AT5G438[illegible]
GO:0010817	regulation of hormone levels	9,64	16,47	// AT1G04610 // AT3G63440 // AT5G25620 // AT5G21482 // AT2G19500 // AT4G08920 // AT5G438[illegible]
GO:0046394	carboxylic acid biosynthetic process	11,24	14,92	// AT2G46760 // AT2G46740 // AT4G32360 // AT3G47930 // AT5G53460 // AT5G11540 // AT2G060[illegible]
GO:0009850	auxin metabolic process	6,02	14,74	// AT1G04610 // AT1G48910 // AT1G04580 // AT2G33230 // AT1G21430 // AT4G13260 // AT5G438[illegible]
GO:0042446	hormone biosynthetic process	6,43	13,59	// AT1G04610 // AT1G48910 // AT1G04580 // AT2G33230 // AT1G21430 // AT4G13260 // AT5G438[illegible]
GO:0044283	small molecule biosynthetic process	12,45	13,16	// AT2G46760 // AT2G46740 // AT4G32360 // AT3G47930 // AT5G53460 // AT5G49970 // AT2G060[illegible]
GO:0016053	organic acid biosynthetic process	11,24	12,96	// AT2G46760 // AT2G46740 // AT4G32360 // AT3G47930 // AT5G53460 // AT5G11540 // AT2G060[illegible]
GO:0065008	regulation of biological quality	16,47	12,12	// AT1G04610 // AT3G54660 // AT3G24170 // AT2G41680 // AT5G25620 // AT3G63440 // AT5G214[illegible]

GO:0072593	reactive oxygen species metabolic process	6,43	11,08	// AT3G14420 // AT1G77760 // AT1G37130 // AT4G08920 // AT2G41680 // AT4G18360 // AT3G528
GO:0009823	cytokinin catabolic process	2,81	10,96	// AT2G19500 // AT1G75450 // AT2G41510 // AT5G56970 // AT4G29740 // AT3G63440 // AT5G214
GO:0044237	cellular metabolic process	55,42	10,49	// AT3G54660 // AT2G24580 // AT4G14210 // AT2G46740 // AT1G06820 // AT4G32360 // AT5G510
GO:0046395	carboxylic acid catabolic process	5,22	10,43	// AT1G50940 // AT1G06290 // AT1G06310 // AT3G51840 // AT5G38710 // AT3G06690 // AT3G307
GO:0042447	hormone catabolic process	2,81	10,43	// AT2G19500 // AT1G75450 // AT2G41510 // AT5G56970 // AT4G29740 // AT3G63440 // AT5G214
GO:0044282	small molecule catabolic process	6,02	10,29	// AT1G50940 // AT1G06290 // AT1G06310 // AT3G51840 // AT3G10370 // AT5G38710 // AT3G066
GO:0042364	water-soluble vitamin biosynthetic process	4,42	9,96	// AT2G46760 // AT2G46750 // AT2G46740 // AT2G46580 // AT4G32360 // AT1G32300 // AT3G479
GO:0006767	water-soluble vitamin metabolic process	4,42	9,85	// AT2G46760 // AT2G46750 // AT2G46740 // AT2G46580 // AT4G32360 // AT1G32300 // AT3G479

GO:0016054	organic acid catabolic process	5,22	9,72	// AT1G50940 // AT1G06290 // AT1G06310 // AT3G51840 // AT5G38710 // AT3G06690 // AT3G30[illegible]
GO:1903409	reactive oxygen species biosynthetic process	3,21	9,70	// AT3G14420 // AT1G77760 // AT1G37130 // AT4G18360 // AT1G64060 // AT3G14150 // AT3G14[illegible]
GO:0044763	single-organism cellular process	41,37	9,68	// AT3G54660 // AT5G56470 // AT4G14210 // AT2G46740 // AT1G06820 // AT4G32360 // AT5G51[illegible]
GO:0019852	L-ascorbic acid metabolic process	3,21	9,47	// AT2G46760 // AT2G46750 // AT2G46740 // AT1G32300 // AT3G47930 // AT5G56470 // AT5G56[illegible]
GO:0019853	L-ascorbic acid biosynthetic process	3,21	9,47	// AT2G46760 // AT2G46750 // AT2G46740 // AT1G32300 // AT3G47930 // AT5G56470 // AT5G56[illegible]
GO:0009110	vitamin biosynthetic process	4,42	9,24	// AT2G46760 // AT2G46750 // AT2G46740 // AT2G46580 // AT4G32360 // AT1G32300 // AT3G47[illegible]
GO:0006766	vitamin metabolic process	4,42	9,15	// AT2G46760 // AT2G46750 // AT2G46740 // AT2G46580 // AT4G32360 // AT1G32300 // AT3G47[illegible]
GO:0019395	fatty acid oxidation	3,61	9,10	// AT1G50940 // AT1G06290 // AT1G06310 // AT3G51840 // AT3G06690 // AT1G72970 // AT5G65[illegible]
GO:0018298	protein-chromophore linkage	3,61	8,96	// AT3G45780 // AT4G08920 // AT2G47590 // AT1G68050 // AT2G18915 // AT1G04400 // AT5G58[illegible]

GO:0045454	cell redox homeostasis	5,22	8,77 // AT3G54660 // AT3G24170 // AT2G17420 // AT2G41680 // AT3G52880 // AT5G03630 // AT4G354
GO:0051186	cofactor metabolic process	7,63	8,42 // AT4G01690 // AT3G59970 // AT2G24580 // AT4G08920 // AT1G48605 // AT5G14760 // AT3G103
GO:0034440	lipid oxidation	3,61	8,35 // AT1G50940 // AT1G06290 // AT1G06310 // AT3G51840 // AT3G06690 // AT1G72970 // AT5G651
GO:0009308	amine metabolic process	4,42	8,34 // AT2G19500 // AT1G75450 // AT3G59050 // AT2G41510 // AT5G56970 // AT1G65840 // AT4G297
GO:0050665	hydrogen peroxide biosynthetic process	2,41	8,33 // AT3G14420 // AT4G18360 // AT1G64060 // AT3G14150 // AT3G14415 // AT3G14130
GO:0006635	fatty acid beta-oxidation	3,21	8,16 // AT1G50940 // AT1G06290 // AT1G06310 // AT3G51840 // AT3G06690 // AT5G65110 // AT4G167
GO:0009062	fatty acid catabolic process	3,21	8,09 // AT1G50940 // AT1G06290 // AT1G06310 // AT3G51840 // AT3G06690 // AT5G65110 // AT4G167
GO:0022900	electron transport chain	4,42	7,96 // AT5G08530 // AT4G36400 // AT5G17770 // AT3G10370 // AT4G32360 // AT2G18450 // AT5G667
GO:0006537	glutamate biosynthetic process	2,01	7,92 // AT5G53460 // AT2G41220 // AT5G04140 // AT3G30775 // AT5G38710
GO:0032787	monocarboxylic acid metabolic process	8,03	7,70 // AT1G50940 // AT1G04580 // AT1G06290 // AT4G36400 // AT1G06310 // AT3G51840 // AT5G209

GO:0043650	dicarboxylic acid biosynthetic process	2,41	7,57	// AT2G41220 // AT3G30775 // AT5G04140 // AT5G38710 // AT5G53460 // AT1G48850
GO:1901565	organonitrogen compound catabolic process	4,82	7,28	// AT5G13700 // AT3G59050 // AT4G34900 // AT1G65840 // AT3G30775 // AT2G43020 // AT5G63[illegible]
GO:0072329	monocarboxylic acid catabolic process	3,21	7,29	// AT1G50940 // AT1G06290 // AT1G06310 // AT3G51840 // AT3G06690 // AT5G65110 // AT4G16[illegible]
GO:0009056	catabolic process	14,06	7,21	// AT1G06310 // AT3G51840 // AT2G41680 // AT3G06690 // AT3G63440 // AT5G63910 // AT3G27[illegible]
GO:0042743	hydrogen peroxide metabolic process	4,02	7,19	// AT3G14420 // AT4G08920 // AT2G41680 // AT4G18360 // AT3G52880 // AT1G64060 // AT3G14[illegible]
GO:0009690	cytokinin metabolic process	2,81	7,14	// AT2G19500 // AT1G75450 // AT2G41510 // AT5G56970 // AT4G29740 // AT3G63440 // AT5G21[illegible]
GO:0009854	oxidative photosynthetic carbon pathway	2,01	7,05	// AT4G18360 // AT3G14420 // AT3G14130 // AT3G14415 // AT3G14150
GO:0034754	cellular hormone metabolic process	2,81	6,85	// AT2G19500 // AT1G75450 // AT2G41510 // AT5G56970 // AT4G29740 // AT3G63440 // AT5G21[illegible]

GO:0022904	respiratory electron transport chain	2,81	6,66	// AT5G08530 // AT4G36400 // AT3G10370 // AT2G18450 // AT5G08740 // AT5G66760 // AT2G43[illegible]
GO:0019725	cellular homeostasis	6,02	6,64	// AT3G54660 // AT3G24170 // AT2G17420 // AT4G08920 // AT2G41680 // AT3G52880 // AT1G04[illegible]
GO:0030258	lipid modification	3,61	6,62	// AT1G50940 // AT1G06290 // AT1G06310 // AT3G51840 // AT3G06690 // AT1G72970 // AT5G65[illegible]
GO:0019758	glycosinolate biosynthetic process	2,81	6,54	// AT1G65860 // AT1G62540 // AT1G12130 // AT1G12160 // AT1G12140 // AT1G62560 // AT1G62[illegible]
GO:0019761	glucosinolate biosynthetic process	2,81	6,54	// AT1G65860 // AT1G62540 // AT1G12130 // AT1G12160 // AT1G12140 // AT1G62560 // AT1G62[illegible]
GO:0044242	cellular lipid catabolic process	3,21	6,26	// AT1G50940 // AT1G06290 // AT1G06310 // AT3G51840 // AT3G06690 // AT5G65110 // AT4G16[illegible]
GO:0009987	cellular process	60,64	6,22	// AT3G54660 // AT2G24580 // AT5G03630 // AT4G14210 // AT2G46740 // AT1G06820 // AT4G32[illegible]
GO:0009853	photorespiration	2,81	5,89	// AT3G14420 // AT4G36400 // AT4G18360 // AT5G04140 // AT3G14130 // AT3G14415 // AT3G14[illegible]
GO:0006950	response to stress	23,29	5,82	// AT3G54660 // AT4G24520 // AT5G20080 // AT4G38540 // AT2G41680 // AT2G34810 // AT4G18[illegible]
GO:0006536	glutamate metabolic process	2,01	5,74	// AT5G53460 // AT2G41220 // AT5G04140 // AT3G30775 // AT5G38710
GO:0042592	homeostatic process	7,23	5,62	// AT3G54660 // AT3G24170 // AT2G17420 // AT4G08920 // AT2G41680 // AT3G52880 // AT1G48[illegible]

GO:0009688	abscisic acid biosynthetic process	2,01	5,57	// AT1G04580 // AT3G43600 // AT2G27150 // AT5G67030 // AT5G20960
GO:0055114	oxidation-reduction process	-3,93	-10,02	// AT5G49730 // AT3G54660 // AT2G24580 // AT5G03630 // AT4G38540 // AT4G05020 // AT4G142
GO:0044710	single-organism metabolic process	-4,48	-10,85	// AT5G49730 // AT3G54660 // AT2G24580 // AT5G03630 // AT4G38540 // AT4G05020 // AT4G142
GO:0044699	single-organism process	-5,03	-11,69	// AT5G49730 // AT3G54660 // AT2G24580 // AT5G03630 // AT4G38540 // AT4G05020 // AT4G142
GO:0008152	metabolic process	-5,57	-12,53	// AT5G49730 // AT3G54660 // AT2G24580 // AT5G03630 // AT4G38540 // AT4G05020 // AT4G142
GO:0044281	small molecule metabolic process	-6,12	-13,37	// AT2G46760 // AT2G44160 // AT2G18450 // AT1G62540 // AT1G06310 // AT3G51840 // AT2G467
GO:0043436	oxoacid metabolic process	-6,67	-14,20	// AT2G46760 // AT2G44160 // AT1G62540 // AT1G06310 // AT3G51840 // AT2G46740 // AT4G323
GO:0006082	organic acid metabolic process	-7,21	-15,04	// AT2G46760 // AT2G44160 // AT1G62540 // AT1G06310 // AT3G51840 // AT2G46740 // AT4G323
GO:0042445	hormone metabolic process	-7,76	-15,88	// AT1G04610 // AT3G63440 // AT5G25620 // AT5G21482 // AT2G19500 // AT5G43890 // AT1G214
GO:0019752	carboxylic acid metabolic process	-8,31	-16,72	// AT2G46760 // AT2G44160 // AT1G06310 // AT3G51840 // AT2G46740 // AT4G32360 // AT1G72

GO:0044711	single-organism biosynthetic process	-8,86	-17,56	// AT2G46760 // AT1G62540 // AT4G14210 // AT2G46740 // AT1G06820 // AT4G32360 // AT3G47[illegible]
GO:0055114	oxidation-reduction process	-9,40	-18,39	// AT5G49730 // AT3G54660 // AT2G24580 // AT5G03630 // AT4G38540 // AT4G05020 // AT4G14[illegible]
GO:0044710	single-organism metabolic process	-9,95	-19,23	// AT5G49730 // AT3G54660 // AT2G24580 // AT5G03630 // AT4G38540 // AT4G05020 // AT4G14[illegible]
GO:0044699	single-organism process	-10,50	-20,07	// AT5G49730 // AT3G54660 // AT2G24580 // AT5G03630 // AT4G38540 // AT4G05020 // AT4G14[illegible]
GO:0008152	metabolic process	-11,04	-20,91	// AT5G49730 // AT3G54660 // AT2G24580 // AT5G03630 // AT4G38540 // AT4G05020 // AT4G14[illegible]
GO:0044281	small molecule metabolic process	-11,59	-21,74	// AT2G46760 // AT2G44160 // AT2G18450 // AT1G62540 // AT1G06310 // AT3G51840 // AT2G46[illegible]
GO:0043436	oxoacid metabolic process	-12,14	-22,58	// AT2G46760 // AT2G44160 // AT1G62540 // AT1G06310 // AT3G51840 // AT2G46740 // AT4G32[illegible]
GO:0006082	organic acid metabolic process	-12,68	-23,42	// AT2G46760 // AT2G44160 // AT1G62540 // AT1G06310 // AT3G51840 // AT2G46740 // AT4G32[illegible]
GO:0042445	hormone metabolic process	-13,23	-24,26	// AT1G04610 // AT3G63440 // AT5G25620 // AT5G21482 // AT2G19500 // AT5G43890 // AT1G21[illegible]
GO:0019752	carboxylic acid metabolic process	-13,78	-25,09	// AT2G46760 // AT2G44160 // AT1G06310 // AT3G51840 // AT2G46740 // AT4G32360 // AT1G72[illegible]

GO:0044711	single-organism biosynthetic process	-14,32	-25,93	// AT2G46760 // AT1G62540 // AT4G14210 // AT2G46740 // AT1G06820 // AT4G32360 // AT3G47[illegible]
GO:0009851	auxin biosynthetic process	-14,87	-26,77	// AT1G04610 // AT1G48910 // AT1G04580 // AT2G33230 // AT1G21430 // AT4G13260 // AT5G43[illegible]
GO:0010817	regulation of hormone levels	-15,42	-27,61	// AT1G04610 // AT3G63440 // AT5G25620 // AT5G21482 // AT2G19500 // AT4G08920 // AT5G43[illegible]
GO:0046394	carboxylic acid biosynthetic process	-15,97	-28,45	// AT2G46760 // AT2G46740 // AT4G32360 // AT3G47930 // AT5G53460 // AT5G11540 // AT2G06[illegible]
GO:0009850	auxin metabolic process	-16,51	-29,28	// AT1G04610 // AT1G48910 // AT1G04580 // AT2G33230 // AT1G21430 // AT4G13260 // AT5G43[illegible]
GO:0042446	hormone biosynthetic process	-17,06	-30,12	// AT1G04610 // AT1G48910 // AT1G04580 // AT2G33230 // AT1G21430 // AT4G13260 // AT5G43[illegible]
GO:0044283	small molecule biosynthetic process	-17,61	-30,96	// AT2G46760 // AT2G46740 // AT4G32360 // AT3G47930 // AT5G53460 // AT5G49970 // AT2G06[illegible]
GO:0016053	organic acid biosynthetic process	-18,15	-31,80	// AT2G46760 // AT2G46740 // AT4G32360 // AT3G47930 // AT5G53460 // AT5G11540 // AT2G06[illegible]
GO:0065008	regulation of biological quality	-18,70	-32,63	// AT1G04610 // AT3G54660 // AT3G24170 // AT2G41680 // AT5G25620 // AT3G63440 // AT5G21[illegible]

GO:0072593	reactive oxygen species metabolic process	-19,25	-33,47	// AT3G14420 // AT1G77760 // AT1G37130 // AT4G08920 // AT2G41680 // AT4G18360 // AT3G52[illegible]
GO:0009823	cytokinin catabolic process	-19,79	-34,31	// AT2G19500 // AT1G75450 // AT2G41510 // AT5G56970 // AT4G29740 // AT3G63440 // AT5G21[illegible]
GO:0044237	cellular metabolic process	-20,34	-35,15	// AT3G54660 // AT2G24580 // AT4G14210 // AT2G46740 // AT1G06820 // AT4G32360 // AT5G51[illegible]
GO:0046395	carboxylic acid catabolic process	-20,89	-35,98	// AT1G50940 // AT1G06290 // AT1G06310 // AT3G51840 // AT5G38710 // AT3G06690 // AT3G30[illegible]
GO:0042447	hormone catabolic process	-21,44	-36,82	// AT2G19500 // AT1G75450 // AT2G41510 // AT5G56970 // AT4G29740 // AT3G63440 // AT5G21[illegible]
GO:0044282	small molecule catabolic process	-21,98	-37,66	// AT1G50940 // AT1G06290 // AT1G06310 // AT3G51840 // AT3G10370 // AT5G38710 // AT3G06[illegible]
GO:0042364	water-soluble vitamin biosynthetic process	-22,53	-38,50	// AT2G46760 // AT2G46750 // AT2G46740 // AT2G46580 // AT4G32360 // AT1G32300 // AT3G47[illegible]
GO:0006767	water-soluble vitamin metabolic process	-23,08	-39,34	// AT2G46760 // AT2G46750 // AT2G46740 // AT2G46580 // AT4G32360 // AT1G32300 // AT3G47[illegible]

GO:0016054	organic acid catabolic process	-23,62	-40,17	// AT1G50940 // AT1G06290 // AT1G06310 // AT3G51840 // AT5G38710 // AT3G06690 // AT3G307
GO:1903409	reactive oxygen species biosynthetic process	-24,17	-41,01	// AT3G14420 // AT1G77760 // AT1G37130 // AT4G18360 // AT1G64060 // AT3G14150 // AT3G144
GO:0044763	single-organism cellular process	-24,72	-41,85	// AT3G54660 // AT5G56470 // AT4G14210 // AT2G46740 // AT1G06820 // AT4G32360 // AT5G51(
GO:0019852	L-ascorbic acid metabolic process	-25,26	-42,69	// AT2G46760 // AT2G46750 // AT2G46740 // AT1G32300 // AT3G47930 // AT5G56470 // AT5G564
GO:0019853	L-ascorbic acid biosynthetic process	-25,81	-43,52	// AT2G46760 // AT2G46750 // AT2G46740 // AT1G32300 // AT3G47930 // AT5G56470 // AT5G564
GO:0009110	vitamin biosynthetic process	-26,36	-44,36	// AT2G46760 // AT2G46750 // AT2G46740 // AT2G46580 // AT4G32360 // AT1G32300 // AT3G479
GO:0006766	vitamin metabolic process	-26,91	-45,20	// AT2G46760 // AT2G46750 // AT2G46740 // AT2G46580 // AT4G32360 // AT1G32300 // AT3G479
GO:0019395	fatty acid oxidation	-27,45	-46,04	// AT1G50940 // AT1G06290 // AT1G06310 // AT3G51840 // AT3G06690 // AT1G72970 // AT5G651
GO:0018298	protein-chromophore linkage	-28,00	-46,88	// AT3G45780 // AT4G08920 // AT2G47590 // AT1G68050 // AT2G18915 // AT1G04400 // AT5G581

GO:0045454	cell redox homeostasis	-28,55	-47,71	// AT3G54660 // AT3G24170 // AT2G17420 // AT2G41680 // AT3G52880 // AT5G03630 // AT4G354
GO:0051186	cofactor metabolic process	-29,09	-48,55	// AT4G01690 // AT3G59970 // AT2G24580 // AT4G08920 // AT1G48605 // AT5G14760 // AT3G103
GO:0034440	lipid oxidation	-29,64	-49,39	// AT1G50940 // AT1G06290 // AT1G06310 // AT3G51840 // AT3G06690 // AT1G72970 // AT5G651
GO:0009308	amine metabolic process	-30,19	-50,23	// AT2G19500 // AT1G75450 // AT3G59050 // AT2G41510 // AT5G56970 // AT1G65840 // AT4G297
GO:0050665	hydrogen peroxide biosynthetic process	-30,73	-51,06	// AT3G14420 // AT4G18360 // AT1G64060 // AT3G14150 // AT3G14415 // AT3G14131
GO:0006635	fatty acid beta-oxidation	-31,28	-51,90	// AT1G50940 // AT1G06290 // AT1G06310 // AT3G51840 // AT3G06690 // AT5G65110 // AT4G167
GO:0009062	fatty acid catabolic process	-31,83	-52,74	// AT1G50940 // AT1G06290 // AT1G06310 // AT3G51840 // AT3G06690 // AT5G65110 // AT4G167
GO:0022900	electron transport chain	-32,38	-53,58	// AT5G08530 // AT4G36400 // AT5G17770 // AT3G10370 // AT4G32360 // AT2G18450 // AT5G667
GO:0006537	glutamate biosynthetic process	-32,92	-54,41	// AT5G53460 // AT2G41220 // AT5G04140 // AT3G30775 // AT5G38711
GO:0032787	monocarboxylic acid metabolic process	-33,47	-55,25	// AT1G50940 // AT1G04580 // AT1G06290 // AT4G36400 // AT1G06310 // AT3G51840 // AT5G209

GO:0043650	dicarboxylic acid biosynthetic process	-34,02	-56,09	// AT2G41220 // AT3G30775 // AT5G04140 // AT5G38710 // AT5G53460 // AT1G48851
GO:1901565	organonitrogen compound catabolic process	-34,56	-56,93	// AT5G13700 // AT3G59050 // AT4G34900 // AT1G65840 // AT3G30775 // AT2G43020 // AT5G63[illegible]
GO:0072329	monocarboxylic acid catabolic process	-35,11	-57,77	// AT1G50940 // AT1G06290 // AT1G06310 // AT3G51840 // AT3G06690 // AT5G65110 // AT4G16[illegible]
GO:0009056	catabolic process	-35,66	-58,60	// AT1G06310 // AT3G51840 // AT2G41680 // AT3G06690 // AT3G63440 // AT5G63910 // AT3G27[illegible]
GO:0042743	hydrogen peroxide metabolic process	-36,20	-59,44	// AT3G14420 // AT4G08920 // AT2G41680 // AT4G18360 // AT3G52880 // AT1G64060 // AT3G14[illegible]
GO:0009690	cytokinin metabolic process	-36,75	-60,28	// AT2G19500 // AT1G75450 // AT2G41510 // AT5G56970 // AT4G29740 // AT3G63440 // AT5G21[illegible]
GO:0009854	oxidative photosynthetic carbon pathway	-37,30	-61,12	// AT4G18360 // AT3G14420 // AT3G14130 // AT3G14415 // AT3G14151
GO:0034754	cellular hormone metabolic process	-37,84	-61,95	// AT2G19500 // AT1G75450 // AT2G41510 // AT5G56970 // AT4G29740 // AT3G63440 // AT5G21[illegible]

GO:0022904	respiratory electron transport chain	-38,39	-62,79	// AT5G08530 // AT4G36400 // AT3G10370 // AT2G18450 // AT5G08740 // AT5G66760 // AT2G43[illegible]
GO:0019725	cellular homeostasis	-38,94	-63,63	// AT3G54660 // AT3G24170 // AT2G17420 // AT4G08920 // AT2G41680 // AT3G52880 // AT1G04[illegible]
GO:0030258	lipid modification	-39,49	-64,47	// AT1G50940 // AT1G06290 // AT1G06310 // AT3G51840 // AT3G06690 // AT1G72970 // AT5G65[illegible]
GO:0019758	glycosinolate biosynthetic process	-40,03	-65,30	// AT1G65860 // AT1G62540 // AT1G12130 // AT1G12160 // AT1G12140 // AT1G62560 // AT1G62[illegible]
GO:0019761	glucosinolate biosynthetic process	-40,58	-66,14	// AT1G65860 // AT1G62540 // AT1G12130 // AT1G12160 // AT1G12140 // AT1G62560 // AT1G62[illegible]
GO:0044242	cellular lipid catabolic process	-41,13	-66,98	// AT1G50940 // AT1G06290 // AT1G06310 // AT3G51840 // AT3G06690 // AT5G65110 // AT4G16[illegible]
GO:0009987	cellular process	-41,67	-67,82	// AT3G54660 // AT2G24580 // AT5G03630 // AT4G14210 // AT2G46740 // AT1G06820 // AT4G32[illegible]
GO:0009853	photorespiration	-42,22	-68,66	// AT3G14420 // AT4G36400 // AT4G18360 // AT5G04140 // AT3G14130 // AT3G14415 // AT3G14[illegible]
GO:0006950	response to stress	-42,77	-69,49	// AT3G54660 // AT4G24520 // AT5G20080 // AT4G38540 // AT2G41680 // AT2G34810 // AT4G18[illegible]
GO:0006536	glutamate metabolic process	-43,31	-70,33	// AT5G53460 // AT2G41220 // AT5G04140 // AT3G30775 // AT5G38711
GO:0042592	homeostatic process	-43,86	-71,17	// AT3G54660 // AT3G24170 // AT2G17420 // AT4G08920 // AT2G41680 // AT3G52880 // AT1G48[illegible]

GO:0009688	abscisic acid biosynthetic process	-44,41	-72,01	// AT1G04580 // AT3G43600 // AT2G27150 // AT5G67030 // AT5G20961
GO:0055114	oxidation-reduction process	-44,96	-72,84	// AT5G49730 // AT3G54660 // AT2G24580 // AT5G03630 // AT4G38540 // AT4G05020 // AT4G142

210 // AT2G46740 // AT1G06820 // AT4G32360 // AT2G35660 // AT5G51060 // AT5G44360 // AT3G63440 // AT5G21482 // AT3G14415 // AT5G11540 // AT4G36400 // AT5

210 // AT2G46740 // AT1G06820 // AT4G32360 // AT2G35660 // AT5G51060 // AT5G44360 // AT3G63440 // AT5G21482 // AT3G14415 // AT5G11540 // AT4G36400 // AT5

210 // AT2G46740 // AT1G06820 // AT4G32360 // AT2G35660 // AT5G51060 // AT5G44360 // AT3G63440 // AT5G21482 // AT3G14415 // AT5G11540 // AT4G36400 // AT5

210 // AT2G46740 // AT1G06820 // AT4G32360 // AT2G35660 // AT5G51060 // AT5G44360 // AT3G63440 // AT5G21482 // AT3G14415 // AT5G11540 // AT4G36400 // AT5

740 // AT4G32360 // AT1G72970 // AT5G49970 // AT4G34890 // AT1G12140 // AT5G53460 // AT5G11540 // AT5G08530 // AT4G36400 // AT4G08920 // AT5G14760 // AT3

360 // AT1G72970 // AT1G12160 // AT1G12140 // AT5G53460 // AT5G11540 // AT1G50940 // AT4G36400 // AT2G06050 // AT2G41220 // AT1G32300 // AT1G77760 // AT5

360 // AT1G72970 // AT1G12160 // AT1G12140 // AT5G53460 // AT5G11540 // AT1G50940 // AT4G36400 // AT2G06050 // AT2G41220 // AT1G32300 // AT1G77760 // AT5

430 // AT2G27150 // AT1G04580 // AT2G41510 // AT1G04180 // AT4G32540 // AT4G29740 // AT2G33230 // AT5G56970 // AT4G13260 // AT5G11320 // AT1G48910 // AT3

970 // AT5G53460 // AT5G11540 // AT1G50940 // AT4G36400 // AT2G06050 // AT2G41220 // AT1G32300 // AT5G65110 // AT2G27150 // AT5G56490 // AT1G76680 // AT3

930 // AT1G12160 // AT2G35660 // AT4G37760 // AT1G12140 // AT5G08740 // AT5G53460 // AT5G49970 // AT4G01690 // AT4G15760 // AT5G14760 // AT5G24160 // AT3

390 // AT4G28720 // AT5G11320 // AT1G04180 // AT2G27150 // AT3G43600 // AT5G25620 // AT4G32540 // AT5G20960

390 // AT1G21430 // AT2G27150 // AT1G04580 // AT2G41510 // AT1G04180 // AT4G32540 // AT4G29740 // AT2G33230 // AT5G56970 // AT4G13260 // AT5G11320 // AT1

050 // AT2G41220 // AT1G32300 // AT2G27150 // AT5G56490 // AT1G76680 // AT1G04580 // AT2G46750 // AT3G30775 // AT3G59970 // AT5G04140 // AT5G56470 // AT4

390 // AT4G28720 // AT5G11320 // AT1G04180 // AT2G27150 // AT3G43600 // AT5G25620 // AT4G32540 // AT5G20960

390 // AT4G28720 // AT5G11320 // AT1G04180 // AT2G27150 // AT3G43600 // AT5G25620 // AT3G19820 // AT4G32540 // AT5G20960

050 // AT2G41220 // AT1G32300 // AT2G27150 // AT5G56490 // AT1G76680 // AT1G04580 // AT2G46750 // AT2G46580 // AT3G30775 // AT3G59970 // AT5G04140 // AT5

050 // AT2G41220 // AT1G32300 // AT2G27150 // AT5G56490 // AT1G76680 // AT1G04580 // AT2G46750 // AT3G30775 // AT3G59970 // AT5G04140 // AT5G56470 // AT4

482 // AT2G19500 // AT4G16155 // AT4G08920 // AT1G01580 // AT1G21430 // AT3G52880 // AT4G28720 // AT2G27150 // AT3G17240 // AT3G16950 // AT1G04580 // AT2

380 // AT1G04400 // AT5G47910 // AT1G64060 // AT3G14150 // AT3G27820 // AT4G34890 // AT4G35460 // AT3G14415 // AT3G14130

482

060 // AT3G63440 // AT5G21482 // AT3G14415 // AT5G11540 // AT4G36400 // AT5G17770 // AT4G16310 // AT3G52880 // AT1G32300 // AT1G20020 // AT5G49740 // AT3

775 // AT5G65110 // AT5G43430 // AT4G16760 // AT2G43400 // AT2G35690 // AT3G45300

482

590 // AT3G30775 // AT5G06580 // AT5G65110 // AT5G43430 // AT4G16760 // AT2G43400 // AT2G35690 // AT3G45300

930 // AT5G56470 // AT5G56490 // AT5G49970 // AT5G11540

930 // AT5G56470 // AT5G56490 // AT5G49970 // AT5G11540

775 // AT5G65110 // AT5G43430 // AT4G16760 // AT2G43400 // AT2G35690 // AT3G45300

415 // AT3G14130

060 // AT5G11540 // AT4G36400 // AT5G17770 // AT3G18030 // AT3G52880 // AT1G32300 // AT5G49740 // AT3G14150 // AT2G41680 // AT3G45300 // AT1G12130 // AT2

490 // AT5G11540

490 // AT5G11540

930 // AT5G56470 // AT5G56490 // AT5G49970 // AT5G11540

930 // AT5G56470 // AT5G56490 // AT5G49970 // AT5G11540

110 // AT4G16760 // AT2G35690

140 // AT2G02710 // AT5G57360

460 // AT3G17240 // AT1G15020 // AT4G16155 // AT2G01270 // AT1G48030 // AT3G16950

370 // AT2G41680 // AT4G32360 // AT2G44160 // AT5G08740 // AT1G04400 // AT5G43430 // AT3G02280 // AT1G04620 // AT5G14220 // AT3G16950 // AT3G18030 // AT5

110 // AT4G16760 // AT2G35690

740 // AT2G43020 // AT3G63440 // AT5G21482 // AT5G13700

760 // AT2G35690

760 // AT2G35690

760 // AT5G49740 // AT2G43400 // AT5G08740 // AT5G66190

960 // AT2G33230 // AT4G16760 // AT4G32360 // AT5G67030 // AT3G06690 // AT5G65110 // AT1G72970 // AT2G27150 // AT3G06810 // AT3G43600 // AT2G35690 // AT2

910 // AT5G38710 // AT5G43430 // AT4G34890 // AT2G43400 // AT3G45300

760 // AT2G35690

320 // AT4G34890 // AT5G21482 // AT2G19500 // AT5G13700 // AT3G52880 // AT5G65110 // AT3G45300 // AT1G50940 // AT3G10370 // AT2G41510 // AT4G34900 // AT3

150 // AT3G27820 // AT3G14415 // AT3G14130

482

482

400

400 // AT5G03630 // AT4G35460 // AT3G17240 // AT1G15020 // AT4G16155 // AT2G01270 // AT1G48030 // AT3G16950

110 // AT4G16760 // AT2G35690

570

570

760 // AT2G35690

360 // AT5G51060 // AT3G63440 // AT5G21482 // AT3G14415 // AT5G11540 // AT4G36400 // AT5G17770 // AT4G16310 // AT3G52880 // AT1G32300 // AT1G26380 // AT3

150

360 // AT1G12370 // AT5G51060 // AT1G09090 // AT4G34890 // AT3G24170 // AT3G14415 // AT1G77760 // AT1G20020 // AT1G73050 // AT4G08920 // AT1G26410 // AT3

030 // AT1G04400 // AT5G47910 // AT1G64060 // AT5G03630 // AT4G35460 // AT3G17240 // AT1G15020 // AT4G16155 // AT2G01270 // AT1G01580 // AT3G16950

210 // AT2G46740 // AT1G06820 // AT4G32360 // AT2G35660 // AT5G51060 // AT5G44360 // AT3G63440 // AT5G21482 // AT3G14415 // AT5G11540 // AT4G36400 // AT5

210 // AT2G46740 // AT1G06820 // AT4G32360 // AT2G35660 // AT5G51060 // AT5G44360 // AT3G63440 // AT5G21482 // AT3G14415 // AT5G11540 // AT4G36400 // AT5

210 // AT2G46740 // AT1G06820 // AT4G32360 // AT2G35660 // AT5G51060 // AT5G44360 // AT3G63440 // AT5G21482 // AT3G14415 // AT5G11540 // AT4G36400 // AT5

210 // AT2G46740 // AT1G06820 // AT4G32360 // AT2G35660 // AT5G51060 // AT5G44360 // AT3G63440 // AT5G21482 // AT3G14415 // AT5G11540 // AT4G36400 // AT5

740 // AT4G32360 // AT1G72970 // AT5G49970 // AT4G34890 // AT1G12140 // AT5G53460 // AT5G11540 // AT5G08530 // AT4G36400 // AT4G08920 // AT5G14760 // AT3

360 // AT1G72970 // AT1G12160 // AT1G12140 // AT5G53460 // AT5G11540 // AT1G50940 // AT4G36400 // AT2G06050 // AT2G41220 // AT1G32300 // AT1G77760 // AT5

360 // AT1G72970 // AT1G12160 // AT1G12140 // AT5G53460 // AT5G11540 // AT1G50940 // AT4G36400 // AT2G06050 // AT2G41220 // AT1G32300 // AT1G77760 // AT5

430 // AT2G27150 // AT1G04580 // AT2G41510 // AT1G04180 // AT4G32540 // AT4G29740 // AT2G33230 // AT5G56970 // AT4G13260 // AT5G11320 // AT1G48910 // AT3

970 // AT5G53460 // AT5G11540 // AT1G50940 // AT4G36400 // AT2G06050 // AT2G41220 // AT1G32300 // AT5G65110 // AT2G27150 // AT5G56490 // AT1G76680 // AT3

930 // AT1G12160 // AT2G35660 // AT4G37760 // AT1G12140 // AT5G08740 // AT5G53460 // AT5G49970 // AT4G01690 // AT4G15760 // AT5G14760 // AT5G24160 // AT3

210 // AT2G46740 // AT1G06820 // AT4G32360 // AT2G35660 // AT5G51060 // AT5G44360 // AT3G63440 // AT5G21482 // AT3G14415 // AT5G11540 // AT4G36400 // AT5

210 // AT2G46740 // AT1G06820 // AT4G32360 // AT2G35660 // AT5G51060 // AT5G44360 // AT3G63440 // AT5G21482 // AT3G14415 // AT5G11540 // AT4G36400 // AT5

210 // AT2G46740 // AT1G06820 // AT4G32360 // AT2G35660 // AT5G51060 // AT5G44360 // AT3G63440 // AT5G21482 // AT3G14415 // AT5G11540 // AT4G36400 // AT5

210 // AT2G46740 // AT1G06820 // AT4G32360 // AT2G35660 // AT5G51060 // AT5G44360 // AT3G63440 // AT5G21482 // AT3G14415 // AT5G11540 // AT4G36400 // AT5

740 // AT4G32360 // AT1G72970 // AT5G49970 // AT4G34890 // AT1G12140 // AT5G53460 // AT5G11540 // AT5G08530 // AT4G36400 // AT4G08920 // AT5G14760 // AT3

360 // AT1G72970 // AT1G12160 // AT1G12140 // AT5G53460 // AT5G11540 // AT1G50940 // AT4G36400 // AT2G06050 // AT2G41220 // AT1G32300 // AT1G77760 // AT5

360 // AT1G72970 // AT1G12160 // AT1G12140 // AT5G53460 // AT5G11540 // AT1G50940 // AT4G36400 // AT2G06050 // AT2G41220 // AT1G32300 // AT1G77760 // AT5

430 // AT2G27150 // AT1G04580 // AT2G41510 // AT1G04180 // AT4G32540 // AT4G29740 // AT2G33230 // AT5G56970 // AT4G13260 // AT5G11320 // AT1G48910 // AT3

970 // AT5G53460 // AT5G11540 // AT1G50940 // AT4G36400 // AT2G06050 // AT2G41220 // AT1G32300 // AT5G65110 // AT2G27150 // AT5G56490 // AT1G76680 // AT3

)30 // AT1G12160 // AT2G35660 // AT4G37760 // AT1G12140 // AT5G08740 // AT5G53460 // AT5G49970 // AT4G01690 // AT4G15760 // AT5G14760 // AT5G24160 // AT3

390 // AT4G28720 // AT5G11320 // AT1G04180 // AT2G27150 // AT3G43600 // AT5G25620 // AT4G32540 // AT5G20961

390 // AT1G21430 // AT2G27150 // AT1G04580 // AT2G41510 // AT1G04180 // AT4G32540 // AT4G29740 // AT2G33230 // AT5G56970 // AT4G13260 // AT5G11320 // AT1

)50 // AT2G41220 // AT1G32300 // AT2G27150 // AT5G56490 // AT1G76680 // AT1G04580 // AT2G46750 // AT3G30775 // AT3G59970 // AT5G04140 // AT5G56470 // AT4

390 // AT4G28720 // AT5G11320 // AT1G04180 // AT2G27150 // AT3G43600 // AT5G25620 // AT4G32540 // AT5G20961

390 // AT4G28720 // AT5G11320 // AT1G04180 // AT2G27150 // AT3G43600 // AT5G25620 // AT3G19820 // AT4G32540 // AT5G20961

)50 // AT2G41220 // AT1G32300 // AT2G27150 // AT5G56490 // AT1G76680 // AT1G04580 // AT2G46750 // AT2G46580 // AT3G30775 // AT3G59970 // AT5G04140 // AT5

)50 // AT2G41220 // AT1G32300 // AT2G27150 // AT5G56490 // AT1G76680 // AT1G04580 // AT2G46750 // AT3G30775 // AT3G59970 // AT5G04140 // AT5G56470 // AT4

482 // AT2G19500 // AT4G16155 // AT4G08920 // AT1G01580 // AT1G21430 // AT3G52880 // AT4G28720 // AT2G27150 // AT3G17240 // AT3G16950 // AT1G04580 // AT2

380 // AT1G04400 // AT5G47910 // AT1G64060 // AT3G14150 // AT3G27820 // AT4G34890 // AT4G35460 // AT3G14415 // AT3G14131

483

060 // AT3G63440 // AT5G21482 // AT3G14415 // AT5G11540 // AT4G36400 // AT5G17770 // AT4G16310 // AT3G52880 // AT1G32300 // AT1G20020 // AT5G49740 // AT3

775 // AT5G65110 // AT5G43430 // AT4G16760 // AT2G43400 // AT2G35690 // AT3G45301

483

590 // AT3G30775 // AT5G06580 // AT5G65110 // AT5G43430 // AT4G16760 // AT2G43400 // AT2G35690 // AT3G45301

930 // AT5G56470 // AT5G56490 // AT5G49970 // AT5G11540

930 // AT5G56470 // AT5G56490 // AT5G49970 // AT5G11540

'75 // AT5G65110 // AT5G43430 // AT4G16760 // AT2G43400 // AT2G35690 // AT3G45301

415 // AT3G14131

)60 // AT5G11540 // AT4G36400 // AT5G17770 // AT3G18030 // AT3G52880 // AT1G32300 // AT5G49740 // AT3G14150 // AT2G41680 // AT3G45300 // AT1G12130 // AT2

490 // AT5G11540

490 // AT5G11540

930 // AT5G56470 // AT5G56490 // AT5G49970 // AT5G11540

930 // AT5G56470 // AT5G56490 // AT5G49970 // AT5G11540

110 // AT4G16760 // AT2G35691

140 // AT2G02710 // AT5G57361

460 // AT3G17240 // AT1G15020 // AT4G16155 // AT2G01270 // AT1G48030 // AT3G16951

370 // AT2G41680 // AT4G32360 // AT2G44160 // AT5G08740 // AT1G04400 // AT5G43430 // AT3G02280 // AT1G04620 // AT5G14220 // AT3G16950 // AT3G18030 // AT5

110 // AT4G16760 // AT2G35691

740 // AT2G43020 // AT3G63440 // AT5G21482 // AT5G13701

760 // AT2G35690

760 // AT2G35690

760 // AT5G49740 // AT2G43400 // AT5G08740 // AT5G66191

960 // AT2G33230 // AT4G16760 // AT4G32360 // AT5G67030 // AT3G06690 // AT5G65110 // AT1G72970 // AT2G27150 // AT3G06810 // AT3G43600 // AT2G35690 // AT2

910 // AT5G38710 // AT5G43430 // AT4G34890 // AT2G43400 // AT3G45301

760 // AT2G35691

320 // AT4G34890 // AT5G21482 // AT2G19500 // AT5G13700 // AT3G52880 // AT5G65110 // AT3G45300 // AT1G50940 // AT3G10370 // AT2G41510 // AT4G34900 // AT3

150 // AT3G27820 // AT3G14415 // AT3G14131

483

483

401

400 // AT5G03630 // AT4G35460 // AT3G17240 // AT1G15020 // AT4G16155 // AT2G01270 // AT1G48030 // AT3G16951

110 // AT4G16760 // AT2G35691

570

570

760 // AT2G35691

360 // AT5G51060 // AT3G63440 // AT5G21482 // AT3G14415 // AT5G11540 // AT4G36400 // AT5G17770 // AT4G16310 // AT3G52880 // AT1G32300 // AT1G26380 // AT3

151

360 // AT1G12370 // AT5G51060 // AT1G09090 // AT4G34890 // AT3G24170 // AT3G14415 // AT1G77760 // AT1G20020 // AT1G73050 // AT4G08920 // AT1G26410 // AT3

030 // AT1G04400 // AT5G47910 // AT1G64060 // AT5G03630 // AT4G35460 // AT3G17240 // AT1G15020 // AT4G16155 // AT2G01270 // AT1G01580 // AT3G16951

210 // AT2G46740 // AT1G06820 // AT4G32360 // AT2G35660 // AT5G51060 // AT5G44360 // AT3G63440 // AT5G21482 // AT3G14415 // AT5G11540 // AT4G36400 // AT5

G05320 // AT4G15760 // AT4G16310 // AT3G52880 // AT1G32300 // AT5G44410 // AT1G20020 // AT5G07390 // AT5G49740 // AT3G14150 // AT5G67220 // AT5G44390 //

G05320 // AT4G15760 // AT4G16310 // AT3G52880 // AT1G32300 // AT5G44410 // AT1G20020 // AT5G07390 // AT5G49740 // AT3G14150 // AT5G67220 // AT5G44390 //

G05320 // AT4G15760 // AT4G16310 // AT3G52880 // AT1G32300 // AT5G44410 // AT1G20020 // AT5G07390 // AT5G49740 // AT3G14150 // AT5G67220 // AT5G44390 //

G05320 // AT4G15760 // AT4G16310 // AT3G52880 // AT1G32300 // AT5G44410 // AT3G15620 // AT5G07390 // AT5G49740 // AT3G14150 // AT5G67220 // AT5G44390 //

G18030 // AT2G41220 // AT1G32300 // AT1G77760 // AT3G59970 // AT5G65110 // AT2G27150 // AT2G46750 // AT5G56490 // AT1G76680 // AT1G62570 // AT5G23300 //

G65110 // AT2G27150 // AT5G56490 // AT1G76680 // AT1G62570 // AT3G45300 // AT3G16950 // AT1G04580 // AT5G38710 // AT2G46750 // AT1G12130 // AT3G30775 //

G65110 // AT2G27150 // AT5G56490 // AT1G76680 // AT1G62570 // AT3G45300 // AT3G16950 // AT1G04580 // AT5G38710 // AT2G46750 // AT1G12130 // AT3G30775 //

G43600 // AT3G19820 // AT1G75450 // AT4G28720 // AT5G20960

G45300 // AT3G16950 // AT1G04580 // AT2G46750 // AT5G38710 // AT3G30775 // AT3G59970 // AT1G65860 // AT5G43430 // AT5G04140 // AT5G56470 // AT4G16760 //

G18030 // AT2G41220 // AT1G32300 // AT5G24140 // AT2G27150 // AT2G29720 // AT5G56490 // AT1G76680 // AT1G62570 // AT2G41680 // AT3G16950 // AT1G04580 //

G48910 // AT3G43600 // AT3G19820 // AT1G75450 // AT4G28720 // AT5G20960

G16760 // AT5G20960 // AT2G33230 // AT3G48560 // AT5G67030 // AT5G38710 // AT3G43600 // AT2G35690 // AT1G76690 // AT1G48850

G56470 // AT4G16760 // AT5G11540 // AT5G20960 // AT2G33230 // AT3G48560 // AT5G67030 // AT5G08740 // AT5G38710 // AT3G43600 // AT2G35690 // AT1G76690 //

G16760 // AT5G20960 // AT2G33230 // AT3G48560 // AT5G67030 // AT5G38710 // AT3G43600 // AT2G35690 // AT1G76690 // AT1G48850

G17420 // AT2G41510 // AT1G04400 // AT5G47910 // AT1G04180 // AT5G03630 // AT2G01270 // AT4G32540 // AT4G29740 // AT1G15020 // AT4G35460 // AT2G33230 //

G14150 // AT2G41680 // AT3G45300 // AT5G24850 // AT1G12130 // AT2G46580 // AT4G30210 // AT4G32540 // AT1G06290 // AT2G47590 // AT2G33230 // AT5G67030 //

G46580 // AT1G06290 // AT4G30210 // AT2G33230 // AT5G67030 // AT1G64060 // AT2G02710 // AT3G06810 // AT2G43400 // AT1G62560 // AT1G48850 // AT2G46760 //

G49970

G06050 // AT3G16950

G30775 // AT5G06580 // AT4G29740 // AT2G43020 // AT5G57360 // AT4G16760 // AT2G35690 // AT1G06290 // AT3G59050 // AT1G68050 // AT5G56970 // AT2G18915 //

G15620 // AT5G49740 // AT3G14150 // AT2G41680 // AT3G45300 // AT5G24850 // AT1G12130 // AT2G46580 // AT4G30210 // AT4G32540 // AT1G06290 // AT1G07180 //

G18030 // AT5G44380 // AT4G37760 // AT1G26380 // AT3G15620 // AT5G07390 // AT3G14150 // AT1G76680 // AT2G06050 // AT5G24850 // AT1G48605 // AT4G20830 //

G05320 // AT4G15760 // AT4G16310 // AT3G52880 // AT1G32300 // AT5G44410 // AT1G20020 // AT5G07390 // AT5G49740 // AT3G14150 // AT5G67220 // AT5G44390 //

G05320 // AT4G15760 // AT4G16310 // AT3G52880 // AT1G32300 // AT5G44410 // AT1G20020 // AT5G07390 // AT5G49740 // AT3G14150 // AT5G67220 // AT5G44390 //

G05320 // AT4G15760 // AT4G16310 // AT3G52880 // AT1G32300 // AT5G44410 // AT1G20020 // AT5G07390 // AT5G49740 // AT3G14150 // AT5G67220 // AT5G44390 //

G05320 // AT4G15760 // AT4G16310 // AT3G52880 // AT1G32300 // AT5G44410 // AT3G15620 // AT5G07390 // AT5G49740 // AT3G14150 // AT5G67220 // AT5G44390 //

G18030 // AT2G41220 // AT1G32300 // AT1G77760 // AT3G59970 // AT5G65110 // AT2G27150 // AT2G46750 // AT5G56490 // AT1G76680 // AT1G62570 // AT5G23300 //

G65110 // AT2G27150 // AT5G56490 // AT1G76680 // AT1G62570 // AT3G45300 // AT3G16950 // AT1G04580 // AT5G38710 // AT2G46750 // AT1G12130 // AT3G30775 //

G65110 // AT2G27150 // AT5G56490 // AT1G76680 // AT1G62570 // AT3G45300 // AT3G16950 // AT1G04580 // AT5G38710 // AT2G46750 // AT1G12130 // AT3G30775 //

G43600 // AT3G19820 // AT1G75450 // AT4G28720 // AT5G20960

G45300 // AT3G16950 // AT1G04580 // AT2G46750 // AT5G38710 // AT3G30775 // AT3G59970 // AT1G65860 // AT5G43430 // AT5G04140 // AT5G56470 // AT4G16760 //

G18030 // AT2G41220 // AT1G32300 // AT5G24140 // AT2G27150 // AT2G29720 // AT5G56490 // AT1G76680 // AT1G62570 // AT2G41680 // AT3G16950 // AT1G04580 //

G05320 // AT4G15760 // AT4G16310 // AT3G52880 // AT1G32300 // AT5G44410 // AT1G20020 // AT5G07390 // AT5G49740 // AT3G14150 // AT5G67220 // AT5G44390 //

G05320 // AT4G15760 // AT4G16310 // AT3G52880 // AT1G32300 // AT5G44410 // AT1G20020 // AT5G07390 // AT5G49740 // AT3G14150 // AT5G67220 // AT5G44390 //

G05320 // AT4G15760 // AT4G16310 // AT3G52880 // AT1G32300 // AT5G44410 // AT1G20020 // AT5G07390 // AT5G49740 // AT3G14150 // AT5G67220 // AT5G44390 //

G05320 // AT4G15760 // AT4G16310 // AT3G52880 // AT1G32300 // AT5G44410 // AT3G15620 // AT5G07390 // AT5G49740 // AT3G14150 // AT5G67220 // AT5G44390 //

G18030 // AT2G41220 // AT1G32300 // AT1G77760 // AT3G59970 // AT5G65110 // AT2G27150 // AT2G46750 // AT5G56490 // AT1G76680 // AT1G62570 // AT5G23300 //

G65110 // AT2G27150 // AT5G56490 // AT1G76680 // AT1G62570 // AT3G45300 // AT3G16950 // AT1G04580 // AT5G38710 // AT2G46750 // AT1G12130 // AT3G30775 //

G65110 // AT2G27150 // AT5G56490 // AT1G76680 // AT1G62570 // AT3G45300 // AT3G16950 // AT1G04580 // AT5G38710 // AT2G46750 // AT1G12130 // AT3G30775 //

G43600 // AT3G19820 // AT1G75450 // AT4G28720 // AT5G20960

G45300 // AT3G16950 // AT1G04580 // AT2G46750 // AT5G38710 // AT3G30775 // AT3G59970 // AT1G65860 // AT5G43430 // AT5G04140 // AT5G56470 // AT4G16760 //

G18030 // AT2G41220 // AT1G32300 // AT5G24140 // AT2G27150 // AT2G29720 // AT5G56490 // AT1G76680 // AT1G62570 // AT2G41680 // AT3G16950 // AT1G04580 //

G48910 // AT3G43600 // AT3G19820 // AT1G75450 // AT4G28720 // AT5G20960

G16760 // AT5G20960 // AT2G33230 // AT3G48560 // AT5G67030 // AT5G38710 // AT3G43600 // AT2G35690 // AT1G76690 // AT1G48850

G56470 // AT4G16760 // AT5G11540 // AT5G20960 // AT2G33230 // AT3G48560 // AT5G67030 // AT5G08740 // AT5G38710 // AT3G43600 // AT2G35690 // AT1G76690 //

G16760 // AT5G20960 // AT2G33230 // AT3G48560 // AT5G67030 // AT5G38710 // AT3G43600 // AT2G35690 // AT1G76690 // AT1G48850

G17420 // AT2G41510 // AT1G04400 // AT5G47910 // AT1G04180 // AT5G03630 // AT2G01270 // AT4G32540 // AT4G29740 // AT1G15020 // AT4G35460 // AT2G33230 //

G14150 // AT2G41680 // AT3G45300 // AT5G24850 // AT1G12130 // AT2G46580 // AT4G30210 // AT4G32540 // AT1G06290 // AT2G47590 // AT2G33230 // AT5G67030 //

G46580 // AT1G06290 // AT4G30210 // AT2G33230 // AT5G67030 // AT1G64060 // AT2G02710 // AT3G06810 // AT2G43400 // AT1G62560 // AT1G48850 // AT2G46760 //

G49971

G06050 // AT3G16950

G30775 // AT5G06580 // AT4G29740 // AT2G43020 // AT5G57360 // AT4G16760 // AT2G35690 // AT1G06290 // AT3G59050 // AT1G68050 // AT5G56970 // AT2G18915 //

G15620 // AT5G49740 // AT3G14150 // AT2G41680 // AT3G45300 // AT5G24850 // AT1G12130 // AT2G46580 // AT4G30210 // AT4G32540 // AT1G06290 // AT1G07180 //

G18030 // AT5G44380 // AT4G37760 // AT1G26380 // AT3G15620 // AT5G07390 // AT3G14150 // AT1G76680 // AT2G06050 // AT5G24850 // AT1G48605 // AT4G20830 //

G05320 // AT4G15760 // AT4G16310 // AT3G52880 // AT1G32300 // AT5G44410 // AT1G20020 // AT5G07390 // AT5G49740 // AT3G14150 // AT5G67220 // AT5G44390 //

/ AT2G41680 // AT3G45300 // AT5G44400 // AT4G30210 // AT1G48910 // AT1G12130 // AT2G46580 // AT1G30740 // AT1G19250 // AT4G32540 // AT1G26390 // AT1G062

/ AT2G41680 // AT3G45300 // AT5G44400 // AT4G30210 // AT1G48910 // AT1G12130 // AT2G46580 // AT1G30740 // AT1G19250 // AT4G32540 // AT1G26390 // AT1G062

/ AT2G41680 // AT3G45300 // AT5G44400 // AT4G30210 // AT1G48910 // AT1G12130 // AT2G46580 // AT1G30740 // AT1G19250 // AT4G32540 // AT1G26390 // AT1G062

/ AT2G41680 // AT3G45300 // AT5G44400 // AT4G30210 // AT5G24850 // AT1G48910 // AT1G12130 // AT2G46580 // AT1G30740 // AT1G19250 // AT4G32540 // AT1G263

/ AT3G45300 // AT3G16950 // AT1G04580 // AT4G34900 // AT5G38710 // AT1G48605 // AT3G10370 // AT1G12130 // AT2G46580 // AT3G30775 // AT5G06580 // AT1G044

/ AT3G59970 // AT1G65860 // AT5G43430 // AT5G04140 // AT5G56470 // AT4G16760 // AT5G20960 // AT3G47930 // AT2G35690 // AT1G06290 // AT1G37130 // AT3G485

/ AT2G18450 // AT3G59970 // AT1G65860 // AT5G43430 // AT5G04140 // AT5G56470 // AT4G16760 // AT5G20960 // AT3G47930 // AT2G35690 // AT1G06290 // AT1G371

/ AT5G20960 // AT3G47930 // AT2G35690 // AT1G06290 // AT3G48560 // AT2G33230 // AT3G06690 // AT5G67030 // AT3G06810 // AT3G43600 // AT2G43400 // AT1G766

/ AT1G48605 // AT2G46750 // AT1G12130 // AT2G46580 // AT3G30775 // AT3G59970 // AT5G04140 // AT5G56470 // AT4G16760 // AT5G11540 // AT5G20960 // AT5G142

/ AT1G48850

/ AT5G56970 // AT4G13260 // AT1G48030 // AT5G11320 // AT1G64060 // AT1G48910 // AT3G43600 // AT3G19820 // AT1G75450 // AT5G43890 // AT5G20960

/ AT1G72280 // AT1G64060 // AT2G02710 // AT1G48910 // AT3G06810 // AT2G43400 // AT1G62560 // AT1G48850 // AT2G46760 // AT4G24520 // AT4G18360 // AT3G485

/ AT4G24520 // AT2G34790 // AT4G18360 // AT3G06690 // AT5G08530 // AT4G08920 // AT2G41220 // AT3G24170 // AT5G65110 // AT2G27150 // AT2G46750 // AT4G016

/ AT1G65840 // AT5G38710 // AT5G43430 // AT2G43400 // AT1G75450

/ AT2G47590 // AT2G33230 // AT5G67030 // AT1G72280 // AT1G64060 // AT2G02710 // AT1G48910 // AT3G06810 // AT2G43400 // AT1G62560 // AT1G48850 // AT2G467

/ AT1G12200 // AT3G30775 // AT1G04400 // AT5G47910 // AT5G03630 // AT4G16760 // AT3G09940 // AT5G44440 // AT5G66190 // AT2G20360 // AT3G14420 // AT1G192

/ AT2G41680 // AT3G45300 // AT5G44400 // AT4G30210 // AT1G48910 // AT1G12130 // AT2G46580 // AT1G30740 // AT1G19250 // AT4G32540 // AT1G26390 // AT1G062

/ AT2G41680 // AT3G45300 // AT5G44400 // AT4G30210 // AT1G48910 // AT1G12130 // AT2G46580 // AT1G30740 // AT1G19250 // AT4G32540 // AT1G26390 // AT1G062

/ AT2G41680 // AT3G45300 // AT5G44400 // AT4G30210 // AT1G48910 // AT1G12130 // AT2G46580 // AT1G30740 // AT1G19250 // AT4G32540 // AT1G26390 // AT1G062

/ AT2G41680 // AT3G45300 // AT5G44400 // AT4G30210 // AT5G24850 // AT1G48910 // AT1G12130 // AT2G46580 // AT1G30740 // AT1G19250 // AT4G32540 // AT1G263

/ AT3G45300 // AT3G16950 // AT1G04580 // AT4G34900 // AT5G38710 // AT1G48605 // AT3G10370 // AT1G12130 // AT2G46580 // AT3G30775 // AT5G06580 // AT1G044

/ AT3G59970 // AT1G65860 // AT5G43430 // AT5G04140 // AT5G56470 // AT4G16760 // AT5G20960 // AT3G47930 // AT2G35690 // AT1G06290 // AT1G37130 // AT3G485

/ AT2G18450 // AT3G59970 // AT1G65860 // AT5G43430 // AT5G04140 // AT5G56470 // AT4G16760 // AT5G20960 // AT3G47930 // AT2G35690 // AT1G06290 // AT1G371

/ AT5G20960 // AT3G47930 // AT2G35690 // AT1G06290 // AT3G48560 // AT2G33230 // AT3G06690 // AT5G67030 // AT3G06810 // AT3G43600 // AT2G43400 // AT1G766

/ AT1G48605 // AT2G46750 // AT1G12130 // AT2G46580 // AT3G30775 // AT3G59970 // AT5G04140 // AT5G56470 // AT4G16760 // AT5G11540 // AT5G20960 // AT5G142

/ AT2G41680 // AT3G45300 // AT5G44400 // AT4G30210 // AT1G48910 // AT1G12130 // AT2G46580 // AT1G30740 // AT1G19250 // AT4G32540 // AT1G26390 // AT1G062

/ AT2G41680 // AT3G45300 // AT5G44400 // AT4G30210 // AT1G48910 // AT1G12130 // AT2G46580 // AT1G30740 // AT1G19250 // AT4G32540 // AT1G26390 // AT1G062

/ AT2G41680 // AT3G45300 // AT5G44400 // AT4G30210 // AT1G48910 // AT1G12130 // AT2G46580 // AT1G30740 // AT1G19250 // AT4G32540 // AT1G26390 // AT1G062

/ AT2G41680 // AT3G45300 // AT5G44400 // AT4G30210 // AT5G24850 // AT1G48910 // AT1G12130 // AT2G46580 // AT1G30740 // AT1G19250 // AT4G32540 // AT1G263

/ AT3G45300 // AT3G16950 // AT1G04580 // AT4G34900 // AT5G38710 // AT1G48605 // AT3G10370 // AT1G12130 // AT2G46580 // AT3G30775 // AT5G06580 // AT1G044

/ AT3G59970 // AT1G65860 // AT5G43430 // AT5G04140 // AT5G56470 // AT4G16760 // AT5G20960 // AT3G47930 // AT2G35690 // AT1G06290 // AT1G37130 // AT3G485

/ AT2G18450 // AT3G59970 // AT1G65860 // AT5G43430 // AT5G04140 // AT5G56470 // AT4G16760 // AT5G20960 // AT3G47930 // AT2G35690 // AT1G06290 // AT1G371

/ AT5G20960 // AT3G47930 // AT2G35690 // AT1G06290 // AT3G48560 // AT2G33230 // AT3G06690 // AT5G67030 // AT3G06810 // AT3G43600 // AT2G43400 // AT1G766

/ AT1G48605 // AT2G46750 // AT1G12130 // AT2G46580 // AT3G30775 // AT3G59970 // AT5G04140 // AT5G56470 // AT4G16760 // AT5G11540 // AT5G20960 // AT5G142

/ AT1G48850

/ AT5G56970 // AT4G13260 // AT1G48030 // AT5G11320 // AT1G64060 // AT1G48910 // AT3G43600 // AT3G19820 // AT1G75450 // AT5G43890 // AT5G20960

/ AT1G72280 // AT1G64060 // AT2G02710 // AT1G48910 // AT3G06810 // AT2G43400 // AT1G62560 // AT1G48850 // AT2G46760 // AT4G24520 // AT4G18360 // AT3G485

/ AT4G24520 // AT2G34790 // AT4G18360 // AT3G06690 // AT5G08530 // AT4G08920 // AT2G41220 // AT3G24170 // AT5G65110 // AT2G27150 // AT2G46750 // AT4G016

/ AT1G65840 // AT5G38710 // AT5G43430 // AT2G43400 // AT1G75450

/ AT2G47590 // AT2G33230 // AT5G67030 // AT1G72280 // AT1G64060 // AT2G02710 // AT1G48910 // AT3G06810 // AT2G43400 // AT1G62560 // AT1G48850 // AT2G467

/ AT1G12200 // AT3G30775 // AT1G04400 // AT5G47910 // AT5G03630 // AT4G16760 // AT3G09940 // AT5G44440 // AT5G66190 // AT2G20360 // AT3G14420 // AT1G192

/ AT2G41680 // AT3G45300 // AT5G44400 // AT4G30210 // AT1G48910 // AT1G12130 // AT2G46580 // AT1G30740 // AT1G19250 // AT4G32540 // AT1G26390 // AT1G062

290 // AT1G12570 // AT5G58800 // AT3G27890 // AT5G67030 // AT1G34575 // AT1G72280 // AT1G64060 // AT3G02280 // AT3G06810 // AT2G43400 // AT4G20840 // AT2

290 // AT1G12570 // AT5G58800 // AT3G27890 // AT5G67030 // AT1G34575 // AT1G72280 // AT1G64060 // AT3G02280 // AT3G06810 // AT2G43400 // AT4G20840 // AT2

290 // AT1G12570 // AT5G58800 // AT3G27890 // AT5G67030 // AT1G34575 // AT1G72280 // AT1G64060 // AT2G02710 // AT1G75200 // AT3G06810 // AT2G43400 // AT4

390 // AT1G06290 // AT1G12570 // AT2G47590 // AT5G58800 // AT3G27890 // AT5G67030 // AT1G34575 // AT1G72280 // AT1G64060 // AT2G02710 // AT1G75200 // AT3

400 // AT3G23410 // AT1G65860 // AT5G43430 // AT5G04140 // AT5G56470 // AT4G16760 // AT5G20960 // AT3G47930 // AT2G35690 // AT1G12160 // AT1G06290 // AT1

560 // AT2G33230 // AT3G06690 // AT5G67030 // AT3G06810 // AT3G43600 // AT2G43400 // AT1G62560 // AT1G76690 // AT1G48850

130 // AT3G48560 // AT2G33230 // AT3G06690 // AT5G67030 // AT5G66760 // AT3G06810 // AT3G43600 // AT2G43400 // AT1G62560 // AT1G76690 // AT1G48850

590 // AT1G48850

20 // AT3G19820 // AT5G24150 // AT5G23300 // AT1G65860 // AT2G33230 // AT3G48560 // AT5G67030 // AT1G73050 // AT2G06050 // AT2G22830 // AT5G38710 // AT3

;60 // AT3G27820 // AT1G30510 // AT5G08530 // AT1G62830 // AT4G08920 // AT2G41220 // AT3G02280 // AT2G38960 // AT5G65110 // AT2G27150 // AT2G46750 // AT1(

590 // AT1G62570 // AT3G16950 // AT1G48605 // AT5G06580 // AT1G04400 // AT5G47910 // AT4G16155 // AT1G72970 // AT1G62580 // AT5G58140 // AT2G35690 // AT1

'60 // AT4G24520 // AT2G34790 // AT4G18360 // AT3G48560 // AT3G27820 // AT1G30510 // AT5G08530 // AT1G62830 // AT4G08920 // AT2G41220 // AT3G02280 // AT2[illegible]

:50 // AT1G15020 // AT1G63940 // AT1G37130 // AT2G47590 // AT3G27890 // AT3G48560 // AT5G67030 // AT1G58440 // AT1G19230 // AT1G64060 // AT5G38710 // AT2[illegible]

90 // AT1G12570 // AT5G58800 // AT3G27890 // AT5G67030 // AT1G34575 // AT1G72280 // AT1G64060 // AT3G02280 // AT3G06810 // AT2G43400 // AT4G20840 // AT2

90 // AT1G12570 // AT5G58800 // AT3G27890 // AT5G67030 // AT1G34575 // AT1G72280 // AT1G64060 // AT3G02280 // AT3G06810 // AT2G43400 // AT4G20840 // AT2

90 // AT1G12570 // AT5G58800 // AT3G27890 // AT5G67030 // AT1G34575 // AT1G72280 // AT1G64060 // AT2G02710 // AT1G75200 // AT3G06810 // AT2G43400 // AT4

90 // AT1G06290 // AT1G12570 // AT2G47590 // AT5G58800 // AT3G27890 // AT5G67030 // AT1G34575 // AT1G72280 // AT1G64060 // AT2G02710 // AT1G75200 // AT3

00 // AT3G23410 // AT1G65860 // AT5G43430 // AT5G04140 // AT5G56470 // AT4G16760 // AT5G20960 // AT3G47930 // AT2G35690 // AT1G12160 // AT1G06290 // AT1

60 // AT2G33230 // AT3G06690 // AT5G67030 // AT3G06810 // AT3G43600 // AT2G43400 // AT1G62560 // AT1G76690 // AT1G48850

30 // AT3G48560 // AT2G33230 // AT3G06690 // AT5G67030 // AT5G66760 // AT3G06810 // AT3G43600 // AT2G43400 // AT1G62560 // AT1G76690 // AT1G48850

90 // AT1G48850

20 // AT3G19820 // AT5G24150 // AT5G23300 // AT1G65860 // AT2G33230 // AT3G48560 // AT5G67030 // AT1G73050 // AT2G06050 // AT2G22830 // AT5G38710 // AT3

90 // AT1G12570 // AT5G58800 // AT3G27890 // AT5G67030 // AT1G34575 // AT1G72280 // AT1G64060 // AT3G02280 // AT3G06810 // AT2G43400 // AT4G20840 // AT2

90 // AT1G12570 // AT5G58800 // AT3G27890 // AT5G67030 // AT1G34575 // AT1G72280 // AT1G64060 // AT3G02280 // AT3G06810 // AT2G43400 // AT4G20840 // AT2

90 // AT1G12570 // AT5G58800 // AT3G27890 // AT5G67030 // AT1G34575 // AT1G72280 // AT1G64060 // AT2G02710 // AT1G75200 // AT3G06810 // AT2G43400 // AT4

90 // AT1G06290 // AT1G12570 // AT2G47590 // AT5G58800 // AT3G27890 // AT5G67030 // AT1G34575 // AT1G72280 // AT1G64060 // AT2G02710 // AT1G75200 // AT3

00 // AT3G23410 // AT1G65860 // AT5G43430 // AT5G04140 // AT5G56470 // AT4G16760 // AT5G20960 // AT3G47930 // AT2G35690 // AT1G12160 // AT1G06290 // AT1

60 // AT2G33230 // AT3G06690 // AT5G67030 // AT3G06810 // AT3G43600 // AT2G43400 // AT1G62560 // AT1G76690 // AT1G48850

30 // AT3G48560 // AT2G33230 // AT3G06690 // AT5G67030 // AT5G66760 // AT3G06810 // AT3G43600 // AT2G43400 // AT1G62560 // AT1G76690 // AT1G48850

90 // AT1G48850

20 // AT3G19820 // AT5G24150 // AT5G23300 // AT1G65860 // AT2G33230 // AT3G48560 // AT5G67030 // AT1G73050 // AT2G06050 // AT2G22830 // AT5G38710 // AT3

[illegible]60 // AT3G27820 // AT1G30510 // AT5G08530 // AT1G62830 // AT4G08920 // AT2G41220 // AT3G02280 // AT2G38960 // AT5G65110 // AT2G27150 // AT2G46750 // AT1[illegible]

590 // AT1G62570 // AT3G16950 // AT1G48605 // AT5G06580 // AT1G04400 // AT5G47910 // AT4G16155 // AT1G72970 // AT1G62580 // AT5G58140 // AT2G35690 // AT1

'60 // AT4G24520 // AT2G34790 // AT4G18360 // AT3G48560 // AT3G27820 // AT1G30510 // AT5G08530 // AT1G62830 // AT4G08920 // AT2G41220 // AT3G02280 // AT2[illegible]

:50 // AT1G15020 // AT1G63940 // AT1G37130 // AT2G47590 // AT3G27890 // AT3G48560 // AT5G67030 // AT1G58440 // AT1G19230 // AT1G64060 // AT5G38710 // AT2[illegible]

[illegible]90 // AT1G12570 // AT5G58800 // AT3G27890 // AT5G67030 // AT1G34575 // AT1G72280 // AT1G64060 // AT3G02280 // AT3G06810 // AT2G43400 // AT4G20840 // AT2[illegible]

G33230 // AT2G46760 // AT4G28570 // AT4G24520 // AT1G01980 // AT2G34790 // AT5G60010 // AT4G18360 // AT3G27820 // AT3G45810 // AT5G08530 // AT5G23980 //

G33230 // AT1G48850 // AT2G46760 // AT4G28570 // AT4G24520 // AT1G01980 // AT2G34790 // AT5G60010 // AT4G18360 // AT3G48560 // AT3G27820 // AT3G45810 //

G20840 // AT2G33230 // AT1G48850 // AT2G46760 // AT4G28570 // AT4G24520 // AT1G01980 // AT2G34790 // AT5G60010 // AT4G18360 // AT3G48560 // AT3G27820 //

G06810 // AT2G43400 // AT4G20840 // AT2G33230 // AT1G48850 // AT2G46760 // AT4G28570 // AT4G24520 // AT1G01980 // AT2G34790 // AT5G60010 // AT4G18360 //

G37130 // AT3G48560 // AT2G33230 // AT3G06690 // AT5G67030 // AT5G08740 // AT1G50940 // AT5G66760 // AT3G06810 // AT3G43600 // AT2G43400 // AT1G62560 //

G43600 // AT2G35690 // AT1G62560 // AT1G76690 // AT1G58440 // AT1G48850

G04620 // AT4G01690 // AT1G62570 // AT3G16950 // AT1G75200 // AT1G48605 // AT5G06580 // AT1G04400 // AT5G47910 // AT2G43020 // AT1G75450 // AT3G59050 //

G73050 // AT2G44160 // AT1G06310 // AT2G18450 // AT5G63910 // AT4G34890 // AT1G12140 // AT5G49970 // AT5G14760 // AT5G56490 // AT1G76680 // AT1G04580 //

G38960 // AT5G65110 // AT2G27150 // AT2G46750 // AT1G04620 // AT4G01690 // AT1G62570 // AT3G16950 // AT1G75200 // AT1G48605 // AT5G06580 // AT1G04400 //

G35690 // AT3G14130 // AT1G76690 // AT2G33230 // AT4G35460

G33230 // AT2G46760 // AT4G28570 // AT4G24520 // AT1G01980 // AT2G34790 // AT5G60010 // AT4G18360 // AT3G27820 // AT3G45810 // AT5G08530 // AT5G23980 //

G33230 // AT1G48850 // AT2G46760 // AT4G28570 // AT4G24520 // AT1G01980 // AT2G34790 // AT5G60010 // AT4G18360 // AT3G48560 // AT3G27820 // AT3G45810 //

G20840 // AT2G33230 // AT1G48850 // AT2G46760 // AT4G28570 // AT4G24520 // AT1G01980 // AT2G34790 // AT5G60010 // AT4G18360 // AT3G48560 // AT3G27820 //

G06810 // AT2G43400 // AT4G20840 // AT2G33230 // AT1G48850 // AT2G46760 // AT4G28570 // AT4G24520 // AT1G01980 // AT2G34790 // AT5G60010 // AT4G18360 //

G37130 // AT3G48560 // AT2G33230 // AT3G06690 // AT5G67030 // AT5G08740 // AT1G50940 // AT5G66760 // AT3G06810 // AT3G43600 // AT2G43400 // AT1G62560 //

G43600 // AT2G35690 // AT1G62560 // AT1G76690 // AT1G58440 // AT1G48850

G33230 // AT2G46760 // AT4G28570 // AT4G24520 // AT1G01980 // AT2G34790 // AT5G60010 // AT4G18360 // AT3G27820 // AT3G45810 // AT5G08530 // AT5G23980 //

G33230 // AT1G48850 // AT2G46760 // AT4G28570 // AT4G24520 // AT1G01980 // AT2G34790 // AT5G60010 // AT4G18360 // AT3G48560 // AT3G27820 // AT3G45810 //

G20840 // AT2G33230 // AT1G48850 // AT2G46760 // AT4G28570 // AT4G24520 // AT1G01980 // AT2G34790 // AT5G60010 // AT4G18360 // AT3G48560 // AT3G27820 //

G06810 // AT2G43400 // AT4G20840 // AT2G33230 // AT1G48850 // AT2G46760 // AT4G28570 // AT4G24520 // AT1G01980 // AT2G34790 // AT5G60010 // AT4G18360 //

G37130 // AT3G48560 // AT2G33230 // AT3G06690 // AT5G67030 // AT5G08740 // AT1G50940 // AT5G66760 // AT3G06810 // AT3G43600 // AT2G43400 // AT1G62560 //

G43600 // AT2G35690 // AT1G62560 // AT1G76690 // AT1G58440 // AT1G48850

G04620 // AT4G01690 // AT1G62570 // AT3G16950 // AT1G75200 // AT1G48605 // AT5G06580 // AT1G04400 // AT5G47910 // AT2G43020 // AT1G75450 // AT3G59050 //

G73050 // AT2G44160 // AT1G06310 // AT2G18450 // AT5G63910 // AT4G34890 // AT1G12140 // AT5G49970 // AT5G14760 // AT5G56490 // AT1G76680 // AT1G04580 //

G38960 // AT5G65110 // AT2G27150 // AT2G46750 // AT1G04620 // AT4G01690 // AT1G62570 // AT3G16950 // AT1G75200 // AT1G48605 // AT5G06580 // AT1G04400 //

G35690 // AT3G14130 // AT1G76690 // AT2G33230 // AT4G35460

G33230 // AT2G46760 // AT4G28570 // AT4G24520 // AT1G01980 // AT2G34790 // AT5G60010 // AT4G18360 // AT3G27820 // AT3G45810 // AT5G08530 // AT5G23980 //

AT1G62830 // AT4G08920 // AT4G29720 // AT4G13260 // AT4G36750 // AT2G38960 // AT1G24340 // AT5G65110 // AT2G27150 // AT2G46750 // AT1G04620 // AT1G039

AT5G08530 // AT5G23980 // AT1G62830 // AT4G08920 // AT4G29720 // AT4G13260 // AT4G36750 // AT2G38960 // AT1G24340 // AT5G65110 // AT3G18030 // AT2G271

AT3G45810 // AT1G30510 // AT5G08530 // AT5G23980 // AT1G62830 // AT4G08920 // AT4G29720 // AT4G13260 // AT4G36750 // AT2G38960 // AT1G24340 // AT5G651

AT3G48560 // AT3G27820 // AT3G45810 // AT1G30510 // AT5G08530 // AT5G23980 // AT1G62830 // AT4G08920 // AT4G29720 // AT4G13260 // AT4G36750 // AT2G389

AT1G76690 // AT2G06050 // AT1G48850

' AT1G76690 // AT1G72970 // AT5G11320 // AT5G58140 // AT2G35690 // AT1G73050 // AT2G44160 // AT1G06310 // AT2G18450 // AT5G63910 // AT3G24170 // AT1G121

' AT3G59970 // AT5G43430 // AT5G04140 // AT5G03630 // AT4G16760 // AT1G19250 // AT1G15020 // AT1G65860 // AT3G48560 // AT5G66760 // AT3G19820 // AT1G766

' AT5G47910 // AT2G43020 // AT4G16155 // AT1G75450 // AT3G59050 // AT1G76690 // AT1G72970 // AT1G62580 // AT5G11320 // AT5G58140 // AT2G35690 // AT1G730

' AT1G62830 // AT4G08920 // AT4G29720 // AT4G13260 // AT4G36750 // AT2G38960 // AT1G24340 // AT5G65110 // AT2G27150 // AT2G46750 // AT1G04620 // AT1G039

' AT5G08530 // AT5G23980 // AT1G62830 // AT4G08920 // AT4G29720 // AT4G13260 // AT4G36750 // AT2G38960 // AT1G24340 // AT5G65110 // AT3G18030 // AT2G271

' AT3G45810 // AT1G30510 // AT5G08530 // AT5G23980 // AT1G62830 // AT4G08920 // AT4G29720 // AT4G13260 // AT4G36750 // AT2G38960 // AT1G24340 // AT5G651

' AT3G48560 // AT3G27820 // AT3G45810 // AT1G30510 // AT5G08530 // AT5G23980 // AT1G62830 // AT4G08920 // AT4G29720 // AT4G13260 // AT4G36750 // AT2G389

' AT1G76690 // AT2G06050 // AT1G48850

' AT1G62830 // AT4G08920 // AT4G29720 // AT4G13260 // AT4G36750 // AT2G38960 // AT1G24340 // AT5G65110 // AT2G27150 // AT2G46750 // AT1G04620 // AT1G039

' AT5G08530 // AT5G23980 // AT1G62830 // AT4G08920 // AT4G29720 // AT4G13260 // AT4G36750 // AT2G38960 // AT1G24340 // AT5G65110 // AT3G18030 // AT2G271

' AT3G45810 // AT1G30510 // AT5G08530 // AT5G23980 // AT1G62830 // AT4G08920 // AT4G29720 // AT4G13260 // AT4G36750 // AT2G38960 // AT1G24340 // AT5G651

' AT3G48560 // AT3G27820 // AT3G45810 // AT1G30510 // AT5G08530 // AT5G23980 // AT1G62830 // AT4G08920 // AT4G29720 // AT4G13260 // AT4G36750 // AT2G389

' AT1G76690 // AT2G06050 // AT1G48850

' AT1G76690 // AT1G72970 // AT5G11320 // AT5G58140 // AT2G35690 // AT1G73050 // AT2G44160 // AT1G06310 // AT2G18450 // AT5G63910 // AT3G24170 // AT1G121

' AT3G59970 // AT5G43430 // AT5G04140 // AT5G03630 // AT4G16760 // AT1G19250 // AT1G15020 // AT1G65860 // AT3G48560 // AT5G66760 // AT3G19820 // AT1G766

' AT5G47910 // AT2G43020 // AT4G16155 // AT1G75450 // AT3G59050 // AT1G76690 // AT1G72970 // AT1G62580 // AT5G11320 // AT5G58140 // AT2G35690 // AT1G730

' AT1G62830 // AT4G08920 // AT4G29720 // AT4G13260 // AT4G36750 // AT2G38960 // AT1G24340 // AT5G65110 // AT2G27150 // AT2G46750 // AT1G04620 // AT1G039

90 // AT1G62570 // AT4G388

50 // AT2G46750 // AT1G046

10 // AT3G18030 // AT2G271

60 // AT1G24340 // AT5G651

40 // AT5G49970 // AT2G195

90 // AT1G48030 // AT1G625

50 // AT2G44160 // AT1G063

90 // AT1G62570 // AT4G388

50 // AT2G46750 // AT1G046

10 // AT3G18030 // AT2G271

60 // AT1G24340 // AT5G651

90 // AT1G62570 // AT4G388

50 // AT2G46750 // AT1G046

10 // AT3G18030 // AT2G271

60 // AT1G24340 // AT5G651

40 // AT5G49970 // AT2G195

90 // AT1G48030 // AT1G625

50 // AT2G44160 // AT1G063

90 // AT1G62570 // AT4G388
