# Supplementary material for: The Flavoproteome of the Model Plant Arabidopsis thaliana
Source: Int J Mol Sci. 2020 Jul 28;21(15):5371. doi: 10.3390/ijms21155371 (PMC7432721; doi:10.3390/ijms21155371)
Supplement: Supplementary file 1 [file ijms-21-05371-s001.zip › Table S1 21.07.2020.pdf]

Table S1. Flavoproteins of Arabidopsis thaliana. The list has been collected by searching the TAIR, Gene Ontology, UniProt and PDB database for flavoproteins associated terms.

Gene Locus	Protein Name	Protein Family	E.C.	Localization (Literature)	Reference (Localization)	Localization (predicted)	Cofactor	Evidence Code	Reference (Flavin binding)
AT1G04400	Cryptochrome-2	DNA photolyase class-1 family	N/A	Nucleus ; Cytoplasm	(8, 9)		FAD, MTHF	1	(7)
AT1G04620	7-hydroxymethyl chlorophyll a reductase, chloroplastic	FrhB family	1.17.7.2	Plastid, chloroplast	(13)		FAD, [4Fe-4S]	1	(12)
AT1G06820	Prolycopene isomerase, chloroplastic	Carotenoid/retinoid oxidoreductase family, CrtISO subfamily	5.2.1.13			chloroplast membrane, chloroplast	FAD, NAD, NADP	1	(16)
AT1G12370	Deoxyribodipyrimidine photo-lyase	DNA photolyase class-2 family	4.1.99.3	Nucleus	(22)		FAD	1	(21)
AT1G15020	Sulfhydryl oxidase 1		1.8.3.2	cell wall	(24)		FAD	1	(23)
AT1G37130	Nitrate reductase [NADH] 2	Nitrate reductase family	1.7.1.1			vacuole, mitochondrion, nucleus, plasma membrane, cytosol	FAD, heme, Mo-molybdopterin	1	(30)
AT1G49880	FAD-linked sulfhydryl oxidase ERV1		1.8.3.2	Mitochondrion	(36)		FAD	1	(35)
AT1G65840	Polyamine oxidase 4	Flavin monoamine oxidase family	1.5.3.16	Peroxisome	(46)		FAD	1	(45)
AT1G72280	Endoplasmic reticulum oxidoreductin-1	EROs family	1.8.4.-	Endoplasmic reticulum membrane; Peripheral membrane protein ; Lumenal side	(51)		FAD	1	(50)
AT1G76680	12-oxophytodienoate reductase 1	NADH:flavin oxidoreductase/NADH oxidase family	1.3.1.42	Cytoplasm	(56)		FMN	1	(55)
AT2G06050	12-oxophytodienoate reductase 3	NADH:flavin oxidoreductase/NADH oxidase family	1.3.1.42	Peroxisome	(56)		FMN	1	(59)
AT2G24580	Sarcosine oxidase	MSOX/MTOX family	1.5.3.1			cytoplasm	FAD	1	(66)
AT2G34790	Berberine bridge enzyme-like 15	Oxygen-dependent FAD-linked oxidoreductase family	1.1.1.194; 1.1.1.195	Secreted, cell wall	(3)		FAD	1	(3)
AT2G38960	Endoplasmic reticulum oxidoreductin-2	EROs family	1.8.4.-	Endoplasmic reticulum membrane ; Peripheral membrane protein ; Lumenal side	(51)		FAD	1	(50)
AT2G43020	Polyamine oxidase 2	Flavin monoamine oxidase family	1.5.3.-	Peroxisome	(46)		FAD	1	(45)
AT3G10370	Glycerol-3-phosphate dehydrogenase SDP6, mitochondrial	FAD-dependent glycerol-3-phosphate dehydrogenase family	1.1.5.3	Mitochondrion inner membrane	(75)		FAD	1	(75)
AT3G14415	(S)-2-hydroxy-acid oxidase	FMN-dependent alpha-hydroxy acid dehydrogenase family	1.1.3.15			vacuole, chloroplast stroma, apoplast, nucleus, peroxisome, cytosol, chloroplast	FMN	1	(77)
AT3G14420	(S)-2-hydroxy-acid oxidase	FMN-dependent alpha-hydroxy acid dehydrogenase family	1.1.3.15			cytosolic ribosome, chloroplast stroma, apoplast, nucleus, peroxisome, cytoplasm, cytosol, chloroplast, plasmodesma	FMN	1	(77)
AT3G15620	(6-4)DNA photolyase	DNA photolyase class-1 family	4.1.99.13			chloroplast	FAD	1	(78)
AT3G18030	Phosphopantothenoylcysteine decarboxylase	HFCD (homooligomeric flavin containing Cys decarboxylase) superfamily	4.1.1.36			cytoplasm, cytosol	FMN	1	(80)
AT3G27890	NADPH:quinone oxidoreductase	SsuE family	1.6.5.2	Cell membrane	(86)		FMN	1	(85)
AT3G45780	Phototropin-1	Protein kinase superfamily, AGC Ser/Thr protein kinase family	2.7.11.1			Cell membrane; Peripheral membrane protein, Cytoplasm	FMN	1	(92)
AT3G47930	L-galactono-1,4-lactone dehydrogenase, mitochondrial		1.3.2.3	Mitochondrion membrane ; Single-pass membrane protein	(93)		FAD	1	(93)
AT3G48560	Acetolactate synthase, chloroplastic	TPP enzyme family	2.2.1.6	Plastid, chloroplast	(95)		FAD, Mg^{2+}, thiamine diphosphate	1	(94)
AT3G51840	Acyl-coenzyme A oxidase 4, peroxisomal	Acyl-CoA dehydrogenase family	1.3.3.6			Peroxisome	FAD	1	(96)
AT3G56840	L-2-hydroxyglutarate dehydrogenase, mitochondrial	L2HGDH family	1.1.99.2	Mitochondrion	(99)		FAD	1	(98)
AT3G59970	Methylenetetrahydrofolate reductase 1	Methylenetetrahydrofolate reductase family	1.5.1.20			cytosol	FAD	1	(100)
AT4G08920	Cryptochrome-1	DNA photolyase class-1 family	N/A	Cytoplasm; Nucleus; PML body. Present in nuclear bodies	(103, 104)		FAD, MTHF	1	(102)
AT4G16760	Peroxisomal acyl-coenzyme A oxidase 1	Acyl-CoA oxidase family	1.3.3.6	Peroxisome	(109)		FAD	1	(108)
AT4G18360	Peroxisomal (S)-2-hydroxy-acid oxidase GLO5	FMN-dependent alpha-hydroxy acid dehydrogenase family	1.1.3.15			peroxisome	FMN	1	(77)
AT4G29720	Polyamine oxidase 5	Flavin monoamine oxidase family	1.5.3.-	Cytoplasm	(112)		FAD	1	(112)
AT4G30210	NADPH--cytochrome P450 reductase 2	NADPH--cytochrome P450 reductase family; Flavodoxin family; Flavoprotein pyridine nucleotide cytochrome reductase family	1.6.2.4			endoplasmic reticulum membrane, endoplasmic reticulum, plasma membrane, cytosol, chloroplast, integral component of membrane	FAD, FMN	1	(113)
AT4G32360	NADPH:adrenodoxin oxidoreductase, mitochondrial	Ferredoxin--NADP reductase type 1 family	1.18.1.6	Mitochondrion	(115)		FAD	1	(114)
AT4G34890	Xanthine dehydrogenase 1	Xanthine dehydrogenase family	1.17.1.4			plasma membrane, cytosol	FAD, [2Fe-2S], Mo-molybdopterin	1	(116)
AT4G35460	Thioredoxin reductase 1, mitochondrial	Class-II pyridine nucleotide-disulfide oxidoreductase family	1.8.1.9	Cytoplasm ; Mitochondrion	(60)		FAD	1	(117)
AT4G36400	D-2-hydroxyglutarate dehydrogenase, mitochondrial	FAD-binding oxidoreductase/transferase type 4 family	1.1.99.39	Mitochondrion	(118)		FAD	1	(118)
AT5G06580	D-lactate dehydrogenase [cytochrome], mitochondrial	FAD-binding oxidoreductase/transferase type 4 family	1.1.2.4	Mitochondrion	(121)		FAD	1	(118)
AT5G08740	Alternative NAD(P)H-ubiquinone oxidoreductase C1, chloroplastic/mitochondrial	NADH dehydrogenase family	1.6.5.9; 1.6.5.12	Mitochondrion ; Mitochondrion inner membrane; Peripheral membrane protein Matrix side; Plastid, chloroplast, plastoglobule	(89, 110, 124)		FAD	1	(123)
AT5G14760	L-aspartate oxidase, chloroplastic	FAD-dependent oxidoreductase 2 family, NadB subfamily	1.4.3.16	Plastid, chloroplast	(128)		FAD	1	(127)
AT5G21482	Cytokinin dehydrogenase 7	Oxygen-dependent FAD-linked oxidoreductase family	1.5.99.12			extracellular region	FAD	1	(132)
AT5G23300	Dihydroorotate dehydrogenase	Dihydroorotate dehydrogenase family, Type 2 subfamily	1.3.5.2			Mitochondrion inner membrane, Single-pass membrane protein	FMN	1	(133)
AT5G24850	Cryptochrome DASH, chloroplastic/mitochondrial	DNA photolyase class-1 family	N/A	Plastid, chloroplast	(135)		FAD, MTHF	1	(134)
AT5G44440	Berberine bridge enzyme-like 28	Oxygen-dependent FAD-linked oxidoreductase family	1.1.1.-			cytoplasm, chloroplast	FAD	1	(138)
AT5G54500	NAD(P)H dehydrogenase (quinone) FQR1	WrbA family	1.6.5.2	Cell membrane	(86)		FMN	1	(143)
AT5G57360	Adagio protein 1	ADAGIO family	N/A	Nucleus; Cytoplasm	(49)		FMN	1	(144)
AT5G58140	Phototropin-2	Protein kinase superfamily, AGC Ser/Thr protein kinase family	2.7.11.1	Cell membrane; Peripheral membrane protein	(145)		FMN	1	(92)
AT5G63910	Farnesylcysteine lyase	Prenylcysteine oxidase family	1.8.3.6			vacuole, extracellular region, lysosome, vacuolar membrane	FAD	1	(146)
AT1G01580	Ferric reduction oxidase 2	Ferric reductase (FRE) family	1.16.1.7	Cell membrane, Multi-pass membrane protein	(2)		FAD	2	(1)
AT1G01590	Ferric reduction oxidase 1	Ferric reductase (FRE) family	1.16.1.7			Cell membrane, Multi-pass membrane protein	FAD	2	(1)
AT1G01980	Berberine bjridge enzyme-like 1	Oxygen-dependent FAD-linked oxidoreductase family	1.1.1.-	Secreted, cell wall	(4)		FAD	2	(3)
AT1G03990	Long-chain-alcohol oxidase FAO1	GMC oxidoreductase family	1.1.3.20			integral component of membrane	FAD	2	(5)
AT1G04180	Indole-3-pyruvate monooxygenase YUCCA9	FMO family	1.14.13.168				FAD	2	(6)
AT1G04580	Aldehyde oxidase 4	Xanthine dehydrogenase family	1.2.3.1; 1.2.3.7	Cytoplasm	(11)		FAD, [2Fe-2S], Mo-molybdopterin	2	(10)
AT1G04610	Indole-3-pyruvate monooxygenase YUCCA3	FMO family	1.14.13.168			nucleus	FAD	2	(6)
AT1G06290	Acyl-coenzyme A oxidase 3, peroxisomal	Acyl-CoA oxidase family	1.3.3.6	Peroxisome	(15)		FAD	2	(14)
AT1G06310	Putative acyl-coenzyme A oxidase 3.2, peroxisomal	Acyl-CoA oxidase family	1.3.3.6			peroxisome	FAD	2	(14)
AT1G07180	External alternative NAD(P)H-ubiquinone oxidoreductase	NADH dehydrogenase family	1.6.5.9			Mitochondria, Peroxisome	FAD	2	(17)
AT1G09090	Respiratory burst oxidase homolog protein B	RBOH (TC 5.B.1.3) family	1.11.1.-			Membrane, Multi-pass membrane protein	FAD	2	(18)
AT1G11770	Berberine bridge enzyme-like 2	Oxygen-dependent FAD-linked oxidoreductase family	1.1.1.-			cell wall	FAD	2	(3)
AT1G12130	Flavin-containing monooxygenase FMO GS-OX-like 6	FMO family	1.8.-.-			nucleus	FAD	2	(19)
AT1G12140	Flavin-containing monooxygenase FMO GS-OX5	FMO family	1.14.13.237			nucleus	FAD	2	(20)
AT1G12160	Flavin-containing monooxygenase FMO GS-OX-like 1	FMO family	1.8.-.-			cellular component	FAD	2	(19)
AT1G19230	Respiratory burst oxidase homolog protein E	RBOH (TC 5.B.1.3) family	1.11.1.-			Membrane, Multi-pass membrane protein	FAD	2	(18)
AT1G19250	flavin-containing monooxygenase 1	FMO family	1.14.13.-			chloroplast	FAD	2	(25)
AT1G20020	Ferredoxin--NADP reductase, leaf isozyme 2, chloroplastic	Ferredoxin--NADP reductase type 1 family	1.18.1.2	chloroplast stroma, chloroplast thylakoid membrane, Peripheral membrane protein, Stromal side	(27)		FAD	2	(26)
AT1G21430	Indole-3-pyruvate monooxygenase YUCCA11	FMO family	1.14.13.168			extracellular region	FAD	2	(6)
AT1G23020	Ferric reduction oxidase 3, mitochondrial	Ferric reductase (FRE) family	1.16.1.7			Mitochondrion membrane, Multi-pass membrane protein	FAD	2	(1)
AT1G26380	Berberine bridge enzyme-like 3	Oxygen-dependent FAD-linked oxidoreductase family	1.1.1.-	Endoplasmic reticulum Cell membrane	(28)		FAD	2	(3)
AT1G26390	Berberine bridge enzyme-like 4	Oxygen-dependent FAD-linked oxidoreductase family	1.1.1.-			cytoplasm, extracellular region	FAD	2	(3)
AT1G26400	Berberine bridge enzyme-like 5	Oxygen-dependent FAD-linked oxidoreductase family	1.1.1.-				FAD	2	(3)
AT1G26410	Berberine bridge enzyme-like 6	Oxygen-dependent FAD-linked oxidoreductase family	1.1.1.-			cytoplasm	FAD	2	(3)
AT1G26420	Berberine bridge enzyme-like 7	Oxygen-dependent FAD-linked oxidoreductase family	1.1.1.-			extracellular region	FAD	2	(3)
AT1G30700	Berberine bridge enzyme-like 8	Oxygen-dependent FAD-linked oxidoreductase family	1.1.1.-			extracellular region	FAD	2	(3)
AT1G30710	Berberine bridge enzyme-like 9	Oxygen-dependent FAD-linked oxidoreductase family	1.1.1.-	Secreted, cell wall	(4)		FAD	2	(3)
AT1G30720	Berberine bridge enzyme-like 10	Oxygen-dependent FAD-linked oxidoreductase family	1.1.1.-			chloroplast, extracellular region, secretory vesicle	FAD	2	(3)
AT1G30730	Berberine bridge enzyme-like 11	Oxygen-dependent FAD-linked oxidoreductase family	1.1.1.-			cytoplasm, extracellular region	FAD	2	(3)
AT1G30740	Berberine bridge enzyme-like 12	Oxygen-dependent FAD-linked oxidoreductase family	1.1.1.-			vacuole, extracellular region, chloroplast, plasmodesma	FAD	2	(3)
AT1G30760	Berberine bridge enzyme-like 13	Oxygen-dependent FAD-linked oxidoreductase family	1.1.1.194; 1.1.1.195			cytoplasm, extracellular region	FAD	2	(3)
AT1G32300	L-gulonolactone oxidase 1	Oxygen-dependent FAD-linked oxidoreductase family	1.1.3.8			membrane, extracellular region	FAD	2	(29)
AT1G34575	Berberine bridge enzyme-like 14	Oxygen-dependent FAD-linked oxidoreductase family	1.1.1.-			cell wall, chloroplast	FAD	2	(3)
AT1G48030	Dihydrolipoyl dehydrogenase 1, mitochondrial	Class-I pyridine nucleotide-disulfide oxidoreductase family	1.8.1.4	Mitochondrion matrix	(32)		FAD	2	(31)
AT1G48605	Phosphopantothenoylcysteine decarboxylase	HFCD (homooligomeric flavin containing Cys decarboxylase) superfamily	4.1.1.36			cytoplasm	FMN	2	(33)
AT1G48850	Chorismate synthase, chloroplastic	Chorismate synthase family	4.2.3.5			nucleolus, chloroplast stroma, nucleus, plastid, cytosol, chloroplast	FMN	2	(34)
AT1G48910	Indole-3-pyruvate monooxygenase YUCCA10	FMO family	1.14.13.168			chloroplast	FAD	2	(6)
AT1G50940	Electron transfer flavoprotein subunit alpha, mitochondrial	ETF alpha-subunit/FixB family	N/A	Mitochondrion matrix	(36)		FAD	2	(37)
AT1G58440	Squalene epoxidase 1	Squalene monooxygenase family	1.14.14.17			integral component of membrane, extracellular region, endoplasmic reticulum	FAD	2	(38)
AT1G62540	Flavin-containing monooxygenase FMO GS-OX2	FMO family	1.14.13.237			nucleus,	FAD	2	(20)
AT1G62560	Flavin-containing monooxygenase FMO GS-OX3	FMO family	1.14.13.237			chloroplast, integral component of membrane	FAD	2	(20)
AT1G62570	Flavin-containing monooxygenase FMO GS-OX4	FMO family	1.14.13.237			nucleus	FAD	2	(20)
AT1G62580	Flavin-containing monooxygenase FMO GS-OX-like 7	FMO family	1.8.-.-			nucleus	FAD	2	(39)
AT1G62600	Flavin-containing monooxygenase FMO GS-OX-like 4	FMO family	1.8.-.-			nucleus, vacuole	FAD	2	Uniprot, curated
AT1G62620	Flavin-containing monooxygenase FMO GS-OX-like 3	FMO family	1.8.-.-			nucleus	FAD	2	Uniprot, curated
AT1G62830	Lysine-specific histone demethylase 1 homolog 1	Flavin monoamine oxidase family	1.-	Nucleus, Cytoplasm	(41)		FAD	2	(40)
AT1G63370	Flavin-containing monooxygenase FMO GS-OX-like 5	FMO family	1.8.-.-			nucleus	FAD	2	Uniprot, curated
AT1G63940	Monodehydroascorbate reductase, chloroplastic/mitochondrial	FAD-dependent oxidoreductase family	1.6.5.4	Isoform MDAR6]: Plastid, chloroplast Isoform MDAR5]: Mitochondrion	(43)		FAD	2	(42)
AT1G64060	Respiratory burst oxidase homolog protein F	RBOH (TC 5.B.1.3) family	1.11.1.-	Cell membrane, Multi-pass membrane protein	(44)		FAD	2	(18)
AT1G65860	Flavin-containing monooxygenase FMO GS-OX1	FMO family	1.14.13.237			nucleus	FAD	2	(47)
AT1G68050	Adagio protein 3	ADAGIO family	N/A	Nucleus, Cytoplasm	(49)		FMN	2	(48)
AT1G72970	Protein HOTHEAD	GMC oxidoreductase family	1.1.			extracellular region	FAD	2	(52)

Table S1. Flavoproteins of Arabidopsis thaliana. The list has been collected by searching the TAIR, Gene Ontology, UniProt and PDB database for flavoproteins associated terms.

Gene Locus	Protein Name	Protein Family	E.C.	Localization (Literature)	Reference (Localization)	Localization (predicted)	Cofactor	Evidence Code	Reference (Flavin binding)
AT1G73050	(R)-mandelonitrile lyase-like	GMC oxidoreductase family	4.1.2.10				FAD	2	(53)
AT1G75450	Cytokinin dehydrogenase 5	Oxygen-dependent FAD-linked oxidoreductase family	1.5.99.12			extracellular space, extracellular region	FAD	2	(54)
AT1G76690	12-oxophytodienoate reductase 2	NADH:flavin oxidoreductase/NADH oxidase family	1.3.1.42; 1.3.1.-	Cytoplasm	(56)		FMN	2	(57)
AT1G77760	Nitrate reductase [NADH] 1	Nitrate reductase family	1.7.1.1			cytosol, chloroplast	FAD, heme, Mo-molybdopterin	2	(30)
AT2G01270	Sulfhydryl oxidase 2		1.8.3.2			Golgi apparatus, Golgi trans cisterna, extracellular space, extracellular region, integral component of Golgi membrane, trans-Golgi network, endosome	FAD	2	(23)
AT2G02710	Protein TWIN LOV 1		N/A			nucleus	FMN	2	(58)
AT2G17420	Thioredoxin reductase 2	Class-II pyridine nucleotide-disulfide oxidoreductase family	1.8.1.9	Cytoplasm, Mitochondrion matrix	(60)		FAD	2	(60)
AT2G18450	Succinate dehydrogenase [ubiquinone] flavoprotein subunit 2, mitochondrial	FAD-dependent oxidoreductase 2 family, FRD/SDH subfamily	1.3.5.1	Mitochondrion inner membrane ; Peripheral membrane protein ; Matrix side	(32)		FAD	2	(61)
AT2G18915	Adagio protein 2	ADAGIO family	N/A	Nucleus, Cytoplasm	(49)		FMN	2	(48)
AT2G19500	Cytokinin dehydrogenase 2	Oxygen-dependent FAD-linked oxidoreductase family	1.5.99.12	Endoplasmic reticulum; Secreted, extracellular space	(62)		FAD	2	(54)
AT2G20800	External alternative NAD(P)H-ubiquinone oxidoreductase	NADH dehydrogenase family	1.6.5.9	Mitochondrion inner membrane; Peripheral membrane protein; Intermembrane side	(65)		FAD	2	(64)
AT2G22830	Squalene epoxidase 2, mitochondrial	Squalene monooxygenase family	1.14.14.17			mitochondrial membrane	FAD	2	(38)
AT2G27150	Abscisic-aldehyde oxidase	Xanthine dehydrogenase family	1.2.3.14; 1.2.3.7	Cytoplasm	(67)		FAD, [2Fe-2S], Mo-molybdopterin	2	(10)
AT2G29990	External alternative NAD(P)H-ubiquinone oxidoreductase	NADH dehydrogenase family	1.6.5.9	Mitochondrion inner membrane ; Peripheral membrane protein ; Matrix side	(65)	Mitochondrion inner membrane; Peripheral membrane protein; Matrix side; Peroxisome	FAD	2	(17)
AT2G33230	Indole-3-pyruvate monooxygenase YUCCA7	FMO family	1.14.13.168				FAD	2	(6)
AT2G34810	Berberine bridge enzyme-like 16	Oxygen-dependent FAD-linked oxidoreductase family	1.1.1.-			cytoplasm, extracellular region	FAD	2	(3)
AT2G35690	Putative peroxisomal acyl-coenzyme A oxidase 1.2	Acyl-CoA oxidase family	1.3.3.6	Peroxisome	(14)		FAD	2	(14)
AT2G41220	Ferredoxin-dependent glutamate synthase 2, chloroplastic	Glutamate synthase family	1.4.7.1			chloroplast, chloroplast envelope, chloroplast stroma, plastid	FAD, FMN, [3Fe-4S]	2	(68)
AT2G41510	Cytokinin dehydrogenase 1	Oxygen-dependent FAD-linked oxidoreductase family	1.5.99.12	Vacuole	(62)		FAD	2	(54)
AT2G41680	NADPH-dependent thioredoxin reductase 3	Class-II pyridine nucleotide-disulfide oxidoreductase family	1.8.1.9	chloroplast	(70)		FAD	2	(69)
AT2G43400	Electron transfer flavoprotein-ubiquinone oxidoreductase, mitochondrial	ETF-QO/FixC family	1.5.5.1	Mitochondrion inner membrane	(37)		FAD, [4Fe-4S]	2	(37)
AT2G46580	Pyridoxine/pyridoxamine 5'-phosphate oxidase 2	Pyridoxamine 5'-phosphate oxidase family	1.4.3.5				FMN	2	(71)
AT2G46740	L-gulonolactone oxidase 5	Oxygen-dependent FAD-linked oxidoreductase family	1.1.3.8			membrane, extracellular region	FAD	2	(29)
AT2G46750	L-gulonolactone oxidase 2	Oxygen-dependent FAD-linked oxidoreductase family	1.1.3.8			membrane, extracellular region	FAD	2	(29)
AT2G46760	L-gulonolactone oxidase 6	Oxygen-dependent FAD-linked oxidoreductase family	1.1.3.8			membrane, chloroplast	FAD	2	(29)
AT3G02280	NADPH-dependent diflavin oxidoreductase 1	NADPH-dependent diflavin oxidoreductase NDOR1 family	1.18.1.-	Cytoplasm . Nucleus	(72)		FAD, FMN	2	(72)
AT3G06810		Encodes a protein with similarity to acyl-CoA dehydrogenases.	N/A	Peroxisome	(73)		FAD	2	(73)
AT3G09940	Monodehydroascorbate reductase 3	FAD-dependent oxidoreductase family	1.6.5.4	Cytoplasm	(74)		FAD	2	(74)
AT3G10390	Protein FLOWERING LOCUS D	Flavin monoamine oxidase family	1.-			mitochondrion, chloroplast,	FAD	2	(40)
AT3G13682	Lysine-specific histone demethylase 1 homolog 2	Flavin monoamine oxidase family	1.-			mitochondrion,	FAD	2	(40)
AT3G16950	Dihydrolipoyl dehydrogenase 1, chloroplastic	Class-I pyridine nucleotide-disulfide oxidoreductase family	1.8.1.4	Plastid, chloroplast stroma	(79)		FAD	2	(79)
AT3G17240	Dihydrolipoyl dehydrogenase 2, mitochondrial	Class-I pyridine nucleotide-disulfide oxidoreductase family	1.8.1.4	Mitochondrion matrix	(32)		FAD	2	(31)
AT3G19820	Delta(24)-sterol reductase	DIMINUTO family	1.3.1.72	Microsome membrane; Single-pass type II membrane protein ; Cytoplasmic side	(82)		FAD	2	(81)
AT3G23410	Long-chain-alcohol oxidase FAO3	GMC oxidoreductase family	1.1.3.20	Membrane	(5)		FAD	2	(5)
AT3G24170	Glutathione reductase, cytosolic	Class-I pyridine nucleotide-disulfide oxidoreductase family	1.8.1.7	Cytoplasm, Peroxisome	(84)		FAD	2	(83)
AT3G27820	Monodehydroascorbate reductase 4, peroxisomal	FAD-dependent oxidoreductase family	1.6.5.4	Peroxisome membrane	(74)		FAD	2	(74)
AT3G30775	Proline dehydrogenase 1, mitochondrial	Proline oxidase family	1.5.5.2	Mitochondrion	(88)		FAD	2	(87)
AT3G43600	Indole-3-acetaldehyde oxidase	Xanthine dehydrogenase family	1.2.3.7			cytoplasm	FAD, [2Fe-2S], Mo-molybdopterin	2	(10)
AT3G44190		FAD/NAD(P)-binding oxidoreductase family protein	1.3.1.-			cytoplasm, Golgi apparatus	FAD	2	(89)
AT3G45300	Isovaleryl-CoA dehydrogenase, mitochondrial	Acyl-CoA dehydrogenase family	1.3.8.4	Mitochondrion	(91)		FAD	2	(90)
AT3G45810	Putative respiratory burst oxidase homolog protein J	RBOH (TC 5.B.1.3) family	1.11.1.-			Membrane, Multi-pass membrane protein	FAD	2	(18)
AT3G52880	Monodehydroascorbate reductase 1, peroxisomal	FAD-dependent oxidoreductase family	1.6.5.4	Peroxisome matrix	(74)		FAD	2	(74)
AT3G54660	Glutathione reductase, chloroplastic	Class-I pyridine nucleotide-disulfide oxidoreductase family	1.8.1.7	Plastid, chloroplast, Mitochondrion	(97)		FAD	2	(83)
AT3G59050	Polyamine oxidase 3	Flavin monoamine oxidase family	1.5.3.17	Peroxisome	(46)		FAD	2	(45)
AT3G63440	Cytokinin dehydrogenase 6	Oxygen-dependent FAD-linked oxidoreductase family	1.5.99.12			extracellular space, extracellular region	FAD	2	(54)
AT4G01690	Protoporphyrinogen oxidase 1, chloroplastic	Protoporphyrinogen oxidase family	1.3.3.4	Plastid, chloroplast	(101)		FAD	2	(101)
AT4G05020	External alternative NAD(P)H-ubiquinone oxidoreductase	NADH dehydrogenase family	1.6.5.9	Mitochondrion inner membrane; Peripheral membrane protein	(65)		FAD	2	(64)
AT4G11230	Respiratory burst oxidase homolog protein I	RBOH (TC 5.B.1.3) family	1.11.1.-			Membrane, Multi-pass membrane protein	FAD	2	(18)
AT4G13260	Indole-3-pyruvate monooxygenase YUCCA2	FMO family	1.14.13.168			nucleus	FAD	2	(6)
AT4G14210	15-cis-phytoene desaturase, chloroplastic/chromoplastic	Carotenoid/retinoid oxidoreductase family	1.3.5.5			chloroplast, chloroplast envelope, cytosol, chloroplast thylakoid	FAD	2	(105)
AT4G15760	Monooxygenase 1	3-hydroxybenzoate 6-hydroxylase family	1.14.13.-			mitochondrion, endoplasmic reticulum	FAD	2	(106)
AT4G16155	Dihydrolipoyl dehydrogenase 2, chloroplastic	Class-I pyridine nucleotide-disulfide oxidoreductase family	1.8.1.4			chloroplast envelope, chloroplast stroma, cytosol, chloroplast	FAD	2	(79)
AT4G16310	Lysine-specific histone demethylase	Flavin monoamine oxidase family	1.-	Nucleus	(107)		FAD	2	(40)
AT4G19380	Long-chain-alcohol oxidase FAO4A	GMC oxidoreductase family	1.1.3.20			Membrane; Single-pass mem	FAD	2	(5)
AT4G20800	Berberine bridge enzyme-like 17	Oxygen-dependent FAD-linked oxidoreductase family	1.1.1.-			cytoplasm, extracellular region	FAD	2	(3)
AT4G20820	Berberine bridge enzyme-like 18	Oxygen-dependent FAD-linked oxidoreductase family	1.1.1.-			chloroplast	FAD	2	(3)
AT4G20830	Berberine bridge enzyme-like 19	Oxygen-dependent FAD-linked oxidoreductase family	1.1.1.-	Extracellular, plasma membrane	(3)		FAD	2	(3)
AT4G20840	Berberine bridge enzyme-like 21	Oxygen-dependent FAD-linked oxidoreductase family	1.1.1.-			plasma membrane, cytosol, apoplast	FAD	2	(3)
AT4G20860	Berberine bridge enzyme-like 22	Oxygen-dependent FAD-linked oxidoreductase family	1.1.1.-	Plasma membrane, cytosol	(3)		FAD	2	(3)
AT4G21490	External alternative NAD(P)H-ubiquinone oxidoreductase	NADH dehydrogenase family	1.6.5.9	Peroxisome	(89)		FAD	2	(110)
AT4G24520	NADPH--cytochrome P450 reductase 1	NADPH--cytochrome P450 reductase family; Flavodoxin family; Flavoprotein pyridine nucleotide cytochrome reductase family	1.6.2.4			endoplasmic reticulum membrane, endoplasmic reticulum, cytosol, chloroplast, integral component of membrane	FAD, FMN	2	(111)
AT4G25090	Putative respiratory burst oxidase homolog protein G	RBOH (TC 5.B.1.3) family	1.11.1.-			Membrane, Multi-pass membrane protein	FAD	2	(18)
AT4G28220	External alternative NAD(P)H-ubiquinone oxidoreductase	NADH dehydrogenase family	1.6.5.9			Mitochondria, Peroxisome	FAD	2	(64)
AT4G28570	Long-chain-alcohol oxidase FAO4B	GMC oxidoreductase family	1.1.3.20			Membrane; Single-pass mem	FAD	2	(5)
AT4G28720	Indole-3-pyruvate monooxygenase YUCCA8	FMO family	1.14.13.168			nucleus	FAD	2	(6)
AT4G29740	Cytokinin dehydrogenase 4	Oxygen-dependent FAD-linked oxidoreductase family	1.5.99.12			Secreted, extracellular space	FAD	2	(54)
AT4G32540	indole-3-pyruvate monooxygenase YUCCA1	FMO family	1.14.13.168				FAD	2	(6)
AT4G34900	Xanthine dehydrogenase 2	Xanthine dehydrogenase family	1.17.1.4				FAD, [2Fe-2S], Mo-molybdopterin	2	(116)
AT4G37760	Squalene epoxidase 3	Squalene monooxygenase family	1.14.14.17			Membrane, Multi-pass membrane protein	FAD	2	(119)
AT4G38540	Monooxygenase 2	3-hydroxybenzoate 6-hydroxylase family	1.14.13.-			plasma membrane, mitochondrion	FAD	2	(106)
AT5G03630	Monodehydroascorbate reductase 2	FAD-dependent oxidoreductase family	1.6.5.4	Cytoplasm	(74)		FAD	2	(74)
AT5G04140	Ferredoxin-dependent glutamate synthase 1, chloroplastic/mitochondrial	Glutamate synthase family	1.4.7.1	chloroplast stroma, Mitochondrion matrix	(120)		FAD, FMN, [3Fe-4S]	2	(68)
AT5G05320	Monooxygenase 3	3-hydroxybenzoate 6-hydroxylase family	1.14.13.-			chloroplast	FAD	2	(106)
AT5G07390	Respiratory burst oxidase homolog protein A	RBOH (TC 5.B.1.3) family	1.11.1.-			Membrane, Multi-pass membrane protein	FAD	2	(18)
AT5G08530	NADH dehydrogenase [ubiquinone] flavoprotein 1, mitochondrial	Complex I 51 kDa subunit family	1.6.99.3	Mitochondrion inner membrane	(63)		FMN, [4Fe-4S]	2	(122)
AT5G11320	Indole-3-pyruvate monooxygenase YUCCA4	FMO family	1.14.13.168			[Isoform 1]: Cytoplasm.; [Isoform 2]: Endoplasmic reticulum membrane; Single-pass membrane protein; Cytoplasmic side	FAD	2	(6)
AT5G11540	L-gulonolactone oxidase 3	Oxygen-dependent FAD-linked oxidoreductase family	1.1.3.8	Vacuole	(125)		FAD	2	(29)
AT5G13700	Polyamine oxidase 1	Flavin monoamine oxidase family	1.5.3.16; 1.5.3.17	Cytoplasm	(126)		FAD	2	(45)
AT5G14220	Protoporphyrinogen oxidase 2, chloroplastic/mitochondrial	Protoporphyrinogen oxidase family	1.3.3.4	Plastid, chloroplast. Mitochondrion	(65, 101)		FAD	2	(101)
AT5G17770	NADH--cytochrome b5 reductase 1	Flavoprotein pyridine nucleotide cytochrome reductase family	1.6.2.2	Mitochondrion outer membrane, Single-pass membrane protein	(130)		FAD	2	(129)
AT5G20960	Indole-3-acetaldehyde oxidase	Xanthine dehydrogenase family	1.2.3.7			Cytoplasm	FAD, [2Fe-2S], Mo-molybdopterin	2	(10)
AT5G22140		FAD/NAD(P)-binding oxidoreductase family protein	1.-			Golgi apparatus, cytoplasm, plasma membrane, chloroplast, plasmodesma	FAD	2	(89)
AT5G23980	Ferric reduction oxidase 4	Ferric reductase (FRE) family	1.16.1.7			Membrane, Multi-pass membrane protein	FAD	2	(1)
AT5G23990	Ferric reduction oxidase 5	Ferric reductase (FRE) family	1.16.1.7			Cell membrane, Multi-pass membrane protein	FAD	2	(1)
AT5G24140	Squalene epoxidase 4	Squalene monooxygenase family	1.14.14.17			Membrane, Multi-pass membrane protein	FAD	2	(38)
AT5G24150	Squalene epoxidase 5	Squalene monooxygenase family	1.14.14.17			Membrane, Multi-pass membrane protein	FAD	2	(38)
AT5G24160	Squalene epoxidase 6	Squalene monooxygenase family	1.14.14.17			Membrane, Multi-pass membrane protein	FAD	2	(38)
AT5G25620	Indole-3-pyruvate monooxygenase YUCCA6	FMO family	1.14.13.168	Cytoplasm	(136)		FAD	2	(6)
AT5G38710	Proline dehydrogenase 2, mitochondrial	Proline oxidase family	1.5.5.2	Mitochondrion	(87)		FAD	2	(87)
AT5G43430	Electron transfer flavoprotein subunit beta, mitochondrial	ETF beta-subunit/FixA family	N/A	Mitochondrion	(32)		FAD, AMP	2	(137)
AT5G43890	Indole-3-pyruvate monooxygenase YUCCA5	FMO family	1.14.13.168			cellular component	FAD	2	(6)
AT5G44360	Berberine bridge enzyme-like 23	Oxygen-dependent FAD-linked oxidoreductase family	1.1.1.-	Secreted, cell wall	(4)		FAD	2	(3)
AT5G44380	Berberine bridge enzyme-like 24	Oxygen-dependent FAD-linked oxidoreductase family	1.1.1.-			cell wall, extracellular region, cytoplasm, chloroplast, plasmodesma	FAD	2	(3)

Table S1. Flavoproteins of Arabidopsis thaliana. The list has been collected by searching the TAIR, Gene Ontology, UniProt and PDB database for flavoproteins associated terms.

Gene Locus	Protein Name	Protein Family	E.C.	Localization (Literature)	Reference (Localization)	Localization (predicted)	Cofactor	Evidence Code	Reference (Flavin binding)
AT5G44390	Berberine bridge enzyme-like 25	Oxygen-dependent FAD-linked oxidoreductase family	1.1.1.-			chloroplast, extracellular region	FAD	2	(3)
AT5G44400	Berberine bridge enzyme-like 26	Oxygen-dependent FAD-linked oxidoreductase family	1.1.1.-			cell wall, extracellular region, chloroplast, plasmodesma	FAD	2	(3)
AT5G44410	Berberine bridge enzyme-like 27	Oxygen-dependent FAD-linked oxidoreductase family	1.1.1.-	Secreted, cell wall	(4)		FAD	2	(3)
AT5G47910	Respiratory burst oxidase homolog protein D	RBOH (TC 5.B.1.3) family	1.11.1.-			Membrane, Multi-pass membrane protein	FAD	2	(18)
AT5G49730	Ferric reduction oxidase 6	Ferric reductase (FRE) family	1.16.1.7			Cell membrane, Multi-pass membrane protein	FAD	2	(1)
AT5G49740	Ferric reduction oxidase 7, chloroplastic	Ferric reductase (FRE) family	1.16.1.7			chloroplast membrane, Multi-pass membrane protein	FAD	2	(1)
AT5G49970	Pyridoxine/pyridoxamine 5'-phosphate oxidase 1, chloroplastic	NnrE/AIBP family; Pyridoxamine 5'-phosphate oxidase family	1.4.3.5	Plastid, chloroplast	(140)		FMN, K^+	2	(139)
AT5G50160	Ferric reduction oxidase 8, mitochondrial	Ferric reductase (FRE) family	1.16.1.7			Mitochondrion membrane, Multi-pass membrane protein	FAD	2	(1)
AT5G51060	Respiratory burst oxidase homolog protein C	RBOH (TC 5.B.1.3) family	1.11.1.-			Mitochondrion membrane, Multi-pass membrane protein	FAD	2	(18)
AT5G53460	Glutamate synthase 1 [NADH], chloroplastic	Glutamate synthase family	1.4.1.14	Plastid, chloroplast	(142)		FMN, FAD, [3Fe-4S]	2	(141)
AT5G56490	L-gulonolactone oxidase 4	Oxygen-dependent FAD-linked oxidoreductase family	1.1.3.8			membrane, extracellular region	FAD	2	(29)
AT5G56970	Cytokinin dehydrogenase 3	Oxygen-dependent FAD-linked oxidoreductase family	1.5.99.12	Endoplasmic reticulum; Vacuole	(62)		FAD	2	(54)
AT5G60010	Putative respiratory burst oxidase homolog protein H	RBOH (TC 5.B.1.3) family	1.11.1.			Membrane, Multi-pass membrane protein	FAD	2	(18)
AT5G61290	Flavin-containing monooxygenase FMO GS-OX-like 8	FMO family	1.8.-.-			chloroplast	FAD	2	Uniprot, curated
AT5G65110	Acyl-coenzyme A oxidase 2, peroxisomal	Acyl-CoA oxidase family	1.3.3.6	Peroxisome	(147)		FAD	2	(14)
AT5G66190	Ferredoxin--NADP reductase, leaf isozyme 1, chloroplastic	Ferredoxin--NADP reductase type 1 family	1.18.1.2	chloroplast stroma, chloroplast thylakoid membrane, Peripheral membrane protein, Stromal side	(27)		FAD	2	(26)
AT5G66760	Succinate dehydrogenase [ubiquinone] flavoprotein subunit 1, mitochondrial	FAD-dependent oxidoreductase 2 family, FRD/SDH subfamily	1.3.5.1	Mitochondrion inner membrane ; Peripheral membrane protein ; Matrix side	(32, 63)		FAD	2	(61)
AT5G67030	Zeaxanthin epoxidase, chloroplastic		1.14.15.21	membrane, chloroplast envelope, plastid, chloroplast			FAD	2	(148)
AT1G09400	Putative 12-oxophytodienoate reductase-like protein 1	NADH:flavin oxidoreductase/NADH oxidase family	1.3.1.-			cytoplasm	FMN	3	
AT1G12200	Flavin-containing monooxygenase FMO GS-OX-like 2	FMO family	1.8.-.-			nucleus	FAD	3	
AT1G12570		Ortholog of maize IPE1 gene which is involved in pollen exine development.	1.1.-			extracellular region	FAD	3	
AT1G14185		Glucose-methanol-choline (GMC) oxidoreductase family protein	1.1.-			chloroplast	FAD	3	
AT1G14190		Glucose-methanol-choline (GMC) oxidoreductase family protein	1.1.			chloroplast, nucleus	FAD	3	
AT1G17990	Putative 12-oxophytodienoate reductase-like protein 2A	NADH:flavin oxidoreductase/NADH oxidase family	1.3.1.-			cytoplasm	FMN	3	
AT1G18020	Putative 12-oxophytodienoate reductase-like protein 2B	NADH:flavin oxidoreductase/NADH oxidase family	1.3.1.-			nucleus	FMN	3	
AT1G24340		A locus involved in embryogenesis. Mutations in this locus result in embryo lethality. The mRNA is cell-to-cell mobile.	N/A			cytoplasm	FAD	3	
AT1G30510	Ferredoxin--NADP reductase, root isozyme 2, chloroplastic	Ferredoxin--NADP reductase type 1 family	1.18.1.2			chloroplast	FAD	3	
AT1G55930	DUF21 domain-containing protein At1g55930, chloroplastic		N/A			chloroplast membrane, chloroplast envelope, plastid, nucleus	FAD	3	
AT1G63340	Putative flavin-containing monooxygenase FMO GS-OX-like 10	FMO family	1.8.-.-			nucleus	FAD	3	
AT1G63390	Putative flavin-containing monooxygenase FMO GS-OX-like 11	FMO family	1.8.-.-				FAD	3	
AT1G75200	S-adenosyl-L-methionine-dependent tRNA 4-demethylwyosine synthase	TYW1 family	4.1.3.44			chloroplast, mitochondrion	FMN, [4Fe-4S]	3	
AT2G13440		glucose-inhibited division family A protein;(source:Araport11)	N/A			mitochondrion	FAD	3	
AT2G20360	NADH dehydrogenase [ubiquinone] 1 alpha subcomplex subunit 9	Complex I NDUFA9 subunit family	N/A	Mitochondrion matrix	(63)		FAD	3	
AT2G29720		Encodes CTF2B.	N/A			mitochondrion	FAD	3	
AT2G35660		Encodes a member of a novel gene family with homology to known proteins involved in hydroxylation and oxidation of an aromatic ring.	1.5.5.1			chloroplast, mitochondrion	FAD	3	
AT2G44160	Methylenetetrahydrofolate reductase 2	Methylenetetrahydrofolate reductase family	1.5.1.20			cytosol, nucleus	FAD	3	
AT2G47590	Blue-light photoreceptor PHR2	DNA photolyase class-1 family	N/A			nucleus	FAD	3	
AT3G06690	Putative acyl-coenzyme A oxidase At3g06690	Acyl-CoA oxidase family	1.3.3.6			cytosol, nucleus, peroxisome	FAD	3	
AT3G13070	Putative DUF21 domain-containing protein At3g13070, chloroplastic		N/A			chloroplast membrane; Mul	FAD	3	
AT3G14130	Peroxisomal (S)-2-hydroxy-acid oxidase GLO4	FMN-dependent alpha-hydroxy acid dehydrogenase family	1.1.3.15			Peroxisome	FMN	3	
AT3G14150	Peroxisomal (S)-2-hydroxy-acid oxidase GLO3	FMN-dependent alpha-hydroxy acid dehydrogenase family	1.1.3.15	Peroxisome	(76)		FMN	3	
AT3G49640		Aldolase-type TIM barrel family protein;(source:Araport11)	1.1.-			chloroplast, nucleus	FAD	3	
AT3G56060		Glucose-methanol-choline (GMC) oxidoreductase family protein	1.3.1.-			chloroplast	FAD	3	
AT3G63510		FMN-linked oxidoreductases superfamily protein;(source:Araport11)	1.-			chloroplast	FAD	3	
AT4G05390	Ferredoxin--NADP reductase, root isozyme 1, chloroplastic	Ferredoxin--NADP reductase type 1 family	1.18.1.2			chloroplast	FAD	3	
AT4G27270	NAD(P)H dehydrogenase (quinone) FQR1-like 1	WrbA family	1.6.5.2	Cell membrane	(86)		FMN	3	
AT4G36750	NAD(P)H dehydrogenase (quinone) FQR1-like 2	WrbA family	1.6.5.2	Cell membrane	(86)		FMN	3	
AT4G38890	tRNA-dihydrouridine	Dus family, Dus3 subfamily	1.3.1.-			nucleus, vacuole	FAD	3	
AT5G07800	Flavin-containing monooxygenase FMO GS-OX-like 9	FMO family	1.8.-.-			nucleus	FAD	3	
AT5G11330		FAD/NAD(P)-binding oxidoreductase family protein	1.3.1.-			chloroplast	FAD	3	
AT5G20080	NADH-cytochrome b5 reductase-like protein	Flavoprotein pyridine nucleotide cytochrome reductase family	1.6.2.2	Mitochondrion	(131)		FAD	3	
AT5G45180	Putative flavin-containing monooxygenase 2	FMO family	1.14.13.-				FAD	3	
AT5G47970		Aldolase-type TIM barrel family protein	4.-			phragmoplast, nucleus, cytoplasm, cytosol, chloroplast	FAD	3	
AT5G51950		GMC oxidoreductase family protein	1.3.1.-			chloroplast	FAD	3	
AT5G56470	Truncated L-gulonolactone oxidase 7, mitochondrial	Oxygen-dependent FAD-linked oxidoreductase family	1.1.3.8			membrane, mitochondrion		3	(29)
AT5G58800	NAD(P)H dehydrogenase (quinone) FQR1-like 3	WrbA family	1.6.5.2			cytoplasm, plasma membrane, plastid	FMN	3	
AT5G67220		FMN-linked oxidoreductases superfamily protein	1.-			mitochondrion	FAD	3	

References

1. Wu, H., et al., Molecular and biochemical characterization of the Fe(III) chelate reductase gene family in Arabidopsis thaliana. Plant Cell Physiol, 2005. 46(9): p. 1505-14.

2. Durrett, T.P., E.L. Connolly, and E.E. Rogers, Arabidopsis cpFtsY mutants exhibit pleiotropic defects including an inability to increase iron deficiency-inducible root Fe(III) chelate reductase activity. Plant J, 2006. 47(3): p. 467-79.

3. Daniel, B., et al., Oxidation of Monolignols by Members of the Berberine Bridge Enzyme Family Suggests a Role in Plant Cell Wall Metabolism. J Biol Chem, 2015. 290(30): p. 18770-81.

4. Irshad, M., et al., A new picture of cell wall protein dynamics in elongating cells of Arabidopsis thaliana: confirmed actors and newcomers. BMC Plant Biol, 2008. 8: p. 94.

5. Cheng, Q., et al., Functional identification of AtFao3, a membrane bound long chain alcohol oxidase in Arabidopsis thaliana. FEBS Lett, 2004. 574(1-3): p. 62-8.

6. Cao, X., et al., The Roles of Auxin Biosynthesis YUCCA Gene Family in Plants. Int J Mol Sci, 2019. 20(24).

7. Sancar, A., Structure and function of DNA photolyase. Biochemistry, 1994. 33(1): p. 2-9.

8. Jourdan, N., et al., Blue-light dependent ROS formation by Arabidopsis cryptochrome-2 may contribute toward its signaling role. Plant Signal Behav, 2015. 10(8): p. e1042647.

9. Kleiner, O., et al., Nuclear localization of the Arabidopsis blue light receptor cryptochrome 2. Plant J, 1999. 19(3): p. 289-96.

10. Srivastava, S., et al., Aldehyde Oxidase 4 Plays a Critical Role in Delaying Silique Senescence by Catalyzing Aldehyde Detoxification. Plant Physiol, 2017. 173(4): p. 1977-1997.

11. Ibdah, M., et al., An aldehyde oxidase in developing seeds of Arabidopsis converts benzaldehyde to benzoic Acid. Plant Physiol, 2009. 150(1): p. 416-23.

12. Wang, X. and L. Liu, Crystal Structure and Catalytic Mechanism of 7-Hydroxymethyl Chlorophyll a Reductase. J Biol Chem, 2016. 291(25): p. 13349-59.

13. Meguro, M., et al., Identification of the 7-hydroxymethyl chlorophyll a reductase of the chlorophyll cycle in Arabidopsis. Plant Cell, 2011. 23(9): p. 3442-53.

14. Adham, A.R., et al., Mutations in Arabidopsis acyl-CoA oxidase genes reveal distinct and overlapping roles in beta-oxidation. Plant J, 2005. 41(6): p. 859-74.

15. Eastmond, P.J., et al., Promoter trapping of a novel medium-chain acyl-CoA oxidase, which is induced transcriptionally during Arabidopsis seed germination. J Biol Chem, 2000. 275(44): p. 34375-81.

16. Yu, Q., et al., Plant carotene cis-trans isomerase CRTISO: a new member of the FAD(RED)-dependent flavoproteins catalyzing non-redox reactions. J Biol Chem, 2011. 286(10): p. 8666-76.

17. Carrie, C., et al., Type II NAD(P)H dehydrogenases are targeted to mitochondria and chloroplasts or peroxisomes in Arabidopsis thaliana. FEBS Lett, 2008. 582(20): p. 3073-9.

18. Kaur, G. and P.K. Pati, In silico insights on diverse interacting partners and phosphorylation sites of respiratory burst oxidase homolog (Rbohs) gene families from Arabidopsis and rice. BMC Plant Biol, 2018. 18(1): p. 161.

19. Kong, W., et al., Two Novel Flavin-Containing Monooxygenases Involved in Biosynthesis of Aliphatic Glucosinolates. Front Plant Sci, 2016. 7: p. 1292.

20. Li, J., et al., Subclade of flavin-monooxygenases involved in aliphatic glucosinolate biosynthesis. Plant Physiol, 2008. 148(3): p. 1721-33.

21. Okafuji, A., et al., Light-induced activation of class II cyclobutane pyrimidine dimer photolyases. DNA Repair (Amst), 2010. 9(5): p. 495-505.

22. Kaiser, G., et al., Increased DNA repair in Arabidopsis plants overexpressing CPD photolyase. Planta, 2009. 230(3): p. 505-15.

23. Limor-Waisberg, K., et al., Phylogenetics and enzymology of plant quiescin sulfhydryl oxidase. FEBS Lett, 2012. 586(23): p. 4119-25.

24. Alejandro, S., et al., An Arabidopsis quiescin-sulfhydryl oxidase regulates cation homeostasis at the root symplast-xylem interface. EMBO J, 2007. 26(13): p. 3203-15.

25. Mishina, T.E. and J. Zeier, The Arabidopsis flavin-dependent monooxygenase FMO1 is an essential component of biologically induced systemic acquired resistance. Plant Physiol, 2006. 141(4): p. 1666-75.

26. Mulo, P., Chloroplast-targeted ferredoxin-NADP(+) oxidoreductase (FNR): structure, function and location. Biochim Biophys Acta, 2011. 1807(8): p. 927-34.

27. HANKE, G.T., et al., Multiple iso-proteins of FNR in Arabidopsis: evidence for different contributions to chloroplast function and nitrogen assimilation. Plant, Cell & Environment, 2005. 28(9): p. 1146-1157.

28. Inze, A., et al., A subcellular localization compendium of hydrogen peroxide-induced proteins. Plant Cell Environ, 2012. 35(2): p. 308-20.

29. Maruta, T., et al., The contribution of Arabidopsis homologs of L-gulono-1,4-lactone oxidase to the biosynthesis of ascorbic acid. Biosci Biotechnol Biochem, 2010. 74(7): p. 1494-7.

30. Meyer, C., et al., Is nitrate reductase a major player in the plant NO (nitric oxide) game? Photosynth Res, 2005. 83(2): p. 181-9.

31. Lutziger, I. and D.J. Oliver, Characterization of two cDNAs encoding mitochondrial lipoamide dehydrogenase from Arabidopsis. Plant Physiol, 2001. 127(2): p. 615-23.

32. Heazlewood, J.L., et al., Experimental analysis of the Arabidopsis mitochondrial proteome highlights signaling and regulatory components, provides assessment of targeting prediction programs, and indicates plant-specific mitochondrial proteins. Plant Cell, 2004. 16(1): p. 241-56.

33. Espinosa-Ruiz, A., et al., Arabidopsis thaliana AtHAL3: a flavoprotein related to salt and osmotic tolerance and plant growth. Plant J, 1999. 20(5): p. 529-39.

34. Schaller, A., et al., Molecular cloning and analysis of a cDNA coding for chorismate synthase from the higher plant Corydalis sempervirens Pers. J Biol Chem, 1991. 266(32): p. 21434-8.

35. Vitu, E., et al., Gain of function in an ERV/ALR sulfhydryl oxidase by molecular engineering of the shuttle disulfide. J Mol Biol, 2006. 362(1): p. 89-101.

36. Levitan, A., A. Danon, and T. Lisowsky, Unique features of plant mitochondrial sulfhydryl oxidase. J Biol Chem, 2004. 279(19): p. 20002-8.

37. Ishizaki, K., et al., The critical role of Arabidopsis electron-transfer flavoprotein:ubiquinone oxidoreductase during dark-induced starvation. Plant Cell, 2005. 17(9): p. 2587-600.

38. Rasbery, J.M., et al., Arabidopsis thaliana squalene epoxidase 1 is essential for root and seed development. J Biol Chem, 2007. 282(23): p. 17002-13.

39. Mulaudzi, T., et al., Identification of a novel Arabidopsis thaliana nitric oxide-binding molecule with guanylate cyclase activity in vitro. FEBS Lett, 2011. 585(17): p. 2693-7.

40. Spedaletti, V., et al., Characterization of a lysine-specific histone demethylase from Arabidopsis thaliana. Biochemistry, 2008. 47(17): p. 4936-47.

41. Krichevsky, A., et al., Involvement of KDM1C histone demethylase-OTLD1 otubain-like histone deubiquitinase complexes in plant gene repression. Proc Natl Acad Sci U S A, 2011. 108(27): p. 11157-62.

42. Johnston, E.J., et al., Monodehydroascorbate reductase mediates TNT toxicity in plants. Science, 2015. 349(6252): p. 1072-5.

43. Obara, K., K. Sumi, and H. Fukuda, The use of multiple transcription starts causes the dual targeting of Arabidopsis putative monodehydroascorbate reductase to both mitochondria and chloroplasts. Plant Cell Physiol, 2002. 43(7): p. 697-705.

44. Keller, T., et al., A plant homolog of the neutrophil NADPH oxidase gp91phox subunit gene encodes a plasma membrane protein with Ca2+ binding motifs. Plant Cell, 1998. 10(2): p. 255-66.

45. Fincato, P., et al., Functional diversity inside the Arabidopsis polyamine oxidase gene family. J Exp Bot, 2011. 62(3): p. 1155-68.

46. Kamada-Nobusada, T., et al., A putative peroxisomal polyamine oxidase, AtPAO4, is involved in polyamine catabolism in Arabidopsis thaliana. Plant Cell Physiol, 2008. 49(9): p. 1272-82.

47. Hansen, B.G., D.J. Kliebenstein, and B.A. Halkier, Identification of a flavin-monooxygenase as the S-oxygenating enzyme in aliphatic glucosinolate biosynthesis in Arabidopsis. Plant J, 2007. 50(5): p. 902-10.

48. Jarillo, J.A., et al., An Arabidopsis circadian clock component interacts with both CRY1 and phyB. Nature, 2001. 410(6827): p. 487-90.

49. Takase, T., et al., LOV KELCH PROTEIN2 and ZEITLUPE repress Arabidopsis photoperiodic flowering under non-inductive conditions, dependent on FLAVIN-BINDING KELCH REPEAT F-BOX1. Plant J, 2011. 67(4): p. 608-21.

50. Fan, F., et al., AtERO1 and AtERO2 Exhibit Differences in Catalyzing Oxidative Protein Folding in the Endoplasmic Reticulum. Plant Physiol, 2019. 180(4): p. 2022-2033.

51. Dixon, D.P., et al., Cloning and initial characterization of the Arabidopsis thaliana endoplasmic reticulum oxidoreductins. Antioxid Redox Signal, 2003. 5(4): p. 389-96.

52. Xu, Y., et al., HOTHEAD-Like HTH1 is Involved in Anther Cutin Biosynthesis and is Required for Pollen Fertility in Rice. Plant Cell Physiol, 2017. 58(7): p. 1238-1248.

53. Hu, Z. and J.E. Poulton, Molecular analysis of (R)-(+)-mandelonitrile lyase microheterogeneity in black cherry. Plant Physiol, 1999. 119(4): p. 1535-46.

54. Schmulling, T., et al., Structure and function of cytokinin oxidase/dehydrogenase genes of maize, rice, Arabidopsis and other species. J Plant Res, 2003. 116(3): p. 241-52.

55. Fox, B.G., et al., X-ray structure of Arabidopsis At1g77680, 12-oxophytodienoate reductase isoform 1. Proteins, 2005. 61(1): p. 206-8.

56. Strassner, J., et al., Characterization and cDNA-microarray expression analysis of 12-oxophytodienoate reductases reveals differential roles for octadecanoid biosynthesis in the local versus the systemic wound response. Plant J, 2002. 32(4): p. 585-601.

57. Beynon, E.R., et al., The role of oxophytodienoate reductases in the detoxification of the explosive 2,4,6-trinitrotoluene by Arabidopsis. Plant Physiol, 2009. 151(1): p. 253-61.

58. Ogura, Y., et al., Blue light diminishes interaction of PAS/LOV proteins, putative blue light receptors in Arabidopsis thaliana, with their interacting partners. J Plant Res, 2008. 121(1): p. 97-105.

59. Malone, T.E., et al., X-ray structure of Arabidopsis At2g06050, 12-oxophytodienoate reductase isoform 3. Proteins, 2005. 58(1): p. 243-5.

60. Reichheld, J.P., et al., AtNTRB is the major mitochondrial thioredoxin reductase in Arabidopsis thaliana. FEBS Lett, 2005. 579(2): p. 337-42.

61. Figueroa, P., et al., The four subunits of mitochondrial respiratory complex II are encoded by multiple nuclear genes and targeted to mitochondria in Arabidopsis thaliana. Plant Mol Biol, 2002. 50(4-5): p. 725-34.

62. Werner, T., et al., Cytokinin-deficient transgenic Arabidopsis plants show multiple developmental alterations indicating opposite functions of cytokinins in the regulation of shoot and root meristem activity. Plant Cell, 2003. 15(11): p. 2532-50.

63. Carrie, C., et al., Identification of cleavage sites and substrate proteins for two mitochondrial intermediate peptidases in Arabidopsis thaliana. J Exp Bot, 2015. 66(9): p. 2691-708.

64. Geisler, D.A., et al., Ca2+-binding and Ca2+-independent respiratory NADH and NADPH dehydrogenases of Arabidopsis thaliana. J Biol Chem, 2007. 282(39): p. 28455-64.

65. Klodmann, J., et al., Defining the protein complex proteome of plant mitochondria. Plant Physiol, 2011. 157(2): p. 587-98.

66. Goyer, A., et al., Characterization and metabolic function of a peroxisomal sarcosine and pipecolate oxidase from Arabidopsis. J Biol Chem, 2004. 279(17): p. 16947-53.

67. Nambara, E. and A. Marion-Poll, Abscisic acid biosynthesis and catabolism. Annu Rev Plant Biol, 2005. 56: p. 165-85.

68. Potel, F., et al., Assimilation of excess ammonium into amino acids and nitrogen translocation in Arabidopsis thaliana--roles of glutamate synthases and carbamoylphosphate synthetase in leaves. FEBS J, 2009. 276(15): p. 4061-76.

69. Bernal-Bayard, P., et al., Electron transfer pathways and dynamics of chloroplast NADPH-dependent thioredoxin reductase C (NTRC). J Biol Chem, 2012. 287(40): p. 33865-72.

70. Moon, J.C., et al., The C-type Arabidopsis thioredoxin reductase ANTR-C acts as an electron donor to 2-Cys peroxiredoxins in chloroplasts. Biochem Biophys Res Commun, 2006. 348(2): p. 478-84.

71. Sang, Y., et al., Identification of a second pyridoxine (pyridoxamine) 5′-phosphate oxidase in Arabidopsis thaliana. Acta Physiologiae Plantarum, 2011. 33(2): p. 559-566.

72. Varadarajan, J., et al., ATR3 encodes a diflavin reductase essential for Arabidopsis embryo development. New Phytol, 2010. 187(1): p. 67-82.

73. Zolman, B.K., M. Nyberg, and B. Bartel, IBR3, a novel peroxisomal acyl-CoA dehydrogenase-like protein required for indole-3-butyric acid response. Plant Mol Biol, 2007. 64(1-2): p. 59-72.

74. Lisenbee, C.S., M.J. Lingard, and R.N. Trelease, Arabidopsis peroxisomes possess functionally redundant membrane and matrix isoforms of monodehydroascorbate reductase. Plant J, 2005. 43(6): p. 900-14.

75. Shen, W., et al., Identification of a mitochondrial glycerol-3-phosphate dehydrogenase from Arabidopsis thaliana: evidence for a mitochondrial glycerol-3-phosphate shuttle in plants. FEBS Lett, 2003. 536(1-3): p. 92-6.

76. Reumann, S., et al., In-depth proteome analysis of Arabidopsis leaf peroxisomes combined with in vivo subcellular targeting verification indicates novel metabolic and regulatory functions of peroxisomes. Plant Physiol, 2009. 150(1): p. 125-43.

77. Engqvist, M.K., et al., GLYCOLATE OXIDASE3, a Glycolate Oxidase Homolog of Yeast l-Lactate Cytochrome c Oxidoreductase, Supports l-Lactate Oxidation in Roots of Arabidopsis. Plant Physiol, 2015. 169(2): p. 1042-61.

78. Hitomi, K., et al., Functional motifs in the (6-4) photolyase crystal structure make a comparative framework for DNA repair photolyases and clock cryptochromes. Proc Natl Acad Sci U S A, 2009. 106(17): p. 6962-7.

79. Lutziger, I. and D.J. Oliver, Molecular evidence of a unique lipoamide dehydrogenase in plastids: analysis of plastidic lipoamide dehydrogenase from Arabidopsis thaliana. FEBS Lett, 2000. 484(1): p. 12-6.

80. Steinbacher, S., et al., Crystal structure of the plant PPC decarboxylase AtHAL3a complexed with an ene-thiol reaction intermediate. J Mol Biol, 2003. 327(1): p. 193-202.

81. Choe, S., et al., The Arabidopsis dwarf1 mutant is defective in the conversion of 24-methylenecholesterol to campesterol in brassinosteroid biosynthesis. Plant Physiol, 1999. 119(3): p. 897-907.

82. Klahre, U., et al., The Arabidopsis DIMINUTO/DWARF1 gene encodes a protein involved in steroid synthesis. Plant Cell, 1998. 10(10): p. 1677-90.

83. Trivedi, D.K., et al., Genome-wide analysis of glutathione reductase (GR) genes from rice and Arabidopsis. Plant Signal Behav, 2013. 8(2): p. e23021.

84. Kataya, A.R. and S. Reumann, Arabidopsis glutathione reductase 1 is dually targeted to peroxisomes and the cytosol. Plant Signal Behav, 2010. 5(2): p. 171-5.

85. Sparla, F., et al., Cloning and heterologous expression of NAD(P)H:quinone reductase of Arabidopsis thaliana, a functional homologue of animal DT-diaphorase. FEBS Lett, 1999. 463(3): p. 382-6.

86. Marmagne, A., et al., Identification of new intrinsic proteins in Arabidopsis plasma membrane proteome. Mol Cell Proteomics, 2004. 3(7): p. 675-91.

87. Funck, D., S. Eckard, and G. Muller, Non-redundant functions of two proline dehydrogenase isoforms in Arabidopsis. BMC Plant Biol, 2010. 10: p. 70.

88. Nakashima, K., et al., A gene encoding proline dehydrogenase is not only induced by proline and hypoosmolarity, but is also developmentally regulated in the reproductive organs of Arabidopsis. Plant Physiol, 1998. 118(4): p. 1233-41.

89. Michalecka, A.M., et al., Arabidopsis genes encoding mitochondrial type II NAD(P)H dehydrogenases have different evolutionary origin and show distinct responses to light. Plant Physiol, 2003. 133(2): p. 642-52.

90. Daschner, K., I. Couee, and S. Binder, The mitochondrial isovaleryl-coenzyme a dehydrogenase of arabidopsis oxidizes intermediates of leucine and valine catabolism. Plant Physiol, 2001. 126(2): p. 601-12.

91. Daschner, K., et al., In plants a putative isovaleryl-CoA-dehydrogenase is located in mitochondria. Plant Mol Biol, 1999. 39(6): p. 1275-82.

92. Nakasako, M., et al., Structural basis of the LOV1 dimerization of Arabidopsis phototropins 1 and 2. J Mol Biol, 2008. 381(3): p. 718-33.

93. Leferink, N.G., W.A. van den Berg, and W.J. van Berkel, l-Galactono-gamma-lactone dehydrogenase from Arabidopsis thaliana, a flavoprotein involved in vitamin C biosynthesis. FEBS J, 2008. 275(4): p. 713-26.

94. Garcia, M.D., et al., Comprehensive understanding of acetohydroxyacid synthase inhibition by different herbicide families. Proc Natl Acad Sci U S A, 2017. 114(7): p. E1091-E1100.

95. Endo, M., et al., Molecular characterization of true and ectopic gene targeting events at the acetolactate synthase gene in Arabidopsis. Plant Cell Physiol, 2006. 47(3): p. 372-9.

96. Mackenzie, J., et al., Controlling electron transfer in Acyl-CoA oxidases and dehydrogenases: a structural view. J Biol Chem, 2006. 281(41): p. 31012-20.

97. Chew, O., J. Whelan, and A.H. Millar, Molecular definition of the ascorbate-glutathione cycle in Arabidopsis mitochondria reveals dual targeting of antioxidant defenses in plants. J Biol Chem, 2003. 278(47): p. 46869-77.

98. Hudig, M., et al., Plants Possess a Cyclic Mitochondrial Metabolic Pathway similar to the Mammalian Metabolic Repair Mechanism Involving Malate Dehydrogenase and l-2-Hydroxyglutarate Dehydrogenase. Plant Cell Physiol, 2015. 56(9): p. 1820-30.

99. Engqvist, M.K., et al., Mitochondrial 2-hydroxyglutarate metabolism. Mitochondrion, 2014. 19 Pt B: p. 275-81.

100. Roje, S., et al., Isolation, characterization, and functional expression of cDNAs encoding NADH-dependent methylenetetrahydrofolate reductase from higher plants. J Biol Chem, 1999. 274(51): p. 36089-96.

101. Lermontova, I., et al., Cloning and characterization of a plastidal and a mitochondrial isoform of tobacco protoporphyrinogen IX oxidase. Proc Natl Acad Sci U S A, 1997. 94(16): p. 8895-900.

102. Brautigam, C.A., et al., Structure of the photolyase-like domain of cryptochrome 1 from Arabidopsis thaliana. Proc Natl Acad Sci U S A, 2004. 101(33): p. 12142-7.

103. Lian, H.L., et al., Blue-light-dependent interaction of cryptochrome 1 with SPA1 defines a dynamic signaling mechanism. Genes Dev, 2011. 25(10): p. 1023-8.

104. Wu, G. and E.P. Spalding, Separate functions for nuclear and cytoplasmic cryptochrome 1 during photomorphogenesis of Arabidopsis seedlings. Proc Natl Acad Sci U S A, 2007. 104(47): p. 18813-8.

105. McQuinn, R.P., B. Wong, and J.J. Giovannoni, AtPDS overexpression in tomato: exposing unique patterns of carotenoid self-regulation and an alternative strategy for the enhancement of fruit carotenoid content. Plant Biotechnol J, 2018. 16(2): p. 482-494.

106. Aubourg, S., et al., Structure and expression of three src2 homologues and a novel subfamily of flavoprotein monooxygenase genes revealed by the analysis of a 25kb fragment from Arabidopsis thaliana chromosome IV. Gene, 1999. 230(2): p. 197-205.

107. Ishihara, H., et al., Primed histone demethylation regulates shoot regenerative competency. Nat Commun, 2019. 10(1): p. 1786.

108. Pedersen, L. and A. Henriksen, Acyl-CoA oxidase 1 from Arabidopsis thaliana. Structure of a key enzyme in plant lipid metabolism. J Mol Biol, 2005. 345(3): p. 487-500.

109. Pedersen, L. and A. Henriksen, Expression, purification and crystallization of two peroxisomal acyl-CoA oxidases from Arabidopsis thaliana. Acta Crystallogr D Biol Crystallogr, 2004. 60(Pt 6): p. 1125-8.

110. Elhafez, D., et al., Characterization of mitochondrial alternative NAD(P)H dehydrogenases in Arabidopsis: intraorganelle location and expression. Plant Cell Physiol, 2006. 47(1): p. 43-54.

111. Urban, P., et al., Cloning, yeast expression, and characterization of the coupling of two distantly related Arabidopsis thaliana NADPH-cytochrome P450 reductases with P450 CYP73A5. J Biol Chem, 1997. 272(31): p. 19176-86.

112. Ahou, A., et al., A plant spermine oxidase/dehydrogenase regulated by the proteasome and polyamines. J Exp Bot, 2014. 65(6): p. 1585-603.

113. Niu, G., et al., Structure of the Arabidopsis thaliana NADPH-cytochrome P450 reductase 2 (ATR2) provides insight into its function. FEBS J, 2017. 284(5): p. 754-765.

114. Picciocchi, A., R. Douce, and C. Alban, The plant biotin synthase reaction. Identification and characterization of essential mitochondrial accessory protein components. J Biol Chem, 2003. 278(27): p. 24966-75.

115. Takubo, K., et al., Identification and molecular characterization of mitochondrial ferredoxins and ferredoxin reductase from Arabidopsis. Plant Mol Biol, 2003. 52(4): p. 817-30.

116. Zarepour, M., et al., Xanthine dehydrogenase AtXDH1 from Arabidopsis thaliana is a potent producer of superoxide anions via its NADH oxidase activity. Plant Mol Biol, 2010. 72(3): p. 301-10.

117. Dai, S., et al., Crystal structure of Arabidopsis thaliana NADPH dependent thioredoxin reductase at 2.5 A resolution. J Mol Biol, 1996. 264(5): p. 1044-57.

118. Engqvist, M., et al., Two D-2-hydroxy-acid dehydrogenases in Arabidopsis thaliana with catalytic capacities to participate in the last reactions of the methylglyoxal and beta-oxidation pathways. J Biol Chem, 2009. 284(37): p. 25026-37.

119. Pose, D., et al., Identification of the Arabidopsis dry2/sqe1-5 mutant reveals a central role for sterols in drought tolerance and regulation of reactive oxygen species. Plant J, 2009. 59(1): p. 63-76.

120. Jamai, A., et al., Arabidopsis photorespiratory serine hydroxymethyltransferase activity requires the mitochondrial accumulation of ferredoxin-dependent glutamate synthase. Plant Cell, 2009. 21(2): p. 595-606.

121. Bari, R., et al., A glycolate dehydrogenase in the mitochondria of Arabidopsis thaliana. J Exp Bot, 2004. 55(397): p. 623-30.

122. Kuhn, K., et al., Complete Mitochondrial Complex I Deficiency Induces an Up-Regulation of Respiratory Fluxes That Is Abolished by Traces of Functional Complex I. Plant Physiol, 2015. 168(4): p. 1537-49.

123. Fatihi, A., et al., A Dedicated Type II NADPH Dehydrogenase Performs the Penultimate Step in the Biosynthesis of Vitamin K1 in Synechocystis and Arabidopsis. Plant Cell, 2015. 27(6): p. 1730-41.

124. Eugeni Piller, L., et al., Chloroplast lipid droplet type II NAD(P)H quinone oxidoreductase is essential for prenylquinone metabolism and vitamin K1 accumulation. Proc Natl Acad Sci U S A, 2011. 108(34): p. 14354-9.

125. Shen, J., et al., An in vivo expression system for the identification of cargo proteins of vacuolar sorting receptors in Arabidopsis culture cells. Plant J, 2013. 75(6): p. 1003-17.

126. Sagor, G.H., et al., Reducing Cytoplasmic Polyamine Oxidase Activity in Arabidopsis Increases Salt and Drought Tolerance by Reducing Reactive Oxygen Species Production and Increasing Defense Gene Expression. Front Plant Sci, 2016. 7: p. 214.

127. Hao, J., et al., Characterization of l-aspartate oxidase from Arabidopsis thaliana. Plant Sci, 2018. 271: p. 133-142.

128. Katoh, A., et al., Early steps in the biosynthesis of NAD in Arabidopsis start with aspartate and occur in the plastid. Plant Physiol, 2006. 141(3): p. 851-7.

129. Kumar, R., et al., A mutation in Arabidopsis cytochrome b5 reductase identified by high-throughput screening differentially affects hydroxylation and desaturation. Plant J, 2006. 48(6): p. 920-32.

130. Duncan, O., et al., Multiple lines of evidence localize signaling, morphology, and lipid biosynthesis machinery to the mitochondrial outer membrane of Arabidopsis. Plant Physiol, 2011. 157(3): p. 1093-113.

131. Kruft, V., et al., Proteomic approach to identify novel mitochondrial proteins in Arabidopsis. Plant Physiol, 2001. 127(4): p. 1694-710.

132. Bae, E., et al., Crystal structure of Arabidopsis thaliana cytokinin dehydrogenase. Proteins, 2008. 70(1): p. 303-6.

133. Ullrich, A., et al., Plant dihydroorotate dehydrogenase differs significantly in substrate specificity and inhibition from the animal enzymes. FEBS Lett, 2002. 529(2-3): p. 346-50.

134. Klar, T., et al., Cryptochrome 3 from Arabidopsis thaliana: structural and functional analysis of its complex with a folate light antenna. J Mol Biol, 2007. 366(3): p. 954-64.

135. Kleine, T., P. Lockhart, and A. Batschauer, An Arabidopsis protein closely related to Synechocystis cryptochrome is targeted to organelles. Plant J, 2003. 35(1): p. 93-103.

136. Kim, J.I., et al., yucca6, a dominant mutation in Arabidopsis, affects auxin accumulation and auxin-related phenotypes. Plant Physiol, 2007. 145(3): p. 722-35.

137. Ishizaki, K., et al., The mitochondrial electron transfer flavoprotein complex is essential for survival of Arabidopsis in extended darkness. Plant J, 2006. 47(5): p. 751-60.

138. Daniel, B., et al., Structure of a Berberine Bridge Enzyme-Like Enzyme with an Active Site Specific to the Plant Family Brassicaceae. PLoS One, 2016. 11(6): p. e0156892.

139. Sang, Y., et al., Identification of a pyridoxine (pyridoxamine) 5'-phosphate oxidase from Arabidopsis thaliana. FEBS Lett, 2007. 581(3): p. 344-8.

140. Sang, Y., et al., Expression, in vivo localization and phylogenetic analysis of a pyridoxine 5'-phosphate oxidase in Arabidopsis thaliana. Plant Physiol Biochem, 2011. 49(1): p. 88-95.

141. Lancien, M., et al., Arabidopsis glt1-T mutant defines a role for NADH-GOGAT in the non-photorespiratory ammonium assimilatory pathway. Plant J, 2002. 29(3): p. 347-58.

142. Zybailov, B., et al., Sorting signals, N-terminal modifications and abundance of the chloroplast proteome. PLoS One, 2008. 3(4): p. e1994.

143. Laskowski, M.J., et al., FQR1, a novel primary auxin-response gene, encodes a flavin mononucleotide-binding quinone reductase. Plant Physiol, 2002. 128(2): p. 578-90.

144. Pudasaini, A., et al., Kinetics of the LOV domain of ZEITLUPE determine its circadian function in Arabidopsis. Elife, 2017. 6.

145. Harada, A., T. Sakai, and K. Okada, Phot1 and phot2 mediate blue light-induced transient increases in cytosolic Ca2+ differently in Arabidopsis leaves. Proc Natl Acad Sci U S A, 2003. 100(14): p. 8583-8.

146. Huizinga, D.H., et al., Farnesylcysteine lyase is involved in negative regulation of abscisic acid signaling in Arabidopsis. Mol Plant, 2010. 3(1): p. 143-55.

147. Hooks, M.A., F. Kellas, and I.A. Graham, Long-chain acyl-CoA oxidases of Arabidopsis. Plant J, 1999. 20(1): p. 1-13.

148. Barrero, J.M., et al., A mutational analysis of the ABA1 gene of Arabidopsis thaliana highlights the involvement of ABA in vegetative development. J Exp Bot, 2005. 56(418): p. 2071-83.
