# Supplementary figures and images for: The Flavoproteome of the Model Plant Arabidopsis thaliana
Source: Int J Mol Sci. 2020 Jul 28;21(15):5371. doi: 10.3390/ijms21155371 (PMC7432721; doi:10.3390/ijms21155371)

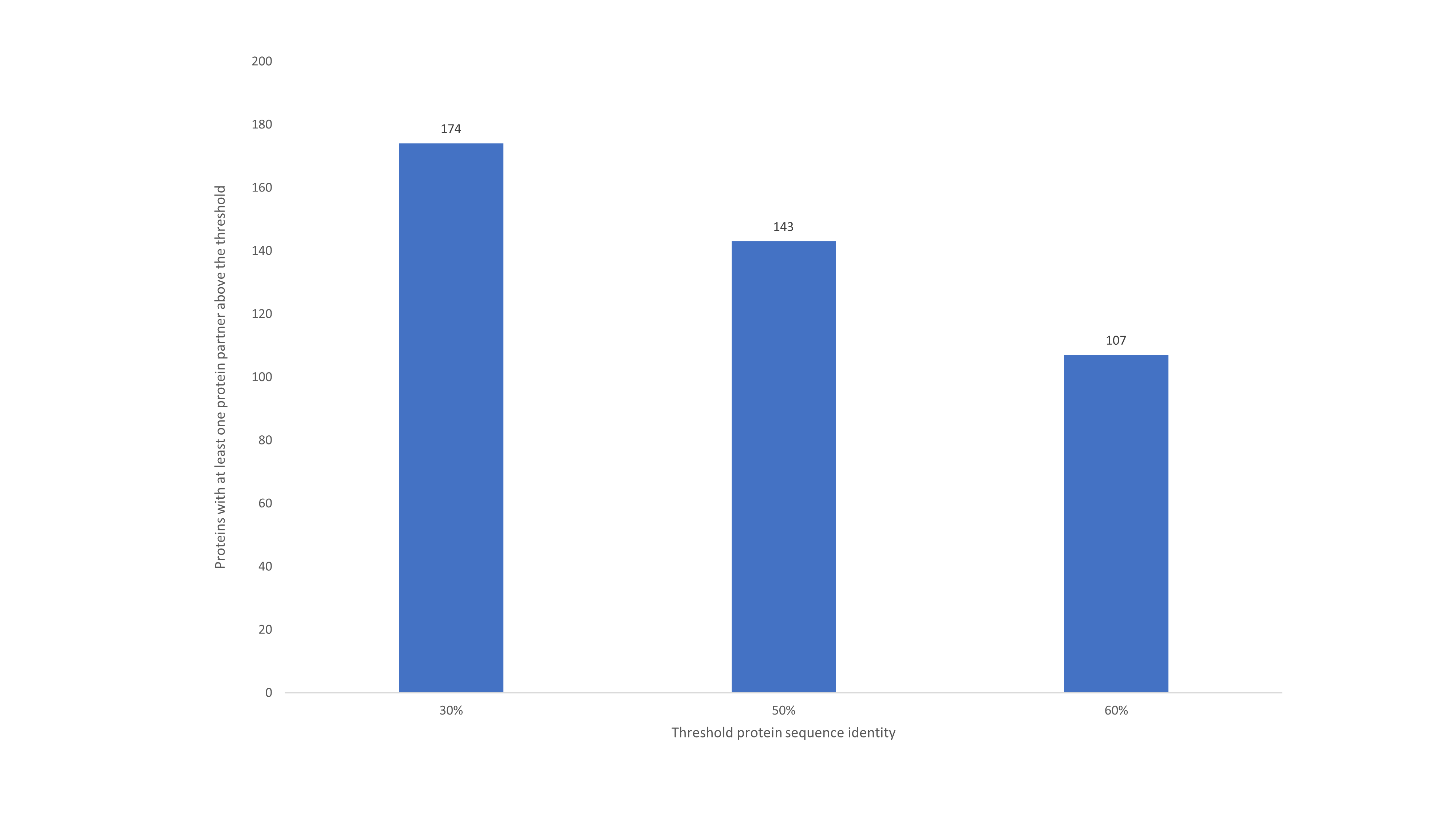

Supplement: Supplementary file 1 [file ijms-21-05371-s001.zip › Figure S1.tif]

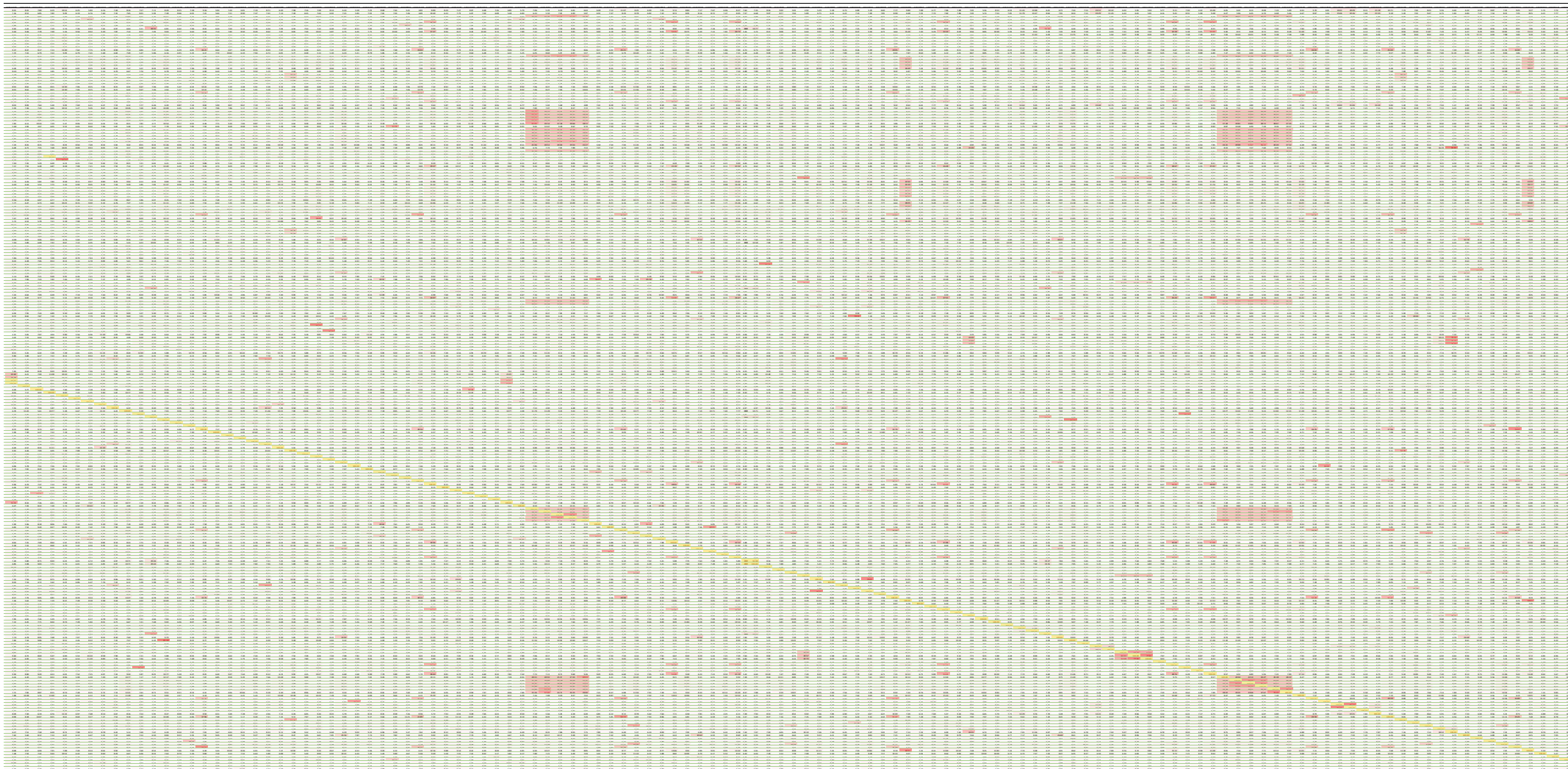

|      |      |      |
|------|------|------|
| 1    | 2    | 3    |
| 4    | 5    | 6    |
| 7    | 8    | 9    |
| 10   | 11   | 12   |
| 13   | 14   | 15   |
| 16   | 17   | 18   |
| 19   | 20   | 21   |
| 22   | 23   | 24   |
| 25   | 26   | 27   |
| 28   | 29   | 30   |
| 31   | 32   | 33   |
| 34   | 35   | 36   |
| 37   | 38   | 39   |
| 40   | 41   | 42   |
| 43   | 44   | 45   |
| 46   | 47   | 48   |
| 49   | 50   | 51   |
| 52   | 53   | 54   |
| 55   | 56   | 57   |
| 58   | 59   | 60   |
| 61   | 62   | 63   |
| 64   | 65   | 66   |
| 67   | 68   | 69   |
| 70   | 71   | 72   |
| 73   | 74   | 75   |
| 76   | 77   | 78   |
| 79   | 80   | 81   |
| 82   | 83   | 84   |
| 85   | 86   | 87   |
| 88   | 89   | 90   |
| 91   | 92   | 93   |
| 94   | 95   | 96   |
| 97   | 98   | 99   |
| 100  | 101  | 102  |
| 103  | 104  | 105  |
| 106  | 107  | 108  |
| 109  | 110  | 111  |
| 112  | 113  | 114  |
| 115  | 116  | 117  |
| 118  | 119  | 120  |
| 121  | 122  | 123  |
| 124  | 125  | 126  |
| 127  | 128  | 129  |
| 130  | 131  | 132  |
| 133  | 134  | 135  |
| 136  | 137  | 138  |
| 139  | 140  | 141  |
| 142  | 143  | 144  |
| 145  | 146  | 147  |
| 148  | 149  | 150  |
| 151  | 152  | 153  |
| 154  | 155  | 156  |
| 157  | 158  | 159  |
| 160  | 161  | 162  |
| 163  | 164  | 165  |
| 166  | 167  | 168  |
| 169  | 170  | 171  |
| 172  | 173  | 174  |
| 175  | 176  | 177  |
| 178  | 179  | 180  |
| 181  | 182  | 183  |
| 184  | 185  | 186  |
| 187  | 188  | 189  |
| 190  | 191  | 192  |
| 193  | 194  | 195  |
| 196  | 197  | 198  |
| 199  | 200  | 201  |
| 202  | 203  | 204  |
| 205  | 206  | 207  |
| 208  | 209  | 210  |
| 211  | 212  | 213  |
| 214  | 215  | 216  |
| 217  | 218  | 219  |
| 220  | 221  | 222  |
| 223  | 224  | 225  |
| 226  | 227  | 228  |
| 229  | 230  | 231  |
| 232  | 233  | 234  |
| 235  | 236  | 237  |
| 238  | 239  | 240  |
| 241  | 242  | 243  |
| 244  | 245  | 246  |
| 247  | 248  | 249  |
| 250  | 251  | 252  |
| 253  | 254  | 255  |
| 256  | 257  | 258  |
| 259  | 260  | 261  |
| 262  | 263  | 264  |
| 265  | 266  | 267  |
| 268  | 269  | 270  |
| 271  | 272  | 273  |
| 274  | 275  | 276  |
| 277  | 278  | 279  |
| 280  | 281  | 282  |
| 283  | 284  | 285  |
| 286  | 287  | 288  |
| 289  | 290  | 291  |
| 292  | 293  | 294  |
| 295  | 296  | 297  |
| 298  | 299  | 300  |
| 301  | 302  | 303  |
| 304  | 305  | 306  |
| 307  | 308  | 309  |
| 310  | 311  | 312  |
| 313  | 314  | 315  |
| 316  | 317  | 318  |
| 319  | 320  | 321  |
| 322  | 323  | 324  |
| 325  | 326  | 327  |
| 328  | 329  | 330  |
| 331  | 332  | 333  |
| 334  | 335  | 336  |
| 337  | 338  | 339  |
| 340  | 341  | 342  |
| 343  | 344  | 345  |
| 346  | 347  | 348  |
| 349  | 350  | 351  |
| 352  | 353  | 354  |
| 355  | 356  | 357  |
| 358  | 359  | 360  |
| 361  | 362  | 363  |
| 364  | 365  | 366  |
| 367  | 368  | 369  |
| 370  | 371  | 372  |
| 373  | 374  | 375  |
| 376  | 377  | 378  |
| 379  | 380  | 381  |
| 382  | 383  | 384  |
| 385  | 386  | 387  |
| 388  | 389  | 390  |
| 391  | 392  | 393  |
| 394  | 395  | 396  |
| 397  | 398  | 399  |
| 400  | 401  | 402  |
| 403  | 404  | 405  |
| 406  | 407  | 408  |
| 409  | 410  | 411  |
| 412  | 413  | 414  |
| 415  | 416  | 417  |
| 418  | 419  | 420  |
| 421  | 422  | 423  |
| 424  | 425  | 426  |
| 427  | 428  | 429  |
| 430  | 431  | 432  |
| 433  | 434  | 435  |
| 436  | 437  | 438  |
| 439  | 440  | 441  |
| 442  | 443  | 444  |
| 445  | 446  | 447  |
| 448  | 449  | 450  |
| 451  | 452  | 453  |
| 454  | 455  | 456  |
| 457  | 458  | 459  |
| 460  | 461  | 462  |
| 463  | 464  | 465  |
| 466  | 467  | 468  |
| 469  | 470  | 471  |
| 472  | 473  | 474  |
| 475  | 476  | 477  |
| 478  | 479  | 480  |
| 481  | 482  | 483  |
| 484  | 485  | 486  |
| 487  | 488  | 489  |
| 490  | 491  | 492  |
| 493  | 494  | 495  |
| 496  | 497  | 498  |
| 499  | 500  | 501  |
| 502  | 503  | 504  |
| 505  | 506  | 507  |
| 508  | 509  | 510  |
| 511  | 512  | 513  |
| 514  | 515  | 516  |
| 517  | 518  | 519  |
| 520  | 521  | 522  |
| 523  | 524  | 525  |
| 526  | 527  | 528  |
| 529  | 530  | 531  |
| 532  | 533  | 534  |
| 535  | 536  | 537  |
| 538  | 539  | 540  |
| 541  | 542  | 543  |
| 544  | 545  | 546  |
| 547  | 548  | 549  |
| 550  | 551  | 552  |
| 553  | 554  | 555  |
| 556  | 557  | 558  |
| 559  | 560  | 561  |
| 562  | 563  | 564  |
| 565  | 566  | 567  |
| 568  | 569  | 570  |
| 571  | 572  | 573  |
| 574  | 575  | 576  |
| 577  | 578  | 579  |
| 580  | 581  | 582  |
| 583  | 584  | 585  |
| 586  | 587  | 588  |
| 589  | 590  | 591  |
| 592  | 593  | 594  |
| 595  | 596  | 597  |
| 598  | 599  | 600  |
| 601  | 602  | 603  |
| 604  | 605  | 606  |
| 607  | 608  | 609  |
| 610  | 611  | 612  |
| 613  | 614  | 615  |
| 616  | 617  | 618  |
| 619  | 620  | 621  |
| 622  | 623  | 624  |
| 625  | 626  | 627  |
| 628  | 629  | 630  |
| 631  | 632  | 633  |
| 634  | 635  | 636  |
| 637  | 638  | 639  |
| 640  | 641  | 642  |
| 643  | 644  | 645  |
| 646  | 647  | 648  |
| 649  | 650  | 651  |
| 652  | 653  | 654  |
| 655  | 656  | 657  |
| 658  | 659  | 660  |
| 661  | 662  | 663  |
| 664  | 665  | 666  |
| 667  | 668  | 669  |
| 670  | 671  | 672  |
| 673  | 674  | 675  |
| 676  | 677  | 678  |
| 679  | 680  | 681  |
| 682  | 683  | 684  |
| 685  | 686  | 687  |
| 688  | 689  | 690  |
| 691  | 692  | 693  |
| 694  | 695  | 696  |
| 697  | 698  | 699  |
| 700  | 701  | 702  |
| 703  | 704  | 705  |
| 706  | 707  | 708  |
| 709  | 710  | 711  |
| 712  | 713  | 714  |
| 715  | 716  | 717  |
| 718  | 719  | 720  |
| 721  | 722  | 723  |
| 724  | 725  | 726  |
| 727  | 728  | 729  |
| 730  | 731  | 732  |
| 733  | 734  | 735  |
| 736  | 737  | 738  |
| 739  | 740  | 741  |
| 742  | 743  | 744  |
| 745  | 746  | 747  |
| 748  | 749  | 750  |
| 751  | 752  | 753  |
| 754  | 755  | 756  |
| 757  | 758  | 759  |
| 760  | 761  | 762  |
| 763  | 764  | 765  |
| 766  | 767  | 768  |
| 769  | 770  | 771  |
| 772  | 773  | 774  |
| 775  | 776  | 777  |
| 778  | 779  | 780  |
| 781  | 782  | 783  |
| 784  | 785  | 786  |
| 787  | 788  | 789  |
| 790  | 791  | 792  |
| 793  | 794  | 795  |
| 796  | 797  | 798  |
| 799  | 800  | 801  |
| 802  | 803  | 804  |
| 805  | 806  | 807  |
| 808  | 809  | 810  |
| 811  | 812  | 813  |
| 814  | 815  | 816  |
| 817  | 818  | 819  |
| 820  | 821  | 822  |
| 823  | 824  | 825  |
| 826  | 827  | 828  |
| 829  | 830  | 831  |
| 832  | 833  | 834  |
| 835  | 836  | 837  |
| 838  | 839  | 840  |
| 841  | 842  | 843  |
| 844  | 845  | 846  |
| 847  | 848  | 849  |
| 850  | 851  | 852  |
| 853  | 854  | 855  |
| 856  | 857  | 858  |
| 859  | 860  | 861  |
| 862  | 863  | 864  |
| 865  | 866  | 867  |
| 868  | 869  | 870  |
| 871  | 872  | 873  |
| 874  | 875  | 876  |
| 877  | 878  | 879  |
| 880  | 881  | 882  |
| 883  | 884  | 885  |
| 886  | 887  | 888  |
| 889  | 890  | 891  |
| 892  | 893  | 894  |
| 895  | 896  | 897  |
| 898  | 899  | 900  |
| 901  | 902  | 903  |
| 904  | 905  | 906  |
| 907  | 908  | 909  |
| 910  | 911  | 912  |
| 913  | 914  | 915  |
| 916  | 917  | 918  |
| 919  | 920  | 921  |
| 922  | 923  | 924  |
| 925  | 926  | 927  |
| 928  | 929  | 930  |
| 931  | 932  | 933  |
| 934  | 935  | 936  |
| 937  | 938  | 939  |
| 940  | 941  | 942  |
| 943  | 944  | 945  |
| 946  | 947  | 948  |
| 949  | 950  | 951  |
| 952  | 953  | 954  |
| 955  | 956  | 957  |
| 958  | 959  | 960  |
| 961  | 962  | 963  |
| 964  | 965  | 966  |
| 967  | 968  | 969  |
| 970  | 971  | 972  |
| 973  | 974  | 975  |
| 976  | 977  | 978  |
| 979  | 980  | 981  |
| 982  | 983  | 984  |
| 985  | 986  | 987  |
| 988  | 989  | 990  |
| 991  | 992  | 993  |
| 994  | 995  | 996  |
| 997  | 998  | 999  |
| 1000 | 1001 | 1002 |

Supplement: Supplementary file 1 [file ijms-21-05371-s001.zip › Table S2.pdf]
